# The associations of major foods and fibre with risks of ischaemic and haemorrhagic stroke: a prospective study of 418 329 participants in the EPIC cohort across nine European countries

**DOI:** 10.1093/eurheartj/ehaa007

**Published:** 2020-02-24

**Authors:** Tammy Y N Tong, Paul N Appleby, Timothy J Key, Christina C Dahm, Kim Overvad, Anja Olsen, Anne Tjønneland, Verena Katzke, Tilman Kühn, Heiner Boeing, Anna Karakatsani, Eleni Peppa, Antonia Trichopoulou, Elisabete Weiderpass, Giovanna Masala, Sara Grioni, Salvatore Panico, Rosario Tumino, Jolanda M A Boer, W M Monique Verschuren, J Ramón Quirós, Antonio Agudo, Miguel Rodríguez-Barranco, Liher Imaz, María-Dolores Chirlaque, Conchi Moreno-Iribas, Gunnar Engström, Emily Sonestedt, Marcus Lind, Julia Otten, Kay-Tee Khaw, Dagfinn Aune, Elio Riboli, Nicholas J Wareham, Fumiaki Imamura, Nita G Forouhi, Emanuele di Angelantonio, Angela M Wood, Adam S Butterworth, Aurora Perez-Cornago

**Affiliations:** Cancer Epidemiology Unit, Nuffield Department of Population Health, University of Oxford, Richard Doll Building, Roosevelt Drive, Oxford OX3 7LF, UK; Cancer Epidemiology Unit, Nuffield Department of Population Health, University of Oxford, Richard Doll Building, Roosevelt Drive, Oxford OX3 7LF, UK; Cancer Epidemiology Unit, Nuffield Department of Population Health, University of Oxford, Richard Doll Building, Roosevelt Drive, Oxford OX3 7LF, UK; Department of Public Health, Aarhus University, Nordre Ringgade 1, 8000 Aarhus, Demark; Department of Public Health, Aarhus University, Nordre Ringgade 1, 8000 Aarhus, Demark; Department of Cardiology, Aalborg University Hospital, Reberbansgade 15, 9000 Aalborg, Denmark; Diet, Genes and Environment, Danish Cancer Society Research Center, Copenhagen, Denmark; Diet, Genes and Environment, Danish Cancer Society Research Center, Copenhagen, Denmark; Department of Public Health, Faculty of Health and Medical Sciences, University of Copenhagen, Strandboulevarden 49, 2100 Copenhagen, Denmark; Division of Cancer Epidemiology, German Cancer Research Center (DKFZ), Im Neuenheimer Feld 580, 69120 Heidelberg Germany; Division of Cancer Epidemiology, German Cancer Research Center (DKFZ), Im Neuenheimer Feld 580, 69120 Heidelberg Germany; Department of Epidemiology, German Institute of Human Nutrition (DIfE) Postdam-Rehbrücke, Arthur-Scheunert-Allee 114, 14558 Nuthetal, Germany; Hellenic Health Foundation, Kaisareias 13 & Alexandroupoleos, 11527 Athens, Greece; 2nd Pulmonary Medicine Department, School of Medicine, National and Kapodistrian University of Athens, “ATTIKON” University Hospital, 1, Rimini Str, Haidari, 12462 Athens, Greece; Hellenic Health Foundation, Kaisareias 13 & Alexandroupoleos, 11527 Athens, Greece; Hellenic Health Foundation, Kaisareias 13 & Alexandroupoleos, 11527 Athens, Greece; International Agency for Research on Cancer (IARC), World Health Organization (WHO), 150 Cours Albert Thomas, 69372, Lyon CEDEX 08, France; Cancer Risk Factors and Life-Style Epidemiology Unit, Institute for Cancer Research, Prevention and Clinical Network - ISPRO, Via Cosimo Il Vecchio; Epidemiology and Prevention Unit, Fondazione IRCCS Istituto Nazionale dei Tumori di Milano, Via Giacomo Venezian, 1, 20133 Milan, Italy; Dipartimento Di Medicina Clinica E Chirurgia Federico II University, Corso Umberto I, 40, 80138 Naples, Italy; Cancer Registry and Histopathology Department, “M.P.Arezzo” Hospital, ASP Contrada Rito, 97100 Ragusa, Italy; National Institute for Public Health and the Environment, Antonie van Leeuwenhoeklaan 9, 3721 MA Bilthoven, the Netherlands; National Institute for Public Health and the Environment, Antonie van Leeuwenhoeklaan 9, 3721 MA Bilthoven, the Netherlands; Julius Center for Health Sciences and Primary Care, University Medical Center Utrecht, Utrecht University, Heidelberglaan 100, 3584 Utrecht, the Netherlands; Public Health Directorate, General Elorza 32, 33001 Oviedo, Asturias, Spain; Unit of Nutrition and Cancer. Cancer Epidemiology Research Program. Catalan Institute of Oncology-IDIBELL. Avinguda de la Gran Via de l'Hospitalet 199-203, 08908 L'Hospitalet de Llobregat, Barcelona, Spain; Andalusian School of Public Health. Biomedical Research Institute ibs.GRANADA, University of Granada, Cuesta del Observatorio, 4, 18011 Granada, Spain; CIBER of Epidemiology and Public Health. Av. Monforte de Lemos, 3-5. Pabellón 11. Planta 0 28029 Madrid, Spain; Ministry of Health of the Basque Government, Public Health Division of Gipuzkoa, Andia 13, 20004 Donostia-San Sebastian, Spain; Biodonostia Health Research Institute, Paseo Doctor Begiristain, s/n, 20014 Donostia-San Sebastian, Spain; CIBER of Epidemiology and Public Health. Av. Monforte de Lemos, 3-5. Pabellón 11. Planta 0 28029 Madrid, Spain; Department of Epidemiology, Regional Health Council, IMIB-Arrixaca, Murcia University, Ronda de Levante, 11, 30008 Murcia, Spain; Instituto de Salud Pública de Navarra, IdiSNA, Navarre Institute for Health Research, Calle de Irunlarrea 3, 31008 Pamplona, Spain; Red de Investigación en Servicios de Salud en Enfermedades Crónicas (REDISSEC), Recinto Hospitalario de Navarra, Calle de Irunlarrea s/n, 31621 Pamplona, Spain; Department of Clinical Science in Malmö, Lund University, Bergsgatan 31 B, 21445 Malmö, Sweden; Nutritional Epidemiology, Department of Clinical Sciences Malmö, Lund University, Jan Waldenströms gata 35, CRC, hus 60 plan 13 205 02 Malmö, Sweden; Department of Public Health and Clinical Medicine, Umeå University, 901 87 Umeå, Sweden; Department of Public Health and Clinical Medicine, Umeå University, 901 87 Umeå, Sweden; Department of Public Health and Primary Care, University of Cambridge, Strangeways Research Laboratory, Worts Causeway, Cambridge CB1 8RN, UK; Department of Epidemiology and Biostatistics, School of Public Health, Imperial College London, South Kensington Campus, London SW7 2AZ, UK; Department of Nutrition, Bjørknes University College, Lovisenberggata 13, N- 0456 Oslo, Norway; Department of Endocrinology, Morbid Obesity and Preventive Medicine, Oslo University Hospital Ullevål, OUS HF Aker sykehus, Postboks 4959 Nydalen, 0424 Oslo, Norway; Department of Epidemiology and Biostatistics, School of Public Health, Imperial College London, South Kensington Campus, London SW7 2AZ, UK; MRC Epidemiology Unit, University of Cambridge School of Clinical Medicine, Cambridge Biomedical Campus, Cambridge CB2 0QQ, UK; MRC Epidemiology Unit, University of Cambridge School of Clinical Medicine, Cambridge Biomedical Campus, Cambridge CB2 0QQ, UK; MRC Epidemiology Unit, University of Cambridge School of Clinical Medicine, Cambridge Biomedical Campus, Cambridge CB2 0QQ, UK; MRC/BHF Cardiovascular Epidemiology Unit, Department of Public Health and Primary Care, University of Cambridge, Strangeways Research Laboratory, Wort's Causeway, Cambridge CB1 8RN, UK; MRC/BHF Cardiovascular Epidemiology Unit, Department of Public Health and Primary Care, University of Cambridge, Strangeways Research Laboratory, Wort's Causeway, Cambridge CB1 8RN, UK; MRC/BHF Cardiovascular Epidemiology Unit, Department of Public Health and Primary Care, University of Cambridge, Strangeways Research Laboratory, Wort's Causeway, Cambridge CB1 8RN, UK; Cancer Epidemiology Unit, Nuffield Department of Population Health, University of Oxford, Richard Doll Building, Roosevelt Drive, Oxford OX3 7LF, UK

**Keywords:** Diet, Fruit, Vegetables, Fibre, Ischaemic stroke, Haemorrhagic stroke

## Abstract

**Aim:**

To investigate the associations between major foods and dietary fibre with subtypes of stroke in a large prospective cohort.

**Methods and results:**

We analysed data on 418 329 men and women from nine European countries, with an average of 12.7 years of follow-up. Diet was assessed using validated country-specific questionnaires which asked about habitual intake over the past year, calibrated using 24-h recalls. Multivariable-adjusted Cox regressions were used to estimate hazard ratios (HRs) for ischaemic and haemorrhagic stroke associated with consumption of red and processed meat, poultry, fish, dairy foods, eggs, cereals, fruit and vegetables, legumes, nuts and seeds, and dietary fibre. For ischaemic stroke (4281 cases), lower risks were observed with higher consumption of fruit and vegetables combined (HR; 95% CI per 200 g/day higher intake, 0.87; 0.82–0.93, *P*-trend < 0.001), dietary fibre (per 10 g/day, 0.77; 0.69–0.86, *P*-trend < 0.001), milk (per 200 g/day, 0.95; 0.91–0.99, *P*-trend = 0.02), yogurt (per 100 g/day, 0.91; 0.85–0.97, *P*-trend = 0.004), and cheese (per 30 g/day, 0.88; 0.81–0.97, *P*-trend = 0.008), while higher risk was observed with higher red meat consumption which attenuated when adjusted for the other statistically significant foods (per 50 g/day, 1.07; 0.96–1.20, *P*-trend = 0.20). For haemorrhagic stroke (1430 cases), higher risk was associated with higher egg consumption (per 20 g/day, 1.25; 1.09–1.43, *P*-trend = 0.002).

**Conclusion:**

Risk of ischaemic stroke was inversely associated with consumption of fruit and vegetables, dietary fibre, and dairy foods, while risk of haemorrhagic stroke was positively associated with egg consumption. The apparent differences in the associations highlight the importance of examining ischaemic and haemorrhagic stroke subtypes separately.

**See page 2641 for the editorial comment on this article (doi: 10.1093/eurheartj/ehaa317)**

## Introduction 

In 2013, stroke was the second most common cause of death and the third most common cause of disability worldwide.^1^ Although the age-standardized incidence and mortality of stroke have decreased globally in the past two decades, the absolute numbers of both ischaemic and haemorrhagic stroke cases, deaths and prevalence have increased.^2^ Therefore, the primary prevention of stroke is of utmost importance. Emerging evidence has also shown that the two main types of stroke (ischaemic and haemorrhagic), which have different pathologies, might also differ in some risk factors.^3–5^ For example, recent evidence including observational, genetic, and trial data has suggested that lower low-density lipoprotein cholesterol concentrations may be causally associated with lower risks of ischaemic stroke, but higher risks of haemorrhagic stroke.^3^ A recent large observational study also showed positive associations of obesity and diabetes with ischaemic stroke, but inverse associations with haemorrhagic stroke.^5^ Therefore, stroke subtypes should be examined separately.

Prior evidence suggests that diet might be related to stroke risk, but most previous prospective studies have focused on total stroke (i.e. ischaemic, haemorrhagic, and unspecified combined) instead of examining the risks of ischaemic and haemorrhagic stroke separately.^6–8^ Evidence on diet and haemorrhagic stroke in particular is relatively limited possibly due to smaller numbers of cases in most prospective studies.^8–11^ Some previous prospective studies have shown a positive association between red or processed meat consumption and risk of total or ischaemic stroke , ^9^ ^,^ ^12–14^ and an inverse association for fruit and vegetables,^11^ ^,^ ^15^ and dietary fibre.^16–20^ There is less evidence for most other major foods including fish, dairy products, eggs, cereals, legumes, or nuts and seeds,^8^ hence further evidence from large cohorts is needed.

The aim of this study was to assess the associations of major foods and dietary fibre with risk of ischaemic and haemorrhagic stroke in a large European cohort (the European Prospective Investigation into Cancer and Nutrition, EPIC) of over 400 000 men and women.

## Methods

### Study population

The study population included 418 329 men and women from 22 centres in nine European countries (Denmark, Germany, Greece, Italy, the Netherlands, Norway, Spain, Sweden, and the UK), who were recruited for the EPIC study between 1992 and 2000. The exclusion criteria for this current study are listed in the Supplementary material online, *Methods*. The rationale and details of the study design have been described previously.^21^ ^,^ ^22^ In brief, participants in EPIC completed country-specific dietary and lifestyle questionnaires at recruitment, which asked about socio-demographic characteristics, habitual diets, lifestyle factors, and medical history. Further details on data collection for physical activity, anthropometry, blood pressure (available in most of the cohort), and blood cholesterol (available in a subset^23^ ^,^ ^24^) are included in the Supplementary material online, *Methods*. The baseline data were centralized at the World Health Organization’s International Agency for Research on Cancer (IARC) in Lyon, France. All participants gave written informed consent, and the study protocol was approved by the ethical review boards of IARC and the institutions where participants were recruited.^22^

### Dietary assessment

Dietary intake was assessed using country-specific questionnaires, mostly food frequency questionnaires, which asked about dietary intake during the year before enrolment.^22^ Based on responses to the questionnaires, we estimated intakes of major food groups and subgroups including meat and meat products (red meat, processed meat, and poultry), fish and fish products (white fish and fatty fish), dairy products (including milk, yogurt, cheese), eggs, cereals and cereal products, fruit and vegetables (combined and separately), legumes, nuts and seeds, and dietary fibre (total fibre and cereal, fruit, and vegetable fibre). Details of examples of foods included in each food group, and data availability of each food group, are included as Supplementary material online, *Table S1*. In addition, a stratified random sample of 8% of participants across all centres also completed a standardized and computerized 24-h recall, which was used in our study for calibration purposes to reduce between-centre heterogeneity.^25^ The calibration process is described in greater detail in the Supplementary material online, *Methods*.

### Outcome assessment

The outcomes of interest were ischaemic (ICD9 433–434 or ICD10 I63) and haemorrhagic stroke (ICD9 430–431 or ICD10 I60–I61) as the two primary endpoints, and secondarily total stroke (ICD9 430–431, 433–434, 436 or ICD10 I60–I61, I63–I64). Both fatal and non-fatal incident events were considered. Details of the ascertainment of case status are included in the Supplementary material online, *Methods*. The last date of follow-up varied between 2003 and 2012 across the different centres (details in Supplementary material online, *Table S2*).

### Statistical analyses

Using Cox proportional hazards regression, we calculated hazard ratios (HRs) and 95% confidence intervals (CIs) for each food group or fibre intake (by per unit difference or fifths of intakes) with risks of ischaemic, haemorrhagic, and total stroke. All our analyses were adjusted for age, smoking status and number of cigarettes per day, self-reported history of diabetes, hypertension, or hyperlipidaemia, Cambridge physical activity index,^26^ employment status, level of education completed, current alcohol consumption, body mass index, and calibrated or observed intake of energy, as appropriate, and stratified by sex and EPIC centre. Details on unit sizes and categorization of exposures and covariates can be found in the Supplementary material online, *Methods*. The proportional hazards assumption was assessed on the basis of Schoenfeld residuals for the main exposures (i.e. calibrated intakes of foods and fibre), and was not violated in the adjusted models for either ischaemic or haemorrhagic stroke (*P* > 0.05 in all models).

For each dietary exposure that was significantly associated with risk of ischaemic or haemorrhagic stroke risk, we estimated the absolute rate difference in incidence of the outcome per unit difference in the exposure, based on a previously reported method.^27^ Rate difference for each dietary exposure was estimated based on the average incidence of ischaemic or haemorrhagic stroke in the overall cohort, and 95% confidence intervals were estimated using a bootstrap method with 1000 resamplings.^28^

To investigate whether the observed associations were independent, we included a model mutually adjusting for all significant foods from the main analyses (details in Supplementary material online, *Methods*). Because fibre is a common component of several foods of interest and has been shown in previous studies^20^ to be inversely associated with total and ischaemic stroke, we additionally examined the associations of all foods further adjusting for dietary fibre; to avoid over-adjustment, cereal fibre was adjusted for in analyses of risk for fruit and vegetables, and fruit and vegetable fibre was adjusted for in analyses of risk for cereals and cereal products. An additional model was included mutually adjusting for all food groups regardless of their associations, restricted to centres with data on all foods. Further *post hoc* analyses included examination of more detailed subtypes of fruits and vegetables, and the associations of the main exposures with subtypes of haemorrhagic stroke (i.e. subarachnoid and intracerebral haemorrhage).

To examine the possible roles of blood pressure and blood cholesterol in driving the relationships, we estimated adjusted (age, sex, and EPIC centre) mean levels of systolic blood pressure (SBP) and non-high-density lipoprotein cholesterol (non-HDL-C, estimated as total cholesterol minus HDL-C), by fifths of intake of each food, in participants with measurements available for blood pressure (*n* = 293 092) or lipids (*n* = 16 467). In addition, we included a model examining risks and further adjusting for SBP in participants with blood pressure data, and the potential explanatory role of SBP was estimated as (β_food_ – β_food+SBP_)/β_food_ × 100, with 95% confidence intervals estimated using a bootstrap method with 1000 resamplings.^28^ ^,^ ^29^

To examine whether the overall results might be influenced by reverse causality, as sensitivity analyses we repeated the analyses for ischaemic and haemorrhagic strokes after excluding the first 4 years of follow-up. To assess the relationship of the exposure at baseline with the risk for suffering stroke within a relatively short follow-up, closer to the time of dietary assessment, we also conducted analyses restricted to the first 10 years of follow-up. In addition, we examined the results stratified by age at recruitment (<55, 55–64, ≥65 years), sex, body mass index (BMI) (<25, 25–29.9, ≥30 kg/m^2^), history of diseases (no disease history, or with a history of diabetes, hypertension, and/or hyperlipidaemia); smoking status (never, former, or current smokers), European region (Northern, Central/Southern), and extent of stroke validation (partial, complete). Tests for heterogeneity of trend between subgroups were obtained by comparing the risk coefficients for each subgroup using inverse variance weighting, testing for statistical significance with a χ^2^ test on *k* − 1 df, where *k* is the number of subgroups.

All analyses were performed using Stata version 15.1 (Stata Corp, College Station, TX, USA), and a *P*-value of <0.05 was considered statistically significant.

## Results

### Baseline characteristics

Participant characteristics in the overall cohort and in participants who developed any type of stroke are shown in *Table 1* subdivided by sex. On average, compared with the overall cohort, men who developed stroke were 7 years older, and women who developed stroke were 10 years older at recruitment. Participants with stroke also had slightly higher BMI, and higher mean alcohol intake among drinkers, but were less likely to be current drinkers overall. They were also more likely to report heavy current smoking, less likely to report having vocational or university education, were generally less active, more likely to be unemployed, more likely to have a history of diabetes or hypertension, and were in a higher proportion from Northern Europe (Denmark, Norway, Sweden). Intakes of foods and fibre of participants are shown in Supplementary material online, *Table S3*. On average, participants who developed stroke reported lower intakes of cheese, cereals and cereal products, fruit and vegetables, legumes, nuts and seeds, but higher intakes of red and processed meat, and milk. Regional differences in food intakes were also observed, and are shown in Supplementary material online, *Table S4*. For example, higher fruit and vegetable intake was generally reported in Southern European countries (Greece, Italy, Spain), highest meat intake was reported in Denmark and highest yogurt intake in Sweden. Baseline characteristics by country are shown in Supplementary material online, *Table S5*.

**Table 1:** Participant characteristics at recruitment by sex and incident case status for any stroke in the EPIC study

Characteristic	Men		Women	
	All men	Men who developed stroke	All women	Women who developed stroke
Number of participants (%)	140 117 (33.5)	3635 (0.9)	278 212 (66.5)	3743 (0.9)
Age, year (SD)	52.0 (10.1)	59.1 (7.8)	50.4 (10.4)	60.0 (9.2)
Body mass index, kg/m^2^ (SD)	26.6 (3.7)	27.1 (3.8)	25.6 (4.5)	26.5 (4.7)
Alcohol in current drinkers, g/day (SD)	21.3 (23.1)	22.6 (25.2)	8.0 (11.0)	8.6 (12.3)
Not current alcohol drinker, *n* (%)	6288 (4.5)	176 (4.8)	30 487 (11.0)	483 (12.9)
Smoking status and cigarettes/day, *n* (%)				
Never smoker	46 878 (33.9)	985 (27.4)	146 424 (53.4)	1845 (49.6)
Former smoker	50 158 (36.3)	1303 (36.2)	65 583 (23.9)	795 (21.4)
Current smoker, <10 or number unknown	14 184 (10.3)	405 (11.3)	17 469 (6.4)	250 (6.7)
Current smoker, 10–19	10 435 (7.6)	368 (10.2)	26 359 (9.6)	492 (13.2)
Current smoker, ≥20	16 493 (11.9)	538 (14.9)	18 181 (6.6)	337 (9.1)
Highest level of education completed, *n* (%)				
None or primary	46 562 (34.2)	1683 (47.7)	90 273 (33.8)	1680 (48.1)
Secondary	18 468 (13.5)	346 (9.8)	47 535 (17.8)	384 (11.0)
Vocational or university	71 312 (52.3)	1496 (42.4)	129 338 (48.4)	1429 (40.9)
Cambridge physical activity index, *n* (%)				
Inactive	25 898 (18.9)	921 (25.8)	62 710 (23.0)	1257 (34.1)
Moderately inactive	42 910 (31.3)	1102 (30.8)	90 308 (33.2)	1238 (33.5)
Moderately active	34 218 (25.0)	793 (22.2)	72 553 (26.7)	684 (18.5)
Active	34 028 (24.8)	759 (21.2)	46 585 (17.1)	512 (13.9)
Employed or student, *n* (%)	94 176 (75.9)	1913 (59.0)	160 758 (64.8)	1440 (42.5)
History of diabetes, *n* (%)	4648 (3.4)	278 (8.0)	6038 (2.3)	252 (7.1)
Previous hypertension, *n* (%)	26 946 (20.1)	1106 (32.2)	48 910 (18.5)	1345 (37.3)
Previous hyperlipidaemia, *n* (%)	23 569 (20.2)	505 (21.0)	27 938 (13.6)	466 (18.5)
Region, *n* (%)^a^				
Northern Europe	46 925 (33.5)	2185 (60.1)	93 896 (33.7)	1814 (48.5)
Central Europe	53 642 (38.3)	789 (21.7)	112 631 (40.5)	1297 (34.7)
Southern Europe	39 550 (28.2)	661 (18.2)	71 685 (25.8)	632 (16.9)

Values are means (SD) or number (%), as indicated on the row.

a Northern Europe: Denmark, Norway, and Sweden; Central Europe: Germany, Netherlands, and UK; Southern Europe: Greece, Italy, and Spain.

### Risk of ischaemic and haemorrhagic stroke

**Figure 1:**
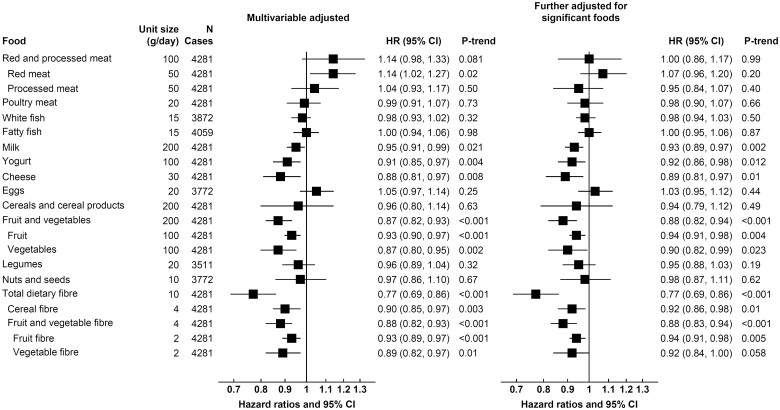
Hazard ratios (95% confidence intervals) for *ischaemic stroke* per unit higher calibrated intake of major foods and fibre in the EPIC study. Unit sizes represent approximate differences in mean 24 h recall intake between participants in the lowest and highest fifths of observed intake. Hazard ratios were adjusted for age, smoking status and number of cigarettes per day, history of diabetes, prior hypertension, prior hyperlipidaemia, Cambridge physical activity index, employment status, level of education completed, current alcohol consumption, body mass index, and calibrated intake of energy, and stratified by sex and EPIC centre. The right-hand column included further adjustment for significant foods from the left-hand column, details on the food adjustment and categorization of covariates can be found in the Supplementary material online, *Methods*.

After a mean follow-up of 12.7 years, there were 4281 incident cases of fatal or non-fatal ischaemic stroke, 1430 cases of haemorrhagic stroke, and 7378 cases of total stroke (ischaemic, haemorrhagic, and unspecified combined). The results of the associations between major foods and stroke were directionally similar for observed (fifths and per unit difference, Supplementary material online, *Tables S6–S8*) and calibrated intakes (per unit difference, *Figures 1 *and* 2*, Supplementary material online, *Figure S1*), and therefore, only the calibrated results are reported below. The ranges and medians of the fifths of intake, and per unit differences for each food, are shown in Supplementary material online, *Table S9*.

**Figure 2:**
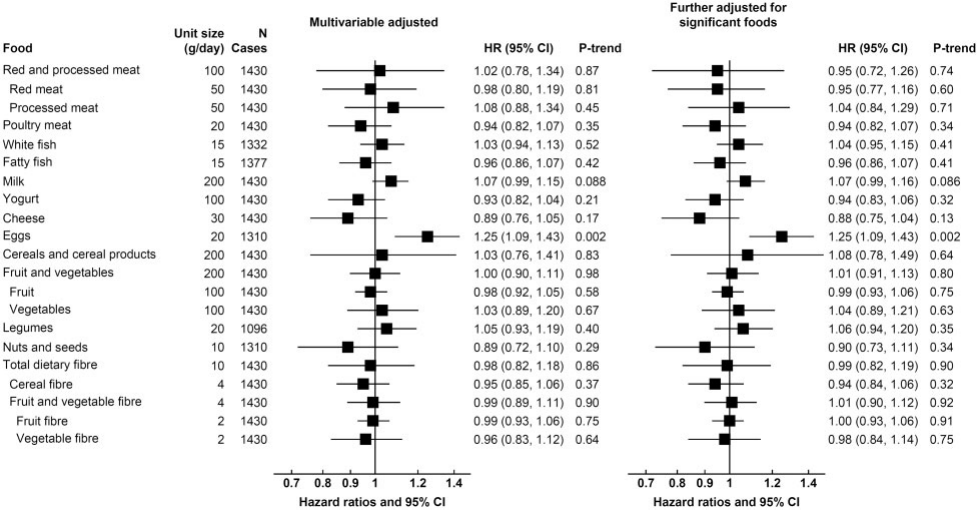
Hazard ratios (95% confidence intervals) for *haemorrhagic stroke* per unit higher calibrated intake of major foods and fibre in the EPIC study. Unit sizes represent approximate differences in mean 24 h recall intake between participants in the lowest and highest fifths of observed intake. Hazard ratios were adjusted for age, smoking status and number of cigarettes per day, history of diabetes, prior hypertension, prior hyperlipidaemia, Cambridge physical activity index, employment status, level of education completed, current alcohol consumption, body mass index, and calibrated intake of energy, and stratified by sex and EPIC centre. The right-hand column included further adjustment for eggs, with exception of the association of eggs which is based on the multivariable-adjusted model. Details on the categorization of covariates can be found in the Supplementary material online, *Methods*.

**Take home figure:**
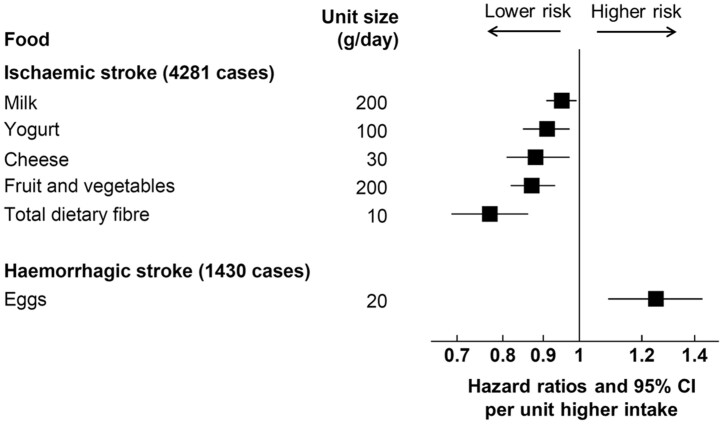
This is the largest study on multiple dietary factors and subtypes of stroke. Ischaemic and haemorrhagic stroke have markedly different patterns of dietary associations. For ischaemic stroke, lower risks were associated with higher consumption of dietary fibre, fruit and vegetables, and dairy foods. For haemorrhagic stroke, higher risk was associated with higher egg consumption. The results highlight the importance of examining stroke subtypes separately.

For ischaemic stroke (*Figure 1*), 200 g/day higher consumption of fruit and vegetables (as a combined group) and 10 g/day higher consumption total dietary fibre were associated with 13% (0.87, 0.82–0.93) and 23% (0.77, 0.69–0.86) lower risk, respectively (*P*-trend < 0.001 for both), which was equivalent to a rate difference of 1.02 fewer events (95% CI −1.48, −0.56) for fruit and vegetables, and 1.86 fewer events (−2.56, −1.16) for total dietary fibre, per 1000 participants in 10 years (*Table 2*). The results were similar when examining fruit and vegetables separately, and for subtypes of dietary fibre (cereal fibre, fruit and vegetable fibre). Further examination of fruit and vegetable subtypes showed inverse associations of risk with citrus fruits, hard fruits (apples and pears), bananas, fruiting vegetables, and root vegetables, but not leafy vegetables and cabbages (Supplementary material online, *Table S10*). Lower risk of ischaemic stroke was also observed with higher consumption of dairy foods, including milk (per 200 g/day; 0.95, 0.91–0.99, *P*-trend = 0.02), yogurt (per 100 g/day; 0.91, 0.85–0.97, *P*-trend = 0.004), and cheese (per 30 g/day; 0.88, 0.81–0.97, *P*-trend = 0.008), with rate differences of −0.42 (−0.78, −0.06); −0.73 (−1.22, −0.24); and −0.94 (−1.60, −0.28) events, respectively. On the other hand, a higher risk was observed with higher red meat consumption (per 50 g/day; 1.14, 1.02–1.27, *P*-trend = 0.02), but this attenuated when mutually adjusted for the other significant foods (1.07, 0.96–1.20, *P*-trend = 0.20, *Figure 1*) or for fibre intake alone (Supplementary material online, *Table S11*), and no significant association was observed for red and processed meat in the model mutually adjusted for all food groups (Supplementary material online, *Table S12*). In contrast, the associations for dietary fibre, fruit and vegetables, milk, yogurt and cheese remained similar in all models.

**Table 2:** Absolute rate differences (95% confidence intervals) for ischaemic and haemorrhagic risk per unit higher calibrated intake of selected major foods and fibre in the EPIC study

Outcome/food	Unit sizes (g/day)^a^	Number of additional events (95% CI) per unit higher intake, per 1000 participants over 10 years^b^
Ischaemic stroke (Average incidence = 8.04 cases per 1000 participants over 10 years)		
Red meat	50	1.10 (0.04, 2.17)
Milk	200	−0.42 (−0.78, −0.06)
Yogurt	100	−0.73 (−1.22, −0.24)
Cheese	30	−0.94 (−1.60, −0.28)
Fruit and vegetables	200	−1.02 (−1.48, −0.56)
Fruit	100	−0.55 (−0.86, −0.25)
Vegetables	100	−1.04 (−1.67, −0.42)
Total dietary fibre	10	−1.86 (−2.56, −1.16)
Cereal fibre	4	−0.77 (−1.28, −0.27)
Fruit and vegetable fibre	4	−1.00 (−1.49, −0.52)
Fruit fibre	2	−0.56 (−0.88, −0.25)
Vegetable fibre	2	−0.12 (−0.23, 0.002)
Haemorrhagic stroke (Average incidence = 2.69 cases per 1000 participants over 10 years)		
Eggs	20	0.66 (0.20, 1.11)

Foods or fibre were included on the basis of significant associations with ischaemic or haemorrhagic stroke risk in the multivariable adjusted hazard ratio analyses (*Figure 1* and *Figure 2*).

a Unit sizes represent approximate differences in mean 24 h recall intake between participants in the lowest and highest fifths of observed intake.

b Relative to the average incidence of ischaemic or haemorrhagic stroke in the EPIC study, based on a model adjusted for age, smoking status and number of cigarettes per day, history of diabetes, prior hypertension, prior hyperlipidaemia, Cambridge physical activity index, employment status, level of education completed, current alcohol consumption, body mass index, and calibrated intake of energy, and stratified by sex and EPIC centre. Details on the categorization of covariates can be found in the Supplementary material online, *Methods*.

For haemorrhagic stroke, higher egg consumption was associated with higher risk (per 20 g/day; 1.25, 1.09–1.43, *P*-trend = 0.002), equivalent to a rate difference of 0.66 (0.20, 1.11) more cases per 1000 participants in 10 years, but no other significant associations were observed (*Figure 2*, *Table 2*), and results were similar for the subarachnoid and intracerebral haemorrhage subtypes (Supplementary material online, *Table S13*). For total stroke (Supplementary material online, *Figure S1*), risks were similar to those for ischaemic stroke; we observed inverse associations for fruit and vegetables (per 200 g/day; 0.89, 0.85–0.93, *P*-trend < 0.001), dietary fibre (per 10 g/day; 0.80, 0.74–0.86, *P*-trend < 0.001), yogurt (per 100 g/day; 0.91, 0.87–0.96, *P*-trend, 0.001), cheese (per 30 g/day; 0.88, 0.82–0.94, *P* < 0.001), and a positive association for red and processed meat combined (per 100 g/day; 1.18, 1.05–1.33, *P*-trend = 0.005). A modest positive association was also observed for egg consumption (per 20 g/day; 1.07, 1.01–1.14, *P*-trend = 0.031) and risk of total stroke, similar to that for haemorrhagic stroke.

### Blood pressure and blood cholesterol concentrations

When we examined average SBP (Supplementary material online, *Table S14*) and non-HDL-C (Supplementary material online, *Table S15*) by fifths of observed intake in participants with these measures available, we observed that, in general, foods that were associated with higher risk of ischaemic stroke (red meat) were associated with both higher SBP and non-HDL-C, while foods and nutrients that were associated with lower risk (yogurt, cheese, fruit and vegetables, dietary fibre) were associated with either lower SBP or lower non-HDL-C, or both. Consumption of eggs, which was associated with higher risk of haemorrhagic stroke, was associated with marginally higher blood pressure, and lower non-HDL-C. However, in analyses of risks for both ischaemic and haemorrhagic stroke further adjusting for SBP, all associations were similar (Supplementary material online, *Tables S16* and *S17*). The addition of SBP to the model showed that the variable statistically explained only a small proportion of the association between fruit and vegetables (6.80%, 2.88–10.7 combined), total dietary fibre (6.40%, 2.26–10.5), and fruit and vegetable fibre (5.16%, 1.82–8.51) with ischaemic stroke risk, with no statistically significant attenuation for the other associations.

### Sensitivity analyses

When we excluded the first 4 years of follow-up similar associations were observed, except that higher milk intake was associated with a marginally higher risk of haemorrhagic stroke (Supplementary material online, *Table S18*). Results were also similar when we restricted the analyses to the first 10 years of follow-up; the positive association of red meat and inverse associations of milk and cheese with ischaemic stroke were no longer statistically significant, but the point estimates were essentially unchanged (Supplementary material online, *Table S19*). In analyses stratified by several covariates, there was limited evidence of between-subgroup heterogeneity (Supplementary material online, *Results*, *Tables S20–S33*).

## Discussion

In this large European cohort, lower risk of ischaemic stroke was observed with higher consumption of fruit and vegetables, dietary fibre and dairy products, while higher risk was observed with higher consumption of red meat. For haemorrhagic stroke, higher risk was associated with higher egg consumption. The different dietary factors associated with the two main types of stroke suggest differences in the aetiology of the stroke subtypes,^4^ ^,^ ^5^ highlighting the importance of assessing the subtypes separately. For total stroke, the results were reflective of those for ischaemic stroke, likely due to the higher proportion of cases with this outcome. The current findings support European dietary guidelines of consuming more fruit, vegetables, and other plant foods which contributes to the intake of dietary fibre.^30^ ^,^ ^31^

### Meat and meat products

Previous meta-analyses have reported a positive association between red and/or processed meat consumption and risk of total and ischaemic stroke, and no significant association with haemorrhagic stroke.^12^ Meat is a major source of saturated fat,^32^ which could contribute to atherosclerosis and thus higher risks of ischaemic stroke.^33^ ^,^ ^34^ However, the strength of the association for red meat in our cohort was relatively modest, and the association attenuated when adjusted for the other significant foods or for fibre alone, which suggests that the positive association observed might be partly due to an inverse association with the consumption of these other foods. For poultry, existing data are comparatively limited, but a meta-analysis reported no significant association for total stroke,^35^ which is consistent with our results. A recent analysis of the EPIC-Oxford cohort (a subset of EPIC) showed that compared with all meat-eaters, vegetarians had a higher risk of haemorrhagic stroke^36^; but the absence of an association of meat with risk for haemorrhagic stroke in the current analysis does not contradict this finding, because the vegetarians in EPIC-Oxford are a small proportion of the entire EPIC cohort.

### Fish and fish products

In contrast to our null associations, previous meta-analyses have reported a modest inverse association between fish consumption and both ischaemic and haemorrhagic stroke.^37^ ^,^ ^38^ However, the meta-analyses showed substantial heterogeneity of risk between studies, including heterogeneity by fish type^39–41^ or study location,^38^ possibly related to differences in cooking methods or presence of contaminants, which might result in inconsistency of findings.^42^ ^,^ ^43^

### Dairy products

Similar to our findings, several meta-analyses have reported inverse associations between total or individual dairy products with risk of stroke.^44^ ^,^ ^45^ Although dairy products are a substantial source of saturated fats in Europe, higher dairy consumption was in fact associated with slightly lower non-HDL-C concentrations in our sub-cohort. It has been hypothesized that the high calcium and potassium content of dairy products might have a role in stroke prevention.^46–48^ It was not possible to differentiate low-fat and high-fat dairy products in our study, and hence, we could not assess whether the associations might differ by their fat content. Evidence from both observational studies and randomized trials also suggests that dairy products, milk tripeptides, or milk protein supplements might have a role in lowering blood pressure.^49–51^ In the current study, high yogurt consumption was associated with lower SBP, although SBP was similar across categories of milk and cheese consumption, and the risks for all dairy products were similar after additional adjustment for SBP.

### Eggs

Previous evidence on egg consumption and stroke has been inconsistent.^6^ ^,^ ^52^ ^,^ ^53^ Egg consumption in EPIC-Europe was low overall (<20 g per day, compared with an average large egg of 60 g), but higher egg consumption in the current study was associated with slightly higher SBP, an established risk factor for both ischaemic and haemorrhagic strokes,^54^ and slightly lower non-HDL-C concentrations, which may be driven by residual confounding of factors not measured in this study.

### Cereals and cereal products

A recent meta-analysis reported that total cereal consumption was not associated with the risk of total stroke, but that whole-grain intake was inversely associated with the risk of ischaemic stroke.^55^ Because it was not possible to differentiate whole grain and refined grain intake in our study, the results were not directly comparable. However, an inverse association of whole-grain intake with ischaemic stroke is plausible if the association was mainly due to the fibre content in whole-grain cereals,^56^ which is much lower in refined cereal products,^20^ and would be consistent with our findings of an inverse association between cereal fibre and ischaemic stroke.

### Fruit and vegetables

Existing prospective evidence generally shows an inverse association between fruit and vegetable consumption and risk of total and ischaemic stroke.^6^ ^,^ ^11^ ^,^ ^15^ ^,^ ^20^ Given the strong inverse association of fibre with total and ischaemic stroke observed in the current study and in previous studies, as described below, the association between fruit and vegetables and stroke risk might be partly driven by their fibre content. It has also been suggested that fruit and vegetables could be beneficial because they provide various micronutrients including potassium and folate.^57^ Potassium supplements have been shown to reduce blood pressure in randomized trials,^58–60^ while higher folate intakes may lower plasma homocysteine concentrations, which is also positively associated with stroke risk.^61^ ^,^ ^62^ Fruit and vegetables are also a major source of dietary nitrates, which contributes to endogenous nitric oxide formation,^63^ ^,^ ^64^ and some evidence suggests that they have blood pressure lowering and vasoprotective properties.^65^ ^,^ ^66^

### Legumes

Published studies on legumes and stroke have found little evidence of an association with either total stroke or stroke subtypes,^67–69^ which is consistent with our results. Although legumes have been associated with several favourable effects on blood pressure and glycaemia, which might translate to favourable cardiovascular effects,^70^ it is possible that legume consumption in EPIC was too low for any possible effects to be apparent. Indeed, SBP and non-HDL-C were similar across fifths of legumes intake.

### Nuts and seeds

We did not find a significant association between nut consumption and either ischaemic or haemorrhagic stroke risk, and previous evidence has been inconsistent.^6^ ^,^ ^71^ ^,^ ^72^ Nuts are rich in unsaturated fats and high consumption has been linked to a lower total-to-HDL blood cholesterol ratio,^73^ which would be suggestive of cardiovascular benefits.^74^ However, nut consumption in EPIC was very low (median of <1 g/day overall, and 10 g/day in the top fifth), and therefore, it is possible that there is insufficient variation for any association with risk to be observed.

### Dietary fibre

Consistent with our results, previous evidence has shown an inverse association between dietary fibre and risk of total or ischaemic stroke, but no conclusive evidence on haemorrhagic stroke.^16–19^ ^,^ ^75^ Our study also showed similar results across subtypes of dietary fibre, for which there was previously insufficient evidence.^76^ The inverse association for fibre might be plausible due to several mechanisms. Although differences in SBP comparing participants who consume high vs. low levels of fibre were only modest and results were similar with and without adjustment for SBP, with this variable only explaining a small proportion of the association, a meta-analyses of randomized trials of fibre supplementation showed a significant lowering of diastolic blood pressure.^77^ Fibre has also been found to reduce total and low-density lipoprotein cholesterol levels in trials,^77^ and in the current study it was associated with lower non-HDL-C.

### Strengths and limitations

Strengths of this study include its large sample size across nine European countries, prospective design and a long follow-up. We included major foods groups in our study, calibrated the dietary data using 24 h recalls, and adjusted for multiple confounders. One of the limitations is that diet was only collected at one time point. Furthermore, because a large number of associations were tested in this study, false-positive findings are possible; e.g. although the strong inverse associations for fruit and vegetables and dietary fibre would have remained significant even when considering a Bonferroni adjusted *P*-value threshold of 0.0024, some other associations, such as for red meat and dairy with ischaemic stroke, would no longer be considered statistically significant. However, this approach involves the use of an arbitrary *P*-value which is dependent on the number of comparisons tested, and thus the results might be better appraised in regards to the strengths and confidence intervals of the associations, rather than solely on the basis of statistical significance.^78^ Due to the multi-centre design of the cohort, there were some variations in the ascertainment and validation of the endpoint, but results were similar when stratified by extent of stroke validation. The study is observational, and as with all such studies, causality cannot be determined and residual confounding cannot be ruled out. Although competing risk from other causes of death is possible, it is unlikely to be significant since the great majority of participants (88%) were alive at the end of follow-up. The study included predominantly white European individuals, and as with all long-term prospective studies, it is not clear whether dietary intakes at recruitment to the study are representative of the contemporary European population, and hence the generalizability of the findings might be limited. Information on medication use (including statins) was not available, and therefore, their possible influences could not be assessed. Reverse causality is possible, although results were similar after excluding the first 4 years of follow-up. Information on possible mediators (blood pressure and cholesterol) was only available at study baseline in a subset of the cohort, and the estimates of their effects could, therefore, be prone to regression dilution bias.

## Conclusions

In this large European cohort, we observed inverse associations of consumption of fruit and vegetables, dietary fibre, and dairy products, with risk of total and ischaemic stroke; a modest positive association of red or processed meat consumption with risk of total and ischaemic stroke; and a positive association of egg consumption with risk of total and haemorrhagic stroke. The observed associations might be partly explained by effects on blood pressure and blood cholesterol. The different dietary factors associated with risk of ischaemic and haemorrhagic stroke highlight the importance of differentiating stroke subtypes in epidemiological studies.

## Supplementary Material

EURHEARTJ_41_28_2632_s5
