# Supplementary material for: The associations of major foods and fibre with risks of ischaemic and haemorrhagic stroke: a prospective study of 418 329 participants in the EPIC cohort across nine European countries
Source: Eur Heart J. 2020 Feb 24;41(28):2632–40. doi: 10.1093/eurheartj/ehaa007 (PMC7377582; doi:10.1093/eurheartj/ehaa007)
Supplement: EURHEARTJ_41_28_2632_s5 [file eurheartj_41_28_2632_s5.docx]

**Supplementary materials**

[**Supplementary methods:** 4](#_Toc23340415)

[**Supplementary results:** 6](#_Toc23340416)

[**Supplementary table 1.** Examples of foods items included and data availability for each food group in the EPIC study^1^ 7](#_Toc23340417)

[**Supplementary table 2**. Follow-up duration and number of cases in each EPIC country. 8](#_Toc23340418)

[**Supplementary table 3.** Dietary intakes of participants at recruitment by sex and incident case status for any stroke in the EPIC study. 9](#_Toc23340419)

[**Supplementary table 4**. Observed intakes of foods and fibre in each EPIC country. 10](#_Toc23340420)

[**Supplementary table 5**. Covariate characteristics in each EPIC country. 11](#_Toc23340421)

[**Supplementary table 6**. Hazard ratios^1^ (95% confidence intervals) for **ischaemic stroke** by overall fifths (relative to the bottom fifth of intake) and per unit higher observed intake of major foods and fibre in the EPIC study. 12](#_Toc23340422)

[**Supplementary table 7**. Hazard ratios^1^ (95% confidence intervals) for **haemorrhagic stroke** by overall fifths (relative to the bottom fifth of intake) and per unit higher observed intake of major foods and fibre in the EPIC study. 13](#_Toc23340423)

[**Supplementary table 8**. Hazard ratios^1^ (95% confidence intervals) for **total stroke** by overall fifths (relative to the bottom fifth of intake) and per unit higher observed intake of major foods and fibre in the EPIC study. 14](#_Toc23340424)

[**Supplementary figure 1:** Hazard ratios (95% confidence intervals) for **total stroke** per unit higher calibrated intake of major foods and fibre in the EPIC study. 15](#_Toc23340425)

[**Supplementary table 9**. Ranges of intake by fifths of observed intake and unit sizes of selected major foods and fibre in the EPIC study. 16](#_Toc23340426)

[**Supplementary table 10**. Hazard ratios (95% confidence intervals) for stroke per unit higher^1^ calibrated intake of **subtypes of fruit and vegetables** in the EPIC study. 17](#_Toc23340427)

[**Supplementary table 11**. Hazard ratios (95% confidence intervals) for stroke per unit higher^1^ calibrated intake of major foods, **adjusted for fibre**^2^, in the EPIC study. 18](#_Toc23340428)

[**Supplementary table 12**. Hazard ratios^1^ (95% confidence intervals) for stroke, **with mutual adjustment of all major foods**, in the EPIC study. 19](#_Toc23340429)

[**Supplementary table 13**. Hazard ratios (95% confidence intervals) for **subarachnoid haemorrhage** and **intracerebral haemorrhage** per unit higher^1^ calibrated intake of major foods and fibre in the EPIC study. 20](#_Toc23340430)

[**Supplementary table 14**. **Adjusted mean systolic blood pressure^1^** (mm Hg; 95% confidence intervals) by overall fifths of observed intake of major foods and fibre in up to 293,092 participants in the EPIC study. 21](#_Toc23340431)

[**Supplementary table 15**. **Adjusted mean non-high density lipoprotein cholesterol^1^** (mmol/l; 95% confidence intervals) by overall fifths of observed intake of major foods and fibre in up to 16467 participants in the EPIC study. 22](#_Toc23340432)

[**Supplementary table 16**. Hazard ratios (95% confidence intervals) for **ischaemic stroke** per unit higher^1^ calibrated intake of major foods and fibre, with **additional adjustment for systolic blood pressure**, in up to 293,092 participants in the EPIC study. 23](#_Toc23340433)

[**Supplementary table 17**. Hazard ratios (95% confidence intervals) for **haemorrhagic stroke** per unit higher^1^ calibrated intake of major foods and fibre, with **additional adjustment for systolic blood pressure**, in up to 293,092 participants in the EPIC study. 24](#_Toc23340434)

[**Supplementary table 18**. Hazard ratios (95% confidence intervals) for stroke per unit higher^1^ calibrated intake of major foods and fibre, after **excluding the first 4 years of follow up** in the EPIC study. 25](#_Toc23340435)

[**Supplementary table 19**. Hazard ratios (95% confidence intervals) for stroke per unit higher^1^ calibrated intake of major foods and fibre, **restricting to the first 10 years of follow up** in the EPIC study. 26](#_Toc23340436)

[**Supplementary table 20**: Hazard ratios (95% confidence intervals) for **ischaemic stroke** per unit higher^1^ calibrated intake of major foods and fibre, **stratified** **by age at recruitment**, showing results from independent subset analyses in the EPIC study. 27](#_Toc23340437)

[**Supplementary table 21**: Hazard ratios (95% confidence intervals) for **haemorrhagic stroke** per unit higher^1^ calibrated intake of major foods and fibre, **stratified** **by age at recruitment**, showing results from independent subset analyses in the EPIC study. 28](#_Toc23340438)

[**Supplementary table 22**: Hazard ratios (95% confidence intervals) for **ischaemic stroke** per unit higher^1^ calibrated intake of major foods and fibre, **stratified by sex**, showing results from independent subset analyses in the EPIC study. 29](#_Toc23340439)

[**Supplementary table 23**: Hazard ratios (95% confidence intervals) for **haemorrhagic stroke** per unit higher^1^ calibrated intake of major foods and fibre, **stratified** **by sex**, showing results from independent subset analyses in the EPIC study. 30](#_Toc23340440)

[**Supplementary table 24**: Hazard ratios (95% confidence intervals) for **ischaemic stroke** per unit higher^1^ calibrated intake of major foods and fibre, **stratified by body mass index**, showing results from independent subset analyses in the EPIC study. 31](#_Toc23340441)

[**Supplementary table 25**: Hazard ratios (95% confidence intervals) for **haemorrhagic stroke** per unit higher^1^ calibrated intake of major foods and fibre, **stratified** **by body mass index**, showing results from independent subset analyses in the EPIC study. 32](#_Toc23340442)

[**Supplementary table 26**: Hazard ratios (95% confidence intervals) for **ischaemic stroke** per unit higher^1^ calibrated intake of major foods and fibre, **stratified** **by prior disease status**, showing results from independent subset analyses in the EPIC study. 33](#_Toc23340443)

[**Supplementary table 27**: Hazard ratios (95% confidence intervals) for **haemorrhagic stroke** per unit higher^1^ calibrated intake of major foods and fibre, **stratified by prior disease status**, showing results from independent subset analyses in the EPIC study. 34](#_Toc23340444)

[**Supplementary table 28**: Hazard ratios (95% confidence intervals) for **ischaemic stroke** per unit higher^1^ calibrated intake of major foods and fibre, **stratified by smoking status**, showing results from independent subset analyses in the EPIC study. 35](#_Toc23340445)

[**Supplementary table 29**: Hazard ratios (95% confidence intervals) for **haemorrhagic stroke** per unit higher^1^ calibrated intake of major foods and fibre, **stratified** **by smoking status**, showing results from independent subset analyses in the EPIC study. 36](#_Toc23340446)

[**Supplementary table 30**: Hazard ratios (95% confidence intervals) for **ischaemic stroke** per unit higher^1^ of calibrated intake of selected animal foods, plant foods, and fibre, **stratified by European region**, showing results from independent subset analyses in the EPIC study. 37](#_Toc23340447)

[**Supplementary table 31**: Hazard ratios (95% confidence intervals) for **haemorrhagic stroke** per unit higher^1^ calibrated intake of major foods and fibre, **stratified by European region**, showing results from independent subset analyses in the EPIC study. 38](#_Toc23340448)

[**Supplementary table 32**: Hazard ratios (95% confidence intervals) for **ischaemic stroke** per unit higher^1^ calibrated intake of major foods and fibre, **stratified by** **extent of stroke validation**, showing results from independent subset analyses in the EPIC study. 39](#_Toc23340449)

[**Supplementary table 33**: Hazard ratios (95% confidence intervals) for **haemorrhagic stroke** per unit higher^1^ calibrated intake of major foods and fibre, **stratified by** **extent of stroke validation**, showing results from independent subset analyses in the EPIC study. 40](#_Toc23340450)

[**References** 41](#_Toc23340451)

**Supplementary methods:**

*Exclusion criteria*

The EPIC study recruited approximately 520,000 participants from 23 centres across 10 European countries (Denmark, France, Germany, Greece, Italy, the Netherlands, Norway, Spain, Sweden, and the United Kingdom (UK)) overall. Participants were excluded if they had no lifestyle data, no dietary data, or were in the top or bottom 1% on the ratio of energy intake to energy requirement (n=16,837). France was excluded because they did not provide stroke incidence data (n=72,987). Participants were also excluded if they had a known or possible history of myocardial infarction or stroke based on self-report or outcomes data, or had unknown prior disease status at recruitment (n=10,325), had an event after the end of follow-up date (n=15), or had no follow-up (n=9). After the exclusions, 418,329 participants were included in these analyses.

*Physical activity*

Physical activity was assessed using the validated EPIC Physical Activity Questionnaire (EPIC-PAQ) which asked about recreational, household, and occupational physical activity in the past year, in all EPIC centres except in Norway.^1–3^ Based on responses to these questions, a four-point index (Cambridge physical activity index) was derived as a simple way of measuring combined physical activity, and the details, interpretation, and validation of the method against objective measures has been previously reported.^4^ Total physical activity in Norway was measured using a scale that ranged from 1 to 10 and was then converted to the Cambridge index^3,5^.

*Anthropometry*

Weight and height was also measured in most centres, except for some participants in Norway and the UK, among whom it was self-reported.

*Blood pressure*

Blood pressure (systolic and diastolic) was measured at the time of recruitment for most participants by trained personnel (blood pressure was not available for participants from Asturias and Navarra in Spain, and Norway). Two readings were performed on the right arm in a sitting position (spaced by 1-5 minutes) after an initial resting time of at least 5 minutes by use of a standard mercury manometer or oscillometric device (with the exception of the Danish and Swedish centres where one single measurement was taken in the supine position).

*Blood cholesterol*

Lipid assays were done on stored plasma samples in a subset of participants.^6,7^ Total cholesterol was measured by enzymatic assay at Stichling Huisartsen Laboratorium (Etten_Leur, Netherlands), and an enzymatic colorimetric assay method (Roche) was used to measure HDL cholesterol directly. Lipid measurements were not available for any participants from Norway.

*Calibration of dietary data*

To improve the comparability of dietary data across participating centres and to correct for measurement error, dietary data from the 24 hour recalls, available in an 8% random sample of the cohort, were used to provide statistically calibrated estimates of dietary intakes, and HRs with 95% CIs were estimated per unit differences of observed and calibrated intakes of each food group or subset of fibre. The calibration was done by regressing the centre and sex-specific 24 hour recall data from the 8% random sample on the observed intakes from FFQs in the same people using a fixed-effects linear model, thereby generating a calibrated prediction of intake for each observed intake from FFQs in all participants.^8,9^

*Ascertainment of stroke cases*

Non-fatal incident cases were ascertained in each EPIC centre using a combination of record linkage to morbidity or hospital registries and self-reports, details on the methods were previously reported.^7^ For mortality data, information on vital status was collected from mortality registries at the regional or national level in most centres, except in Potsdam (Germany) and Greece where vital status was ascertained by active follow-up of study participants and next of kin. In Denmark, Germany, Greece, Italy and Spain, all suspected cases were validated, while in the Netherlands, Norway, Sweden and the UK, a subset was validated to assess the accuracy of the overall ascertainment process. Validation was done using a range of methods to confirm diagnosis, including retrieving and assessing medical records or hospital discharge notes, contact with medical professionals, retrieval and assessment of death certificates, or verbal autopsy with the next of kin.

*Unit sizes and categorisation of foods*

The sizes of the units were chosen to approximate the difference in mean 24 hour recall intake between participants in the lowest and highest fifths of observed intake. For examining differences by fifths of intakes, participants were divided into fifths of observed intake of each food based on their responses to the recruitment FFQ, calculated using the whole EPIC cohort. For any foods with more than 20% zero values (e.g. yogurt, nuts and seeds), the categories were approximate fifths. A trend test was performed by replacing the categorical fifth of intake by the mean observed intake in that fifth as a continuous variable.

*Categorisation of covariates*

All our analyses were adjusted for age (continuous), smoking status and number of cigarettes per day (never smoker, former smoker, current smoker <10 cigs/d, current smoker 10-19 cigs/d, current smoker 20+ cigs/d, unknown), self-reported history of diabetes, hypertension, or hyperlipidaemia (yes, no, unknown for each, based on responses to the question “Have you ever been told by a doctor that you have, or had [the condition]?”), Cambridge physical activity index (inactive, moderately inactive, moderately active, active, unknown, details in physical activity section above), employment status (employed or student, not employed or student, unknown), level of education completed (none or primary, secondary, vocational or university, unknown), current alcohol consumption (non-drinkers and sex-specific fifths of intake among drinkers), body mass index (BMI, <22.5, 22.5-24.9, 25.0-27.4, 27.5-29.9, ≥30.0 kg/m2, unknown), and calibrated or observed intake of energy (continuous), as appropriate, and stratified by sex and EPIC centre.

*Additional adjustment for significant foods*

For ischaemic stroke, the additional adjustment for significant foods included dietary fibre, fruit and vegetables, milk, yogurt, cheese, and red meat, as follows. For red and processed meat, and red meat, the model included total dietary fibre, fruit and vegetables, milk, yogurt, and cheese; for processed meat, poultry meat, white fish, fatty fish, milk, yogurt, cheese, and eggs, the model included total dietary fibre, fruit and vegetables, milk, yogurt, cheese, and red meat; for cereal and cereal products, and cereal fibre, the model included fruit and vegetables, milk, yogurt, cheese and red meat; for fruit and vegetables, and fruit and vegetable fibre, the model included cereal fibre, milk, yogurt, cheese, and red meat; for fruit, and fruit fibre, the model included cereal fibre, vegetables, milk, yogurt, cheese, and red meat; for vegetables, and vegetable fibre, the model included cereal fibre, fruit, milk, yogurt, cheese, and red meat; for legumes, and nuts and seeds, the model included cereal fibre, fruit and vegetables, milk, yogurt, cheese and red meat; for total dietary fibre, the model included milk, yogurt, cheese and red meat. For haemorrhagic stroke, the additional adjustment for significant foods included eggs in all analyses except for the analyses of eggs.

**Supplementary results:**

*Results from subgroup analyses*

To assess the extent to which our overall results varied across subgroups of participants, we examined our results stratified by age at recruitment, sex, BMI, history of diseases, smoking status, European region, and extent of stroke validation. Although we did not observe between-subgroup heterogeneity that would be statistically significant if we accounted for the large number of tests conducted, several associations were nominally significant (p-het<0.05). A significant inverse association between fruit and vegetable intake and ischaemic stroke was only observed in men (p-heterogeneity=0.04, *I^2^*=76%, Supplementary table 22), in former and current smokers (p-het=0.038, *I^2^*=70%, Supplementary table 28), and in participants from Northern Europe (p-het=0.016, *I^2^*=83%, Supplementary table 30); a significant inverse association between fruit and vegetable fibre and ischaemic stroke was also only observed in former and current smokers (p-het=0.027, *I^2^*=72%,) and participants from Northern Europe (p-het=0.02, *I^2^*=82%,). A significant inverse association of ischaemic stroke with cereal fibre was only observed in participants aged above 65 years (p-het=0.04, *I^2^*=68%, Supplementary table 20), and with cheese only in participants with no disease history of diabetes, previous hypertension, or previous hyperlipidaemia (p-het=0.041, *I^2^*=76%, Supplementary table 26). Future studies will be needed to replicate these hypothesis-generating subgroup-specific associations.

**Supplementary table 1.** Examples of foods items included and data availability for each food group in the EPIC study^1^

| Food | Food items included and data availability |
| --- | --- |
|  |  |
| Red and processed meat | Sum of unprocessed red meat and process meat below |
| Red meat | All fresh, frozen or minced unprocessed red meats, including beef, veal, pork, lamb, mutton, goat, horse, hamburger, meatballs, minced meat |
| Processed meat | Processed meat products, including poultry, preserved by methods other than freezing, such as salting with or without nitrites, smoking, marinating, air drying, or heating, and including ham, bacon, sausages, blood sausages, chicken sausage, meat cuts, liver paté, salami, bologna, tinned meat, luncheon meat, corned beef and black pudding |
| Poultry meat | All fresh, frozen, or minced chicken, turkey, duck, goose, rabbit, excluding processed poultry |
| White fish | Cod, haddock, plaice, and other lean or white fish (fat <4g/100g); data not available for the centres in Naples, Heidelberg, and Potsdam |
| Fatty fish | Salmon, tuna, trout, herring, kippers, and mackerel, and other fatty fish (fat ≥4g/100g); data not available for the centre in Potsdam |
| Milk | Plain milk (whole-fat, skimmed, semi-skimmed and not specified) including buttermilk, excluding milk-based beverages |
| Yogurt | Plain yogurt, fruit yogurt, kefir, fermented milk, thick milk, sour milk |
| Cheese | All fresh, fermented and matured cheese such as ricotta, cheddar, gouda, camembert, mozzarella |
| Eggs | Whole eggs and egg products, such as eggs consumed as components of recipes; data not available for the centre in Umeå |
| Cereals and cereal products | All bread, pasta, rice and other grains, plus cereal products such as pastry, crackers and breakfast cereals, but excluding cakes and biscuits |
| Fruit and vegetables | Sum of fruit and vegetables intake below |
| Fruit | All fruit including citrus fruits, apples and pears, grapes, stone fruits, berries, bananas, kiwis, and other citrus and non-citrus fruits |
| Vegetables | All vegetables including leafy vegetables, fruiting vegetables, root vegetables (excluding potatoes and other tubers), cabbages, mushrooms, grain and pod vegetables, allium vegetables, stalk vegetables, salads, and mixed vegetables |
| Legumes | Beans, chickpeas, split peas, lentils; data not available for the centres in Denmark and Norway |
| Nuts and seeds | All nuts and seeds including tree nuts, peanuts, seeds, and nuts and seeds products (e.g. spreads); Umeå was excluded in all analyses of nuts and seeds owing to the inclusion of potato chips in the same food group for this centre. |
| Total dietary fibre | Fibre from all foods including cereals, fruits and vegetables and other foods. Estimated using the Association of Official Agricultural Chemists gravimetric method in all countries except the UK and Greece, where the Englyst method was used |
| Cereal fibre | Estimated fibre content from bread, pasta and rice, and cereal products |
| Fruit and vegetable fibre | Estimated fibre content from fruit and vegetables, as described above |
| Fruit fibre | Estimated fibre content from fruit, as described above |
| Vegetable fibre | Estimated fibre content from vegetables, as described above |
| ^1^ Data available in all centres unless otherwise stated. | |

**Supplementary table 2**. Follow-up duration and number of cases in each EPIC country.

|  | Country | | | | | | | | | |
| --- | --- | --- | --- | --- | --- | --- | --- | --- | --- | --- |
|  | Italy | Spain | UK-general^1^ | UK-health conscious^1^ | The Netherlands | Greece | Germany | Sweden | Denmark | Norway |
| Total number | 45,301 | 40,114 | 30,869 | 47,617 | 37,266 | 25,820 | 50,521 | 51,306 | 53,322 | 36,193 |
| Recruitment period | 1992-1998 | 1992-1996 | 1993-1998 | 1994-2000 | 1993-1997 | 1993-1999 | 1994-1998 | 1991-1996 | 1993-1997 | 1998 |
| Last year of follow-up | 2008 | 2012 | 2009 | 2009 | 2008 | 2009 | 2008 | 2008 | 2009 | 2010 |
| Duration of follow-up, years, mean (SD) | 11.2 (2.4) | 14.8 (2.3) | 11.0 (2.0) | 12.6 (1.3) | 12.8 (1.6) | 13.0 (1.7) | 11.1 (1.7) | 14.2 (2.1) | 13.5 (1.6) | 12.1 (0.2) |
| Total stroke cases | 301 | 678 | 559 | 474 | 549 | 314 | 504 | 2166 | 1795 | 38 |
| Ischaemic stroke, n  (% total stroke) | 141 (46.8) | 457 (67.4) | 220 (39.4) | 171 (36.1) | 320 (58.3) | 18 (5.7) | 398 (79.0) | 1786 (82.5) | 763 (42.5) | 7 (18.4) |
| Haemorrhagic  stroke, n (% total  stroke) | 83 (27.6) | 129 (19.0) | 123 (22.0) | 135 (28.5) | 126 (23.0) | 32 (10.2) | 90 (17.9) | 378 (17.5) | 307 (17.1) | 27 (71.1) |
| Status at end of follow-up, n (%) |  |  |  |  |  |  |  |  |  |  |
| Alive | 42,068 (92.9) | 36,603 (91.3) | 24,066 (78.0) | 41,684 (87.5) | 32,798 (88.0) | 23,116 (89.5) | 46,364 (91.8) | 40,797 (79.5) | 44,298 (83.1) | 34,608 (95.6) |
| Dead | 2,456 (5.4) | 3,424 (8.5) | 6,694 (21.7) | 4,217 (8.9) | 3,563 (9.6) | 2,367 (9.2) | 3,538 (7.0) | 9,050 (17.6) | 8,676 (16.3) | 1,388 (3.8) |
| Censored for other  reasons^2^ | 777 (1.7) | 87 (0.2) | 109 (0.4) | 1716 (3.6) | 905 (2.4) | 337 (1.3) | 619 (1.2) | 1459 (2.8) | 348 (0.7) | 197 (0.5) |
| ^1^ United Kingdom-general includes participants recruited from general practices via both the Cambridge and Oxford centres. United Kingdom-health conscious includes participants recruited via postal methods, and includes a large proportion of vegetarians and health conscious people.  ^2^ Censored due to opt-out, moved to a different region or country, or loss to follow up for any other reason. | | | | | | | | | | |

**Supplementary table 3.** Dietary intakes of participants at recruitment by sex and incident case status for any stroke in the EPIC study.

| Dietary intakes, g/day | Men | |  | Women | |
| --- | --- | --- | --- | --- | --- |
|  | All men | Men who developed stroke |  | All women | Women who developed stroke |
| Red and processed meat | 90 (54, 131) | 98 (62, 138) |  | 57 (33, 85) | 62 (37, 91) |
| Red meat | 48 (23, 80) | 52 (25, 85) |  | 28 (13, 50) | 34 (16, 58) |
| Processed meat | 33 (15, 58) | 37 (20, 61) |  | 20 (8, 38) | 20 (10, 37) |
| Poultry meat | 15 (7, 28) | 14 (5, 26) |  | 13 (5, 22) | 11 (3, 21) |
| White fish^1^ | 11 (3, 22) | 13 (2, 23) |  | 11 (2, 24) | 10 (1, 19) |
| Fatty fish^2^ | 8 (1, 15) | 8 (2, 17) |  | 7 (1, 16) | 6 (1, 15) |
| Milk | 150 (26, 295) | 174 (32, 374) |  | 152 (43, 294) | 200 (52, 359) |
| Yogurt | 18 (1, 71) | 15 (0, 87) |  | 27 (3, 89) | 36 (3, 107) |
| Cheese | 28 (15, 52) | 24 (13, 50) |  | 27 (15, 48) | 23 (13, 43) |
| Eggs^3^ | 15 (7, 26) | 18 (8, 32) |  | 13 (7, 22) | 14 (7, 23) |
| Cereals and cereal products | 236 (171, 316) | 214 (153, 291) |  | 185 (135, 247) | 162 (117, 224) |
| Fruit and vegetables | 328 (203, 521) | 288 (179, 452) |  | 386 (254, 565) | 375 (249, 539) |
| Fruit | 156 (82, 280) | 135 (69, 240) |  | 201 (114, 319) | 199 (116, 309) |
| Vegetables | 151 (94, 248) | 137 (80, 221) |  | 166 (108, 259) | 158 (101, 241) |
| Legumes^4^ | 8 (2, 23) | 6 (0, 18) |  | 8 (1, 22) | 5 (0, 16) |
| Nuts and seeds^3^ | 0.8 (0.1, 4.3) | 0.7 (0.0, 2.3) |  | 0.8 (0.0, 3.5) | 0.5 (0.0, 2.3) |
| Total dietary fibre | 23 (18, 29) | 22 (17, 28) |  | 21 (17, 26) | 20 (16, 25) |
| Cereal fibre | 10 (6, 14) | 10 (6, 14) |  | 8 (5, 11) | 7 (5, 11) |
| Fruit and vegetable fibre | 7 (4, 10) | 6 (4, 9) |  | 8 (5, 11) | 8 (5, 11) |
| Fruit fibre | 3 (1, 5) | 3 (1, 4) |  | 4 (2, 6) | 4 (2, 6) |
| Vegetable fibre | 4 (2, 5) | 3 (2, 5) |  | 4 (3, 6) | 4 (2, 5) |
|  |  |  |  |  |  |
| Energy intake, kcal/day (SD) | 2416 (662) | 2388 (646) |  | 1876 (515) | 1867 (520) |
| Values are medians (lower and upper quartiles) except where otherwise indicated  ^1^ Unavailable for Naples, Heidelberg and Potsdam.  ^2^ Unavailable for Potsdam.  ^3^ Unavailable for Umea.  ^4^ Unavailable for Denmark and Norway. | | | | | |

**Supplementary table 4**. Observed intakes of foods and fibre in each EPIC country.

|  | Country | | | | | | | | | |
| --- | --- | --- | --- | --- | --- | --- | --- | --- | --- | --- |
| Foods, g/day, medians (lower and upper quartiles) | Italy | Spain | UK-general^1^ | UK-health conscious^1^ | The Netherlands | Greece | Germany | Sweden | Denmark | Norway |
| Red and processed meat | 68 (45, 96) | 70 (43, 106) | 62 (39, 92) | 7 (2, 39) | 88 (53, 120) | 49 (32, 71) | 81 (52, 118) | 59 (38, 89) | 96 (69, 133) | 58 (41, 79) |
| Red meat | 45 (28, 65) | 36 (17, 59) | 34 (24, 66) | 4 (1, 19) | 61 (34, 85) | 47 (30, 67) | 24 (14, 39) | 20 (10, 38) | 70 (50, 95) | 21 (13, 29) |
| Processed meat | 19 (11, 33) | 29 (14, 51) | 21 (13, 35) | 4 (0, 15) | 23 (11, 39) | 2 (0, 4) | 53 (31, 79) | 34 (21, 53) | 24 (14, 39) | 36 (23, 52) |
| Poultry meat | 18 (10, 29) | 27 (14, 44) | 16 (16, 49) | 1 (0, 16) | 9 (4, 16) | 19 (13, 37) | 8 (4, 16) | 7 (0, 15) | 17 (10, 27) | 16 (6, 16) |
| White fish^2^ | 6 (3, 13) | 24 (8, 47) | 18 (10, 26) | 10 (0, 19) | 1 (1, 3) | 12 (9, 19) | --- | 6 (0, 14) | 15 (10, 22) | 41 (23, 67) |
| Fatty fish^3^ | 8 (4, 15) | 10 (2, 23) | 8 (1, 16) | 8 (0, 16) | 2 (1, 4) | 3 (2, 6) | 1 (1, 6) | 4 (0, 11) | 11 (7, 18) | 18 (8, 31) |
| Milk | 120 (0, 189) | 200 (111,307) | 295 (293,440) | 293 (146,439) | 197 (60, 377) | 70 (12, 157) | 33 (2, 103) | 194 (76, 402) | 174 (30, 429) | 120 (54, 204) |
| Yogurt | 8 (0, 42) | 0 (0, 54) | 18 (0, 55) | 19 (10, 63) | 46 (13, 87) | 27 (13, 42) | 53 (13, 108) | 79 (16, 179) | 30 (5, 159) | 25 (0, 25) |
| Cheese | 57 (37, 85) | 14 (1, 39) | 15 (5, 19) | 17 (7, 28) | 31 (20, 49) | 54 (30, 83) | 31 (17, 40) | 27 (16, 47) | 26 (18, 53) | 27 (17, 51) |
| Eggs^4^ | 14 (8, 22) | 22 (12, 35) | 7 (5, 22) | 7 (4, 22) | 14 (7, 21) | 10 (6, 15) | 12 (6, 20) | 16 (8, 27) | 18 (11, 35) | 15 (8, 18) |
| Cereals and cereal products | 305 (217,416) | 195 (138,264) | 164 (111,235) | 221 (157,300) | 179 (133,241) | 197 (151,251) | 186 (138,239) | 183 (130,260) | 202 (153,260) | 195 (149,242) |
| Fruit and vegetables | 475 (346,632) | 528 (362,727) | 433 (311,582) | 498 (357,682) | 303 (217,415) | 775 (606,968) | 237 (175,323) | 269 (167,395) | 320 (213,459) | 268 (185,379) |
| Fruit | 303 (205,425) | 276 (160,430) | 190 (114,289) | 222 (135,343) | 170 (103,260) | 334 (234,447) | 106 (78, 187) | 147 (86, 241) | 144 (75, 239) | 130 (72, 213) |
| Vegetables | 156 (105,225) | 224 (143,329) | 230 (164,311) | 259 (186,353) | 124 (96,158) | 429 (332,545) | 113 (85, 150) | 104 (55, 167) | 163(105,232) | 127 (87, 179) |
| Legumes^5^ | 5 (2, 12) | 45 (25, 69) | 11 (6, 16) | 17 (11, 40) | 7 (2, 13) | 18 (11, 30) | 2 (1, 5) | 0 (0, 5) | --- | --- |
| Nuts and seeds^4^ | 0 (0, 1) | 0 (0, 4) | 0 (0, 3) | 3 (0, 10) | 4 (2, 11) | 3 (0, 5) | 1 (0, 3) | 0 (0, 1) | 1 (0, 2) | 0 (0, 3) |
| Total dietary fibre | 21 (17, 26) | 24 (19, 29) | 21 (17, 26) | 25 (19, 31) | 22 (19, 27) | 21 (17, 25) | 21 (17, 26) | 19 (15, 24) | 24 (19, 30) | 20 (16, 24) |
| Cereal fibre | 7 (5, 10) | 6 (4, 8) | 6 (4, 10) | 9 (6, 12) | 8 (5, 10) | 6 (4, 8) | 9 (6, 12) | 9 (6, 12) | 13 (9, 17) | 10 (7, 13) |
| Fruit and vegetable fibre | 10 (7, 13) | 10 (7, 14) | 8 (6, 11) | 9 (6, 12) | 7 (5, 10) | 11 (9, 14) | 5 (4, 7) | 5 (3, 8) | 7 (5, 10) | 6 (4, 9) |
| Fruit fibre | 6 (4, 8) | 5 (3, 7) | 3 (2, 4) | 3 (2, 5) | 3 (2, 5) | 4 (3, 5) | 2 (2, 4) | 3 (2, 5) | 3 (1, 5) | 3 (1, 4) |
| Vegetable fibre | 4 (2, 5) | 5 (3, 7) | 5 (4, 7) | 5 (4, 7) | 4 (3, 5) | 8 (6, 10) | 3 (2, 4) | 2 (1, 3) | 4 (3, 6) | 3 (2, 5) |
| ^1^ United Kingdom-general includes participants recruited from general practices via both the Cambridge and Oxford centres. United Kingdom-health conscious includes participants recruited via postal methods, and includes a large proportion of vegetarians and health conscious people.  ^2^ Unavailable for Naples, Heidelberg and Potsdam.  ^3^ Unavailable for Potsdam.  ^4^ Unavailable for Umea.  ^5^ Unavailable for Denmark and Norway. | | | | | | | | | | |

**Supplementary table 5**. Covariate characteristics in each EPIC country.

|  | Country | | | | | | | | | |
| --- | --- | --- | --- | --- | --- | --- | --- | --- | --- | --- |
| Characteristic | Italy | Spain | UK-general^1^ | UK-health conscious^1^ | The Netherlands | Greece | Germany | Sweden | Denmark | Norway |
| Age, y (SD) | 50.5 (7.9) | 49.2 (8.0) | 57.6 (9.3) | 44.1 (14.4) | 49.1 (11.9) | 52.9 (12.5) | 50.5 (8.5) | 52.1 (10.8) | 56.6 (4.4) | 48.1 (4.3) |
| Women, n (%) | 31239 (69.0) | 25036 (62.4) | 18532 (60.0) | 37152 (78.0) | 27764 (74.5) | 15410 (59.7) | 29183 (57.8) | 29288 (57.1) | 28415 (53.3) | 36193 (100.0) |
| Body mass index, kg/m^2^ (SD) | 26.0 (4.0) | 28.2 (4.3) | 26.2 (4.1) | 23.4 (3.7) | 25.6 (4.0) | 28.7 (4.7) | 26.2 (4.3) | 25.6 (4.0) | 26.0 (4.0) | 24.4 (3.8) |
| Alcohol in current drinkers, g/day (SD) | 14.5 (17.9) | 16.6 (23.9) | 9.4 (13.5) | 10.0 (13.0) | 11.3 (15.7) | 10.0 (18.3) | 15.8 (19.9) | 8.4 (10.6) | 20.8 (21.3) | 3.7 (3.6) |
| Not current alcohol drinker, n (%) | 5113 (11.3) | 15004 (37.4) | 4288 (13.9) | 92 (0.2) | 2617 (7.0) | 6431 (24.9) | 2085 (4.1) | 0 (0.0) | 1145 (2.2) | 0 (0.0) |
| Smoking status and cigarettes/day, n (%) |  |  |  |  |  |  |  |  |  |  |
| Never smoker | 20581 (45.7) | 22295 (55.6) | 13940 (48.0) | 28930 (60.9) | 14277 (38.4) | 13823 (56.3) | 23243 (46.1) | 24938 (48.9) | 19052 (35.8) | 12223 (35.8) |
| Former smoker | 11984 (26.6) | 7025 (17.5) | 10830 (37.3) | 13492 (28.4) | 11570 (31.1) | 3924 (16.0) | 16554 (32.8) | 13703 (26.9) | 16032 (30.1) | 10627 (31.1) |
| Current smoker, <10 or number unknown | 4465 (9.9) | 4460 (11.1) | 1855 (6.4) | 2389 (5.0) | 3511 (9.5) | 1392 (5.7) | 3330 (6.6) | 3387 (6.7) | 5677 (10.7) | 1187 (3.5) |
| Current smoker, 10-19 | 4213 (9.4) | 2522 (6.3) | 1271 (4.4) | 1516 (3.2) | 4003 (10.8) | 1313 (5.3) | 3487 (6.9) | 5438 (10.7) | 6540 (12.3) | 6491 (19.0) |
| Current smoker, ≥20 | 3831 (8.5) | 3791 (9.5) | 1132 (3.9) | 1154 (2.4) | 3793 (10.2) | 4116 (16.8) | 3802 (7.5) | 3502 (6.9) | 5921 (11.1) | 3632 (10.6) |
| Highest level of education completed, n (%) |  |  |  |  |  |  |  |  |  |  |
| None or primary | 22260 (49.2) | 29306 (73.6) | 8233 (32.6) | 0 (0.0) | 5795 (15.6) | 15849 (61.5) | 12055 (23.9) | 17821 (34.9) | 17181 (32.3) | 8335 (23.0) |
| Secondary | 11236 (24.8) | 2596 (6.5) | 2408 (9.5) | 7315 (18.6) | 10497 (28.3) | 3346 (13.0) | 3423 (6.8) | 9403 (18.4) | 5362 (10.1) | 10417 (28.8) |
| Vocational or university | 11733 (25.9) | 7940 (19.9) | 14613 (57.9) | 32004 (81.4) | 20777 (56.1) | 6564 (25.5) | 35016 (69.4) | 23860 (46.7) | 30702 (57.7) | 17441 (48.2) |
| Cambridge physical activity index, n (%) |  |  |  |  |  |  |  |  |  |  |
| Inactive | 13316 (29.4) | 15386 (38.4) | 10256 (35.2) | 9207 (19.4) | 2558 (7.9) | 11612 (45.0) | 8500 (16.8) | 10727 (21.0) | 5708 (10.7) | 1338 (3.9) |
| Moderately inactive | 17270 (38.1) | 13337 (33.3) | 9332 (32.0) | 17264 (36.4) | 8287 (25.7) | 6845 (26.5) | 18529 (36.7) | 18322 (35.8) | 16289 (30.6) | 7743 (22.7) |
| Moderately active | 7988 (17.6) | 7080 (17.7) | 5608 (19.2) | 11951 (25.2) | 8501 (26.3) | 5291 (20.5) | 13257 (26.3) | 13388 (26.2) | 13023 (24.4) | 20684 (60.7) |
| Active | 6726 (14.9) | 4311 (10.8) | 3966 (13.6) | 9028 (19.0) | 12938 (40.1) | 2072 (8.0) | 10224 (20.2) | 8761 (17.1) | 18260 (34.3) | 4327 (12.7) |
| Employed or student, n (%) | 26259 (58.2) | NA^2^ | 16404 (53.5) | 33226 (70.8) | 21336 (58.7) | 14068 (54.5) | 35173 (71.3) | 36545 (71.3) | 42033 (79.2) | 29890 (88.6) |
| History of diabetes, n (%) | 942 (2.1) | 1947 (4.9) | 567 (2.0) | 563 (1.3) | 667 (1.8) | 1692 (6.6) | 1883 (3.7) | 997 (2.1) | 1000 (2.0) | 428 (1.5) |
| Previous hypertension, n (%) | 9163 (20.3) | 7973 (19.9) | 4019 (13.9) | 4702 (10.5) | 7993 (22.9) | 6030 (23.4) | 15120 (30.0) | 9054 (17.7) | 8183 (17.6) | 3619 (11.7) |
| Previous hyperlipidaemia, n (%) | 10940 (24.2) | 8034 (20.1) | 2301 (8.0) | 2175 (4.9) | 2941 (9.2) | 6201 (24.1) | 15272 (30.4) | 188 (0.8) | 3455 (11.3) | NA^2^ |
| Values are means (SD) or number (%), as indicated on the row.  ^1^United Kingdom-general includes participants recruited from general practices via both the Cambridge and Oxford centres. United Kingdom-health conscious includes participants recruited via postal methods, and includes a large proportion of vegetarians and health conscious people.  ^2^Data on this variable were not collected in this country | | | | | | | | | | |

**Supplementary table 6**. Hazard ratios^1^ (95% confidence intervals) for **ischaemic stroke** by overall fifths (relative to the bottom fifth of intake) and per unit higher observed intake of major foods and fibre in the EPIC study.

| Food | No. of cases | Fifth of intake^2^ | | | | Per unit difference (g/day)^2^ | P for trend^3^ |
| --- | --- | --- | --- | --- | --- | --- | --- |
|  |  | 2 | 3 | 4 | 5 |  |  |
| Red and processed meat | 4281 | 1.04 (0.92-1.16) | 1.11 (0.99-1.25) | 1.09 (0.97-1.23) | 1.19 (1.05-1.35) | 1.09 (1.01-1.17) | 0.026 |
| Red meat | 4281 | 0.94 (0.84-1.04) | 1.00 (0.90-1.11) | 1.03 (0.92-1.15) | 1.09 (0.97-1.24) | 1.07 (1.02-1.13) | 0.007 |
| Processed meat | 4281 | 0.97 (0.85-1.10) | 0.97 (0.86-1.10) | 1.00 (0.89-1.14) | 1.02 (0.89-1.16) | 1.01 (0.96-1.07) | 0.60 |
| Poultry meat | 4281 | 0.95 (0.86-1.05) | 1.02 (0.92-1.12) | 0.90 (0.81-0.99) | 0.97 (0.88-1.07) | 0.99 (0.96-1.03) | 0.66 |
| White fish^4^ | 3872 | 0.85 (0.76-0.95) | 0.90 (0.80-1.00) | 0.91 (0.83-1.01) | 0.95 (0.85-1.06) | 0.99 (0.97-1.02) | 0.61 |
| Fatty fish^5^ | 4059 | 0.98 (0.88-1.08) | 1.00 (0.90-1.11) | 1.06 (0.95-1.17) | 1.02 (0.92-1.12) | 1.02 (0.99-1.05) | 0.23 |
| Milk | 4281 | 0.89 (0.81-0.98) | 0.83 (0.75-0.93) | 0.81 (0.73-0.90) | 0.82 (0.74-0.91) | 0.97 (0.94-1.00) | 0.050 |
| Yogurt | 4281 | 0.98 (0.88-1.10) | 0.86 (0.77-0.95) | 0.90 (0.81-0.99) | 0.85 (0.77-0.93) | 0.95 (0.92-0.99) | 0.006 |
| Cheese | 4281 | 0.90 (0.81-0.99) | 0.90 (0.82-0.99) | 0.82 (0.74-0.90) | 0.90 (0.81-1.00) | 0.96 (0.93-1.00) | 0.048 |
| Eggs^6^ | 3772 | 1.02 (0.91-1.14) | 0.95 (0.85-1.06) | 1.03 (0.93-1.15) | 1.04 (0.93-1.15) | 1.03 (0.99-1.07) | 0.15 |
| Cereals and cereal products | 4281 | 0.96 (0.88-1.05) | 0.92 (0.83-1.01) | 0.88 (0.79-0.98) | 0.96 (0.86-1.07) | 0.99 (0.93-1.06) | 0.84 |
| Fruit and vegetables | 4281 | 0.89 (0.82-0.97) | 0.86 (0.79-0.95) | 0.79 (0.72-0.88) | 0.82 (0.73-0.93) | 0.94 (0.91-0.97) | <0.001 |
| Fruit | 4281 | 0.96 (0.88-1.05) | 0.89 (0.81-0.97) | 0.86 (0.78-0.95) | 0.86 (0.77-0.96) | 0.96 (0.94-0.99) | 0.001 |
| Vegetables | 4281 | 0.94 (0.86-1.03) | 0.91 (0.83-1.00) | 0.88 (0.80-0.97) | 0.87 (0.77-0.98) | 0.96 (0.93-1.00) | 0.024 |
| Legumes^7^ | 3511 | 0.99 (0.86-1.13) | 0.97 (0.86-1.09) | 1.02 (0.90-1.16) | 0.96 (0.83-1.10) | 0.99 (0.96-1.03) | 0.62 |
| Nuts and seeds^6^ | 3772 | 0.89 (0.77-1.03) | 0.90 (0.82-0.99) | 0.92 (0.82-1.03) | 0.94 (0.84-1.06) | 1.02 (0.97-1.06) | 0.44 |
| Total dietary fibre | 4281 | 0.96 (0.88-1.05) | 0.84 (0.76-0.92) | 0.83 (0.75-0.93) | 0.79 (0.70-0.90) | 0.88 (0.83-0.93) | <0.001 |
| Cereal fibre | 4281 | 0.90 (0.81-0.99) | 0.92 (0.83-1.01) | 0.80 (0.71-0.89) | 0.83 (0.74-0.93) | 0.96 (0.93-0.99) | 0.005 |
| Fruit and vegetable fibre | 4281 | 0.96 (0.88-1.04) | 0.93 (0.84-1.02) | 0.84 (0.76-0.94) | 0.83 (0.74-0.93) | 0.94 (0.91-0.97) | <0.001 |
| Fruit fibre | 4281 | 0.92 (0.84-1.01) | 0.89 (0.81-0.98) | 0.90 (0.81-0.99) | 0.83 (0.75-0.92) | 0.96 (0.94-0.98) | <0.001 |
| Vegetable fibre | 4281 | 0.96 (0.88-1.05) | 0.91 (0.82-1.00) | 0.93 (0.83-1.03) | 0.88 (0.78-0.99) | 0.97 (0.94-1.00) | 0.034 |
| ^1^ Hazard ratios are adjusted for age (continuous), smoking status and number of cigarettes per day (never smoker, former smoker, current smoker <10 cigs/d, current smoker 10-19 cigs/d, current smoker 20+ cigs/d, unknown), history of diabetes (yes, no, unknown), prior hypertension (yes, no , unknown), prior hyperlipidaemia (yes, no, unknown), Cambridge physical activity index (inactive, moderately inactive, moderately active, active, unknown), employment status (employed or student, not employed or student, unknown), level of education completed (none or primary, secondary, vocational or university, unknown), current alcohol consumption (non-drinkers and sex-specific fifths of intake among drinkers), body mass index (<22.5, 22.5-24.9, 25.0-27.4, 27.5-29.9, ≥30.0 kg/m^2^, unknown), and observed intake of energy (continuous), and stratified by sex and EPIC centre.  ^2^ For ranges of intake by observed fifths and unit sizes, please refer to Supplementary table 8.  ^3^ Tests of trend were performed using the observed intake (continuous).  ^4^ Unavailable for Naples, Heidelberg and Potsdam.  ^5^ Unavailable for Potsdam.  ^6^ Unavailable for Umea.  ^7^ Unavailable for Denmark and Norway. | | | | | | | |

**Supplementary table 7**. Hazard ratios^1^ (95% confidence intervals) for **haemorrhagic stroke** by overall fifths (relative to the bottom fifth of intake) and per unit higher observed intake of major foods and fibre in the EPIC study.

| Food | No. of cases | Fifth of intake^2^ | | | | Per unit difference (g/day)^2^ | P for trend^3^ |
| --- | --- | --- | --- | --- | --- | --- | --- |
|  |  | 2 | 3 | 4 | 5 |  |  |
| Red and processed meat | 1430 | 1.18 (0.98-1.42) | 1.04 (0.85-1.26) | 1.01 (0.83-1.23) | 1.03 (0.83-1.28) | 0.96 (0.84-1.09) | 0.53 |
| Red meat | 1430 | 1.01 (0.84-1.21) | 0.96 (0.80-1.17) | 1.06 (0.87-1.29) | 1.00 (0.81-1.23) | 0.99 (0.90-1.08) | 0.82 |
| Processed meat | 1430 | 1.04 (0.85-1.26) | 1.14 (0.94-1.39) | 0.97 (0.79-1.20) | 1.06 (0.85-1.32) | 0.96 (0.87-1.07) | 0.47 |
| Poultry meat | 1430 | 1.02 (0.86-1.22) | 1.03 (0.86-1.23) | 1.00 (0.84-1.19) | 0.97 (0.82-1.16) | 0.95 (0.89-1.01) | 0.082 |
| White fish^4^ | 1332 | 1.03 (0.85-1.24) | 1.13 (0.94-1.36) | 1.08 (0.90-1.28) | 0.93 (0.76-1.15) | 0.99 (0.94-1.04) | 0.60 |
| Fatty fish^5^ | 1377 | 1.01 (0.84-1.20) | 0.81 (0.68-0.97) | 0.96 (0.81-1.15) | 0.97 (0.81-1.16) | 0.99 (0.93-1.05) | 0.71 |
| Milk | 1430 | 0.98 (0.81-1.18) | 1.02 (0.85-1.23) | 1.15 (0.96-1.37) | 1.09 (0.91-1.31) | 1.03 (0.98-1.08) | 0.21 |
| Yogurt | 1430 | 0.94 (0.78-1.14) | 0.91 (0.75-1.09) | 0.91 (0.76-1.10) | 0.93 (0.78-1.10) | 0.95 (0.89-1.01) | 0.11 |
| Cheese | 1430 | 1.06 (0.90-1.24) | 0.97 (0.82-1.15) | 0.93 (0.78-1.11) | 0.88 (0.73-1.07) | 0.95 (0.89-1.01) | 0.095 |
| Eggs^6^ | 1310 | 0.92 (0.76-1.10) | 0.89 (0.73-1.08) | 0.92 (0.77-1.10) | 1.14 (0.95-1.36) | 1.10 (1.04-1.17) | 0.001 |
| Cereals and cereal products | 1430 | 0.86 (0.73-1.02) | 1.06 (0.90-1.25) | 1.03 (0.86-1.22) | 1.01 (0.83-1.22) | 1.03 (0.91-1.16) | 0.64 |
| Fruit and vegetables | 1430 | 0.98 (0.84-1.15) | 0.92 (0.78-1.10) | 0.98 (0.82-1.17) | 0.93 (0.76-1.14) | 0.99 (0.94-1.04) | 0.70 |
| Fruit | 1430 | 0.84 (0.71-0.98) | 0.84 (0.71-0.99) | 0.82 (0.69-0.98) | 0.88 (0.73-1.05) | 0.99 (0.96-1.03) | 0.57 |
| Vegetables | 1430 | 0.83 (0.70-0.98) | 1.03 (0.87-1.21) | 0.86 (0.72-1.02) | 1.00 (0.82-1.22) | 1.00 (0.95-1.05) | 0.95 |
| Legumes^7^ | 1096 | 1.04 (0.82-1.32) | 1.01 (0.81-1.24) | 1.16 (0.93-1.45) | 1.14 (0.89-1.46) | 1.02 (0.96-1.09) | 0.50 |
| Nuts and seeds^6^ | 1310 | 0.85 (0.67-1.08) | 0.91 (0.77-1.08) | 0.98 (0.82-1.18) | 0.85 (0.69-1.03) | 0.99 (0.93-1.06) | 0.82 |
| Total dietary fibre | 1430 | 0.86 (0.73-1.02) | 0.97 (0.82-1.16) | 0.97 (0.81-1.17) | 0.87 (0.70-1.07) | 0.98 (0.89-1.07) | 0.62 |
| Cereal fibre | 1430 | 0.90 (0.76-1.07) | 0.85 (0.71-1.01) | 0.89 (0.75-1.07) | 0.84 (0.68-1.02) | 0.98 (0.93-1.03) | 0.46 |
| Fruit and vegetable fibre | 1430 | 0.98 (0.83-1.15) | 0.92 (0.78-1.10) | 0.98 (0.82-1.17) | 0.95 (0.78-1.14) | 0.99 (0.94-1.04) | 0.60 |
| Fruit fibre | 1430 | 0.85 (0.72-1.00) | 0.86 (0.73-1.01) | 0.88 (0.74-1.04) | 0.90 (0.76-1.08) | 0.99 (0.96-1.03) | 0.78 |
| Vegetable fibre | 1430 | 0.81 (0.68-0.95) | 0.86 (0.73-1.02) | 0.87 (0.73-1.04) | 0.90 (0.74-1.09) | 0.99 (0.94-1.03) | 0.58 |
| ^1^ Hazard ratios are adjusted for age (continuous), smoking status and number of cigarettes per day (never smoker, former smoker, current smoker <10 cigs/d, current smoker 10-19 cigs/d, current smoker 20+ cigs/d, unknown), history of diabetes (yes, no, unknown), prior hypertension (yes, no , unknown), prior hyperlipidaemia (yes, no, unknown), Cambridge physical activity index (inactive, moderately inactive, moderately active, active, unknown), employment status (employed or student, not employed or student, unknown), level of education completed (none or primary, secondary, vocational or university, unknown), current alcohol consumption (non-drinkers and sex-specific fifths of intake among drinkers), body mass index (<22.5, 22.5-24.9, 25.0-27.4, 27.5-29.9, ≥30.0 kg/m^2^, unknown), and observed intake of energy (continuous), and stratified by sex and EPIC centre.  ^2^ For ranges of intake by observed fifths and unit sizes, please refer to Supplementary table 8.  ^3^ Tests of trend were performed using the observed intake (continuous).  ^4^ Unavailable for Naples, Heidelberg and Potsdam.  ^5^ Unavailable for Potsdam.  ^6^ Unavailable for Umea.  ^7^ Unavailable for Denmark and Norway. | | | | | | | |

**Supplementary table 8**. Hazard ratios^1^ (95% confidence intervals) for **total stroke** by overall fifths (relative to the bottom fifth of intake) and per unit higher observed intake of major foods and fibre in the EPIC study.

| Food | No. of cases | Fifth of intake^2^ | | | | Per unit difference (g/day)^2^ | P for trend^3^ |
| --- | --- | --- | --- | --- | --- | --- | --- |
|  |  | 2 | 3 | 4 | 5 |  |  |
| Red and processed meat | 7378 | 1.06 (0.98-1.16) | 1.06 (0.97-1.16) | 1.07 (0.98-1.17) | 1.18 (1.07-1.30) | 1.08 (1.02-1.15) | 0.005 |
| Red meat | 7378 | 0.97 (0.89-1.05) | 0.99 (0.91-1.08) | 1.02 (0.94-1.12) | 1.09 (0.99-1.19) | 1.06 (1.02-1.10) | 0.004 |
| Processed meat | 7378 | 0.99 (0.90-1.09) | 1.03 (0.94-1.13) | 1.03 (0.94-1.13) | 1.07 (0.97-1.18) | 1.03 (0.98-1.07) | 0.27 |
| Poultry meat | 7378 | 0.97 (0.89-1.05) | 1.00 (0.92-1.08) | 0.93 (0.86-1.01) | 0.94 (0.87-1.02) | 0.98 (0.95-1.00) | 0.077 |
| White fish^4^ | 6855 | 0.92 (0.84-1.00) | 0.93 (0.86-1.01) | 0.94 (0.87-1.02) | 0.92 (0.85-1.01) | 0.98 (0.96-1.00) | 0.13 |
| Fatty fish^5^ | 7100 | 0.94 (0.86-1.01) | 0.93 (0.86-1.00) | 0.99 (0.91-1.07) | 0.98 (0.91-1.06) | 1.01 (0.99-1.04) | 0.38 |
| Milk | 7378 | 0.91 (0.84-0.98) | 0.88 (0.82-0.96) | 0.89 (0.82-0.96) | 0.90 (0.84-0.98) | 1.00 (0.98-1.02) | 0.88 |
| Yogurt | 7378 | 0.98 (0.90-1.06) | 0.89 (0.82-0.96) | 0.91 (0.84-0.99) | 0.88 (0.81-0.94) | 0.95 (0.93-0.98) | <0.001 |
| Cheese | 7378 | 0.96 (0.89-1.03) | 0.91 (0.84-0.97) | 0.86 (0.79-0.93) | 0.90 (0.83-0.98) | 0.96 (0.93-0.99) | 0.004 |
| Eggs^6^ | 6748 | 0.95 (0.87-1.03) | 0.94 (0.87-1.02) | 0.98 (0.90-1.06) | 1.04 (0.96-1.12) | 1.04 (1.01-1.07) | 0.006 |
| Cereals and cereal products | 7378 | 0.93 (0.87-1.00) | 0.93 (0.86-1.00) | 0.91 (0.85-0.99) | 0.93 (0.85-1.01) | 0.97 (0.92-1.03) | 0.35 |
| Fruit and vegetables | 7378 | 0.90 (0.84-0.97) | 0.88 (0.82-0.95) | 0.84 (0.77-0.90) | 0.79 (0.72-0.87) | 0.94 (0.92-0.97) | <0.001 |
| Fruit | 7378 | 0.93 (0.86-0.99) | 0.86 (0.80-0.93) | 0.84 (0.78-0.91) | 0.85 (0.79-0.93) | 0.97 (0.95-0.99) | <0.001 |
| Vegetables | 7378 | 0.92 (0.85-0.98) | 0.95 (0.88-1.02) | 0.87 (0.81-0.94) | 0.86 (0.78-0.94) | 0.96 (0.94-0.98) | <0.001 |
| Legumes^7^ | 5545 | 0.96 (0.87-1.07) | 0.97 (0.89-1.07) | 1.04 (0.94-1.15) | 0.94 (0.84-1.05) | 0.99 (0.96-1.02) | 0.53 |
| Nuts and seeds^6^ | 6748 | 0.86 (0.78-0.96) | 0.89 (0.82-0.95) | 0.91 (0.84-0.99) | 0.87 (0.80-0.95) | 1.00 (0.97-1.03) | 0.83 |
| Total dietary fibre | 7378 | 0.92 (0.86-0.99) | 0.85 (0.78-0.91) | 0.84 (0.78-0.92) | 0.76 (0.69-0.84) | 0.88 (0.85-0.92) | <0.001 |
| Cereal fibre | 7378 | 0.91 (0.84-0.98) | 0.91 (0.84-0.98) | 0.83 (0.77-0.90) | 0.84 (0.77-0.92) | 0.96 (0.94-0.98) | <0.001 |
| Fruit and vegetable fibre | 7378 | 0.96 (0.89-1.03) | 0.91 (0.84-0.98) | 0.87 (0.81-0.94) | 0.82 (0.75-0.89) | 0.94 (0.92-0.96) | <0.001 |
| Fruit fibre | 7378 | 0.89 (0.83-0.96) | 0.88 (0.82-0.94) | 0.87 (0.81-0.94) | 0.84 (0.78-0.91) | 0.97 (0.95-0.98) | <0.001 |
| Vegetable fibre | 7378 | 0.94 (0.87-1.01) | 0.92 (0.86-1.00) | 0.92 (0.85-0.99) | 0.84 (0.77-0.92) | 0.96 (0.94-0.98) | <0.001 |
| ^1^ Hazard ratios are adjusted for age (continuous), smoking status and number of cigarettes per day (never smoker, former smoker, current smoker <10 cigs/d, current smoker 10-19 cigs/d, current smoker 20+ cigs/d, unknown), history of diabetes (yes, no, unknown), prior hypertension (yes, no , unknown), prior hyperlipidaemia (yes, no, unknown), Cambridge physical activity index (inactive, moderately inactive, moderately active, active, unknown), employment status (employed or student, not employed or student, unknown), level of education completed (none or primary, secondary, vocational or university, unknown), current alcohol consumption (non-drinkers and sex-specific fifths of intake among drinkers), body mass index (<22.5, 22.5-24.9, 25.0-27.4, 27.5-29.9, ≥30.0 kg/m^2^, unknown), and observed intake of energy (continuous), and stratified by sex and EPIC centre.  ^2^ For ranges of intake by observed fifths and unit sizes, please refer to Supplementary table 8.  ^3^ Tests of trend were performed using the observed intake (continuous).  ^4^ Unavailable for Naples, Heidelberg and Potsdam.  ^5^ Unavailable for Potsdam.  ^6^ Unavailable for Umea.  ^7^ Unavailable for Denmark and Norway. | | | | | | | |

**
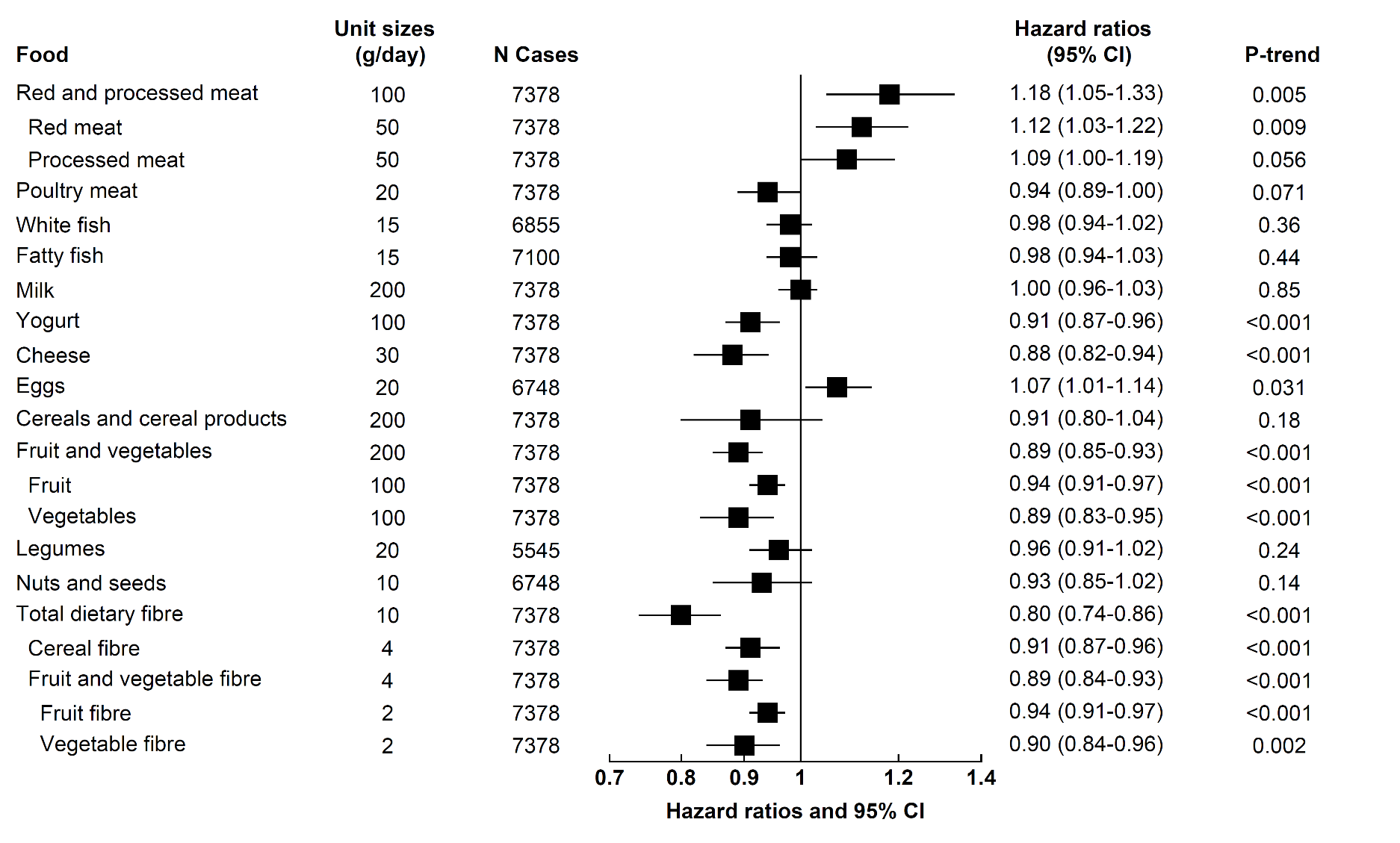
**

**Supplementary figure 1:** Hazard ratios (95% confidence intervals) for **total stroke** per unit higher calibrated intake of major foods and fibre in the EPIC study.

Unit sizes represent approximate differences in mean 24 hour recall intake between participants in the lowest and highest fifths of observed intake. Hazard ratios are adjusted for age (continuous), smoking status and number of cigarettes per day (never smoker, former smoker, current smoker <10 cigs/d, current smoker 10-19 cigs/d, current smoker 20+ cigs/d, unknown), history of diabetes (yes, no, unknown), prior hypertension (yes, no , unknown), prior hyperlipidaemia (yes, no, unknown), Cambridge physical activity index (inactive, moderately inactive, moderately active, active, unknown), employment status (employed or student, not employed or student, unknown), level of education completed (none or primary, secondary, vocational or university, unknown), current alcohol consumption (non-drinkers and sex-specific fifths of intake among drinkers), body mass index (<22.5, 22.5-24.9, 25.0-27.4, 27.5-29.9, ≥30.0 kg/m^2^, unknown), and calibrated intake of energy (continuous), and stratified by sex and EPIC centre.

**Supplementary table 9**. Ranges of intake by fifths of observed intake and unit sizes of selected major foods and fibre in the EPIC study.

| Foods | Fifth of intake, range and median (g/day)^1^ | | | | | Per unit^1^ (g/day) |
| --- | --- | --- | --- | --- | --- | --- |
|  | 1 | 2 | 3 | 4 | 5 |  |
| Red and processed meat | <31.9, 13.5 | >=31.9 to <54.6, 43.9 | >=54.6 to <77.5, 65.5 | >=77.5 to <109.4, 91.4 | >=109.4, 138.6 | 100 |
| Red meat | <11.9, 3.7 | >=11.9 to <25.6, 18.8 | >=25.6 to <42.2, 33.1 | >=42.2 to <67.8, 53.3 | >=67.8, 88.1 | 50 |
| Processed meat | <6.6, 1.7 | >=6.6 to <18.0, 12.6 | >=18.0 to <30.9, 23.9 | >=30.9 to <50.5, 39.1 | >=50.5, 69.3 | 50 |
| Poultry meat | <3.4, 0.0 | >=3.4 to <9.5, 6.6 | >=9.5 to <16.1, 13.5 | >=16.1 to <28.5, 20.4 | >=28.5, 44.6 | 20 |
| White fish^2^ | <1.0, 0.0 | >=1.0 to <8.1, 3.9 | >=8.1 to <16.0, 11.2 | >=16.0 to <27.0, 19.1 | >=27.0, 45.1 | 15 |
| Fatty fish^3^ | <0.8, 0.0 | >=0.8 to <4.1, 2.1 | >=4.1 to <8.3, 7.0 | >=8.3 to <16.4, 12.6 | >=16.4, 27.8 | 15 |
| Milk | <17.1, 1.6 | >=17.1 to <114.1, 55.7 | >=114.1 to <202.6, 151.4 | >=202.6 to <374.1, 292.7 | >=374.1, 490.8 | 200 |
| Yogurt | <0.0, 0.0 | >=0.0 to <15.2, 4.9 | >=15.2 to <44.9, 25.0 | >=44.9 to <103.1, 62.5 | >=103.1, 158.8 | 100 |
| Cheese | <11.4, 5.0 | >=11.4 to <21.0, 16.9 | >=21.0 to <34.6, 27.1 | >=34.6 to <55.1, 42.2 | >=55.1, 76.5 | 30 |
| Eggs^4^ | <5.8, 3.0 | >=5.8 to <10.0, 7.8 | >=10.0 to <16.6, 13.7 | >=16.6 to <25.1, 21.5 | >=25.1, 35.6 | 20 |
| Cereals and cereal products | <132.5, 104.3 | >=132.5 to <177.3, 155.3 | >=177.3 to <224.7, 200.3 | >=224.7 to <291.6, 253.1 | >=291.6, 358.2 | 200 |
| Fruit and vegetables | <210.5, 154.5 | >=210.5 to <312.1, 260.6 | >=312.1 to <430.3, 367.8 | >=430.3 to <608.3, 506.2 | >=608.3, 776.7 | 200 |
| Fruit | <87.7, 49.7 | >=87.7 to <149.3, 117.5 | >=149.3 to <231.0, 186.8 | >=231.0 to <343.7, 276.2 | >=343.7, 449.2 | 100 |
| Vegetables | <92.2, 66.2 | >=92.2 to <136.1, 113.9 | >=136.1 to <191.6, 161.3 | >=191.6 to <285.9, 230.6 | >=285.9, 380.4 | 100 |
| Legumes^5^ | <0.7, 0.0 | >=0.7 to <5.5, 2.4 | >=5.5 to <11.6, 8.1 | >=11.6 to <30.8, 16.6 | >=30.8, 45.7 | 20 |
| Nuts and seeds^4^ | <0.0, 0.0 | >=0.0 to <0.4, 0.2 | >=0.4 to <1.6, 0.8 | >=1.6 to <4.5, 3.0 | >=4.5, 10.2 | 10 |
| Total dietary fibre | <16.3, 13.8 | >=16.3 to <20.0, 18.3 | >=20.0 to <23.6, 21.8 | >=23.6 to <28.6, 25.8 | >=28.6, 32.9 | 10 |
| Cereal fibre | <5.0, 3.7 | >=5.0 to <7.1, 6.1 | >=7.1 to <9.4, 8.2 | >=9.4 to <12.8, 10.9 | >=12.8, 15.6 | 4 |
| Fruit and vegetable fibre | <4.5, 3.3 | >=4.5 to <6.5, 5.6 | >=6.5 to <8.7, 7.6 | >=8.7 to <11.8, 10.0 | >=11.8, 14.5 | 4 |
| Fruit fibre | <1.6, 0.9 | >=1.6 to <2.7, 2.1 | >=2.7 to <4.0, 3.3 | >=4.0 to <6.0, 4.9 | >=6.0, 8.0 | 2 |
| Vegetable fibre | <2.1, 1.5 | >=2.1 to <3.2, 2.7 | >=3.2 to <4.4, 3.8 | >=4.4 to <6.3, 5.2 | >=6.3, 8.0 | 2 |
| ^1^ Refers to the observed fifths and size of per unit difference used in longitudinal analyses of foods and stroke risk. Unit sizes represent approximate differences in mean 24 hour recall intake between participants in the lowest and highest fifths of observed intake.  ^2^ Unavailable for Naples, Heidelberg and Potsdam.  ^3^ Unavailable for Potsdam.  ^4^ Unavailable for Umea.  ^5^ Unavailable for Denmark and Norway. | | | | | |  |

**Supplementary table 10**. Hazard ratios (95% confidence intervals) for stroke per unit higher^1^ calibrated intake of **subtypes of fruit and vegetables** in the EPIC study.

| Major subtypes of fruits and vegetables | Unit sizes (g/day) | No. of cases | **Ischaemic stroke** | P for trend^2^ | No. of cases | **Haemorrhagic stroke** | P for trend^2^ |
| --- | --- | --- | --- | --- | --- | --- | --- |
| Fruit subtypes |  |  |  |  |  |  |  |
| Citrus fruits | 50 | 4281 | 0.95 (0.90-0.99) | 0.028 | 1430 | 1.00 (0.92-1.08) | 0.96 |
| Hard fruits (apples and pears) | 50 | 4281 | 0.95 (0.92-0.98) | 0.001 | 1430 | 0.97 (0.92-1.03) | 0.33 |
| Bananas | 50 | 4281 | 0.89 (0.82-0.98) | 0.018 | 1430 | 0.85 (0.73-0.99) | 0.043 |
|  |  |  |  |  |  |  |  |
| Vegetable subtypes |  |  |  |  |  |  |  |
| Fruiting vegetables^3^ | 50 | 4274 | 0.92 (0.85-0.99) | 0.031 | 1403 | 1.02 (0.89-1.16) | 0.77 |
| Leafy vegetables^4^ | 50 | 3765 | 1.11 (0.92-1.35) | 0.27 | 1283 | 1.26 (0.88-1.80) | 0.21 |
| Cabbages^5^ | 50 | 3772 | 0.93 (0.77-1.11) | 0.41 | 1310 | 1.01 (0.72-1.40) | 0.96 |
| Root vegetables | 50 | 4281 | 0.80 (0.69-0.93) | 0.003 | 1430 | 0.84 (0.67-1.06) | 0.15 |
| ^1^ Hazard ratios are adjusted for age (continuous), smoking status and number of cigarettes per day (never smoker, former smoker, current smoker <10 cigs/d, current smoker 10-19 cigs/d, current smoker 20+ cigs/d, unknown), history of diabetes (yes, no, unknown), prior hypertension (yes, no , unknown), prior hyperlipidaemia (yes, no, unknown), Cambridge physical activity index (inactive, moderately inactive, moderately active, active, unknown), employment status (employed or student, not employed or student, unknown), level of education completed (none or primary, secondary, vocational or university, unknown), current alcohol consumption (non-drinkers and sex-specific fifths of intake among drinkers), body mass index (<22.5, 22.5-24.9, 25.0-27.4, 27.5-29.9, ≥30.0 kg/m^2^, unknown), and calibrated intake of energy (continuous), and stratified by sex and EPIC centre. Unit sizes represent approximate differences in mean 24 hour recall intake between participants in the lowest and highest fifths of observed intake.  ^2^ Tests of trend were performed using the calibrated intake (continuous).  ^3^ Unavailable for Norway.  ^4^ Unavailable for Umea and Norway.  ^5^ Unavailable for Umea. | | | | | | | |

**Supplementary table 11**. Hazard ratios (95% confidence intervals) for stroke per unit higher^1^ calibrated intake of major foods, **adjusted for fibre**^2^, in the EPIC study.

|  | Food | Unit sizes (g/day) | **Ischaemic stroke** | P for trend^3^ | **Haemorrhagic stroke** | P for trend^3^ |  |
| --- | --- | --- | --- | --- | --- | --- | --- |
|  | Red and processed meat | 100 | 1.07 (0.92-1.25) | 0.36 | 1.02 (0.77-1.34) | 0.90 |  |
|  | Red meat | 50 | 1.10 (0.99-1.23) | 0.076 | 0.97 (0.79-1.19) | 0.79 |  |
|  | Processed meat | 50 | 1.00 (0.89-1.13) | 0.98 | 1.08 (0.88-1.34) | 0.46 |  |
|  | Poultry meat | 20 | 0.99 (0.91-1.07) | 0.73 | 0.94 (0.82-1.07) | 0.35 |  |
|  | White fish^4^ | 15 | 0.98 (0.93-1.03) | 0.39 | 1.03 (0.94-1.13) | 0.52 |  |
|  | Fatty fish^5^ | 15 | 1.00 (0.95-1.06) | 0.91 | 0.96 (0.86-1.07) | 0.42 |  |
|  | Milk | 200 | 0.93 (0.89-0.98) | 0.004 | 1.07 (0.99-1.15) | 0.090 |  |
|  | Yogurt | 100 | 0.92 (0.86-0.98) | 0.008 | 0.93 (0.82-1.04) | 0.21 |  |
|  | Cheese | 30 | 0.89 (0.81-0.97) | 0.012 | 0.89 (0.76-1.05) | 0.17 |  |
|  | Eggs^6^ | 20 | 1.04 (0.95-1.13) | 0.39 | 1.25 (1.09-1.43) | 0.002 |  |
|  | Cereals and cereal products | 200 | 0.95 (0.80-1.13) | 0.55 | 1.03 (0.76-1.41) | 0.83 |  |
|  | Fruit and vegetables | 200 | 0.88 (0.82-0.93) | <0.0001 | 1.00 (0.90-1.11) | 0.97 |  |
|  | Fruit | 100 | 0.93 (0.90-0.97) | 0.0002 | 0.98 (0.92-1.05) | 0.58 |  |
|  | Vegetables | 100 | 0.87 (0.80-0.96) | 0.003 | 1.03 (0.89-1.20) | 0.65 |  |
|  | Legumes^7^ | 20 | 0.99 (0.92-1.07) | 0.88 | 1.04 (0.92-1.19) | 0.52 |  |
|  | Nuts and seeds^6^ | 10 | 1.00 (0.88-1.13) | 0.98 | 0.89 (0.72-1.10) | 0.30 |  |
| ^1^ Hazard ratios are adjusted for age (continuous), smoking status and number of cigarettes per day (never smoker, former smoker, current smoker <10 cigs/d, current smoker 10-19 cigs/d, current smoker 20+ cigs/d, unknown), history of diabetes (yes, no, unknown), prior hypertension (yes, no , unknown), prior hyperlipidaemia (yes, no, unknown), Cambridge physical activity index (inactive, moderately inactive, moderately active, active, unknown), employment status (employed or student, not employed or student, unknown), level of education completed (none or primary, secondary, vocational or university, unknown), current alcohol consumption (non-drinkers and sex-specific fifths of intake among drinkers), body mass index (<22.5, 22.5-24.9, 25.0-27.4, 27.5-29.9, ≥30.0 kg/m^2^, unknown), and calibrated intake of energy (continuous), and stratified by sex and EPIC centre. Unit sizes represent approximate differences in mean 24 hour recall intake between participants in the lowest and highest fifths of observed intake.  ^2^ For animal foods (red and processed meat, poultry, fish, milk, yogurt, cheese, eggs), legumes, nuts and seeds, the analyses was adjusted for total dietary fibre. For Cereals and cereal products, the analysis was adjusted for fruit and vegetable fibre. For fruit and vegetables, the analyses were adjusted for cereal fibre.  ^3^ Tests of trend were performed using the calibrated intake (continuous).  ^4^ Unavailable for Naples, Heidelberg and Potsdam.  ^5^ Unavailable for Potsdam.  ^6^ Unavailable for Umea.  ^7^ Unavailable for Denmark and Norway. | | | | | | | |

**Supplementary table 12**. Hazard ratios^1^ (95% confidence intervals) for stroke, **with mutual adjustment of all major foods**, in the EPIC study.

|  | Food | Unit sizes (g/day)^2^ | **Ischaemic stroke**  (2,593 cases) | P for trend^3^ | **Haemorrhagic stroke**  (878 cases) | P for trend^3^ |  |
| --- | --- | --- | --- | --- | --- | --- | --- |
|  | Red and processed meat | 100 | 0.93 (0.76, 1.13) | 0.45 | 0.85 (0.59, 1.22) | 0.36 |  |
|  | Poultry meat | 20 | 1.00 (0.92, 1.09) | 0.99 | 0.91 (0.78, 1.06) | 0.23 |  |
|  | White fish^4^ | 15 | 0.98 (0.93, 1.02) | 0.32 | 1.06 (0.96, 1.16) | 0.28 |  |
|  | Fatty fish^5^ | 15 | 1.01 (0.95, 1.08) | 0.67 | 0.97 (0.86, 1.10) | 0.63 |  |
|  | Milk | 200 | 0.91 (0.85, 0.97) | 0.003 | 1.09 (0.97, 1.22) | 0.13 |  |
|  | Yogurt | 100 | 0.92 (0.84, 1.01) | 0.06 | 0.82 (0.68, 0.98) | 0.02 |  |
|  | Cheese | 30 | 0.86 (0.76, 0.98) | 0.01 | 0.86 (0.68, 1.07) | 0.18 |  |
|  | Eggs^6^ | 20 | 1.04 (0.94, 1.14) | 0.44 | 1.15 (0.96, 1.37) | 0.11 |  |
|  | Cereals and cereal products | 200 | 1.01 (0.81, 1.27) | 0.92 | 1.21 (0.80, 1.82) | 0.36 |  |
|  | Fruit and vegetables | 200 | 0.89 (0.83, 0.97) | 0.007 | 1.04 (0.91, 1.19) | 0.59 |  |
|  | Legumes^7^ | 20 | 0.94 (0.87, 1.02) | 0.11 | 1.05 (0.93, 1.19) | 0.44 |  |
|  | Nuts and seeds^6^ | 10 | 0.98 (0.85, 1.13) | 0.78 | 0.87 (0.68, 1.11) | 0.27 |  |
| ^1^ Hazard ratios are adjusted for all foods listed in the rows above, age (continuous), smoking status and number of cigarettes per day (never smoker, former smoker, current smoker <10 cigs/d, current smoker 10-19 cigs/d, current smoker 20+ cigs/d, unknown), history of diabetes (yes, no, unknown), prior hypertension (yes, no , unknown), prior hyperlipidaemia (yes, no, unknown), Cambridge physical activity index (inactive, moderately inactive, moderately active, active, unknown), employment status (employed or student, not employed or student, unknown), level of education completed (none or primary, secondary, vocational or university, unknown), current alcohol consumption (non-drinkers and sex-specific fifths of intake among drinkers), body mass index (<22.5, 22.5-24.9, 25.0-27.4, 27.5-29.9, ≥30.0 kg/m^2^, unknown), and calibrated intake of energy (continuous), and stratified by sex and EPIC centre.  ^2^ Unit sizes represent approximate differences in mean 24 hour recall intake between participants in the lowest and highest fifths of observed intake.  ^3^ Tests of trend were performed using the calibrated intake (continuous). | | | | | | | |

**Supplementary table 13**. Hazard ratios (95% confidence intervals) for **subarachnoid haemorrhage** and **intracerebral haemorrhage** per unit higher^1^ calibrated intake of major foods and fibre in the EPIC study.

|  | Food | Unit sizes (g/day) | **Subarachnoid haemorrhage**  (up to 476 cases) | P for trend^2^ | **Intracerebral haemorrhage**  (up to 954 cases) | P for trend^2^ |  |
| --- | --- | --- | --- | --- | --- | --- | --- |
|  | Red and processed meat | 100 | 1.25 (0.80-1.98) | 0.33 | 0.92 (0.66-1.29) | 0.63 |  |
|  | Red meat | 50 | 1.37 (1.00-1.88) | 0.052 | 0.82 (0.64-1.05) | 0.11 |  |
|  | Processed meat | 50 | 0.86 (0.58-1.25) | 0.42 | 1.21 (0.94-1.55) | 0.13 |  |
|  | Poultry meat | 20 | 0.98 (0.78-1.23) | 0.86 | 0.91 (0.77-1.08) | 0.30 |  |
|  | White fish^3^ | 15 | 1.04 (0.87-1.24) | 0.70 | 1.03 (0.92-1.15) | 0.62 |  |
|  | Fatty fish^4^ | 15 | 0.92 (0.76-1.13) | 0.43 | 0.97 (0.86-1.11) | 0.69 |  |
|  | Milk | 200 | 1.08 (0.95-1.24) | 0.24 | 1.06 (0.97-1.17) | 0.20 |  |
|  | Yogurt | 100 | 0.96 (0.78-1.18) | 0.71 | 0.91 (0.79-1.05) | 0.19 |  |
|  | Cheese | 30 | 1.05 (0.80-1.37) | 0.75 | 0.83 (0.68-1.01) | 0.058 |  |
|  | Eggs^5^ | 20 | 1.29 (1.02-1.63) | 0.031 | 1.23 (1.04-1.46) | 0.016 |  |
|  | Cereals and cereal products | 200 | 0.95 (0.53-1.69) | 0.86 | 1.08 (0.74-1.57) | 0.69 |  |
|  | Fruit and vegetables | 200 | 1.07 (0.90-1.28) | 0.44 | 0.96 (0.85-1.10) | 0.59 |  |
|  | Fruit | 100 | 1.01 (0.91-1.13) | 0.81 | 0.97 (0.89-1.05) | 0.41 |  |
|  | Vegetables | 100 | 1.09 (0.85-1.39) | 0.50 | 1.00 (0.83-1.20) | 1.00 |  |
|  | Legumes^6^ | 20 | 1.00 (0.79-1.27) | 0.99 | 1.08 (0.93-1.24) | 0.30 |  |
|  | Nuts and seeds^5^ | 10 | 0.96 (0.69-1.33) | 0.80 | 0.85 (0.64-1.11) | 0.23 |  |
|  | Total dietary fibre | 10 | 1.10 (0.81-1.49) | 0.55 | 0.93 (0.74-1.16) | 0.51 |  |
|  | Cereal fibre | 4 | 1.01 (0.83-1.22) | 0.94 | 0.93 (0.81-1.06) | 0.27 |  |
|  | Fruit and vegetable fibre | 4 | 1.08 (0.90-1.29) | 0.40 | 0.95 (0.83-1.08) | 0.43 |  |
|  | Fruit fibre | 2 | 1.00 (0.90-1.12) | 0.94 | 0.98 (0.91-1.06) | 0.66 |  |
|  | Vegetable fibre | 2 | 1.11 (0.87-1.43) | 0.39 | 0.88 (0.73-1.07) | 0.21 |  |
| ^1^ Hazard ratios are adjusted for age (continuous), smoking status and number of cigarettes per day (never smoker, former smoker, current smoker <10 cigs/d, current smoker 10-19 cigs/d, current smoker 20+ cigs/d, unknown), history of diabetes (yes, no, unknown), prior hypertension (yes, no , unknown), prior hyperlipidaemia (yes, no, unknown), Cambridge physical activity index (inactive, moderately inactive, moderately active, active, unknown), employment status (employed or student, not employed or student, unknown), level of education completed (none or primary, secondary, vocational or university, unknown), current alcohol consumption (non-drinkers and sex-specific fifths of intake among drinkers), body mass index (<22.5, 22.5-24.9, 25.0-27.4, 27.5-29.9, ≥30.0 kg/m^2^, unknown), and calibrated intake of energy (continuous), and stratified by sex and EPIC centre. Unit sizes represent approximate differences in mean 24 hour recall intake between participants in the lowest and highest fifths of observed intake.  ^2^ Tests of trend were performed using the calibrated intake (continuous).  ^3^ Unavailable for Naples, Heidelberg and Potsdam.  ^4^ Unavailable for Potsdam.  ^5^ Unavailable for Umea.  ^6^ Unavailable for Denmark and Norway. | | | | | | | |

**Supplementary table 14**. **Adjusted mean systolic blood pressure^1^** (mm Hg; 95% confidence intervals) by overall fifths of observed intake of major foods and fibre in up to 293,092 participants in the EPIC study.

| Food | No. of observations | Fifth of intake | | | | | P for trend^2^ |  |
| --- | --- | --- | --- | --- | --- | --- | --- | --- |
|  |  | 1 | 2 | 3 | 4 | 5 |  | |
| Red and processed meat | 293092 | 130.1 (129.9-130.2) | 131.2 (131.0-131.3) | 131.8 (131.7-132.0) | 132.2 (132.1-132.4) | 133.1 (133.0-133.0) | <0.001 | |
| Red meat | 293092 | 130.4 (130.2-130.6) | 131.2 (131.0-131.4) | 131.7 (131.6-131.9) | 132.2 (132.1-132.4) | 132.7 (132.6-132.9) | <0.001 | |
| Processed meat | 293092 | 130.0 (129.8-130.1) | 131.1 (131.1-131.4) | 132.1 (131.9-132.2) | 132.6 (132.4-132.7) | 133.2 (133.1-133.4) | <0.001 | |
| Poultry meat | 293092 | 131.3 (131.1-131.4) | 131.5 (131.3-131.6) | 131.8 (131.7-132.0) | 132.1 (132.0-132.2) | 132.4 (132.2-132.5) | <0.001 | |
| White fish^3^ | 253839 | 131.4 (131.2-131.6) | 131.1 (130.9-131.2) | 131.4 (131.2-131.5) | 131.7 (131.5-131.9) | 132.0 (131.8-132.2) | <0.001 | |
| Fatty fish^4^ | 268271 | 131.6 (131.5-131.8) | 131.6 (131.4-131.7) | 131.5 (131.3-131.6) | 131.6 (131.4-131.7) | 132.0 (131.8-132.2) | <0.001 | |
| Milk | 293092 | 132.0 (131.8-132.1) | 131.9 (131.8-132.1) | 131.7 (131.5-131.8) | 131.7 (131.5-131.8) | 131.8 (131.7-132.0) | 0.17 | |
| Yogurt | 293092 | 132.8 (132.6-132.9) | 132.1 (132.0-132.3) | 131.9 (131.7-132.0) | 131.7 (131.5-131.8) | 130.9 (130.8-131.1) | <0.001 | |
| Cheese | 293092 | 132.2 (132.0-132.4) | 132.4 (132.2-132.5) | 131.9 (131.8-132.1) | 131.5 (131.4-131.6) | 131.3 (131.2-131.4) | <0.001 | |
| Eggs^5^ | 268579 | 131.2 (131.0-131.4) | 131.6 (131.4-131.7) | 131.4 (131.3-131.6) | 131.8 (131.7-132.0) | 131.9 (131.8-132.1) | <0.001 | |
| Cereals and cereal products | 293092 | 132.0 (131.9-132.2) | 132.0 (131.8-132.1) | 131.9 (131.8-132.0) | 131.7 (131.6-131.9) | 131.4 (131.3-131.6) | <0.001 | |
| Fruit and vegetables | 293092 | 132.5 (132.3-132.6) | 131.9 (131.8-132.0) | 131.6 (131.5-131.8) | 131.6 (131.5-131.8) | 131.4 (131.3-131.6) | <0.001 | |
| Fruit | 293092 | 132.2 (132.1-132.4) | 131.8 (131.6-131.9) | 131.7 (131.6-131.8) | 131.8 (131.7-132.0) | 131.6 (131.4-131.7) | <0.001 | |
| Vegetables | 293092 | 132.6 (132.5-132.8) | 132.1 (131.9-132.2) | 131.7 (131.6-131.8) | 131.4 (131.3-131.6) | 131.1 (131.0-131.3) | <0.001 | |
| Legumes^6^ | 239822 | 130.4 (130.2-130.6) | 130.3 (130.2-130.5) | 130.6 (130.4-130.7) | 130.8 (130.7-131.0) | 130.6 (130.4-130.8) | 0.086 | |
| Nuts and seeds^5^ | 268579 | 132.5 (132.4-132.7) | 131.7 (131.5-131.8) | 131.7 (131.5-131.8) | 131.2 (131.0-131.4) | 130.8 (130.6-130.9) | <0.001 | |
| Total dietary fibre | 293092 | 132.5 (132.3-132.6) | 132.1 (131.9-132.2) | 131.9 (131.7-132.0) | 131.5 (131.4-131.7) | 131.1 (131.0-131.3) | <0.001 | |
| Cereal fibre | 293092 | 132.1 (132.0-132.3) | 132.2 (132.0-132.3) | 131.9 (131.8-132.1) | 131.8 (131.6-131.9) | 131.1 (131.0-131.3) | <0.001 | |
| Fruit and vegetable fibre | 293092 | 132.5 (132.3-132.6) | 131.9 (131.7-132.0) | 131.7 (131.6-131.9) | 131.6 (131.5-131.8) | 131.4 (131.2-131.5) | <0.001 | |
| Fruit fibre | 293092 | 132.3 (132.1-132.4) | 131.9 (131.7-132.0) | 131.7 (131.6-131.9) | 131.5 (131.4-131.7) | 131.7 (131.5-131.8) | <0.001 | |
| Vegetable fibre | 293092 | 132.5 (132.4-132.7) | 132.0 (131.8-132.1) | 131.7 (131.5-131.8) | 131.6 (131.5-131.8) | 131.2 (131.0-131.3) | <0.001 | |
| ^1^ Means are adjusted for age (continuous), sex and EPIC centre  ^2^ Based on median food intake in each category.  ^3^ Unavailable for Naples, Heidelberg and Potsdam.  ^4^ Unavailable for Potsdam.  ^5^ Unavailable for Umea.  ^6^ Unavailable for Denmark and Norway. | | | | | | | | |

**Supplementary table 15**. **Adjusted mean non-high density lipoprotein cholesterol^1^** (mmol/l; 95% confidence intervals) by overall fifths of observed intake of major foods and fibre in up to 16467 participants in the EPIC study.

| Food | No. of observations | Fifth of intake | | | | | P for trend^2^ | |  |
| --- | --- | --- | --- | --- | --- | --- | --- | --- | --- |
|  |  | 1 | 2 | 3 | 4 | 5 | |  | |
| Red and processed meat | 16467 | 4.40 (4.36-4.44) | 4.44 (4.40-4.48) | 4.45 (4.41-4.49) | 4.54 (4.50-4.58) | 4.58 (4.54-4.62) | | <0.001 | |
| Red meat | 16467 | 4.42 (4.38-4.46) | 4.41 (4.37-4.45) | 4.48 (4.44-4.52) | 4.52 (4.48-4.56) | 4.57 (4.53-4.61) | | <0.001 | |
| Processed meat | 16467 | 4.41 (4.36-4.45) | 4.46 (4.42-4.50) | 4.50 (4.46-4.54) | 4.49 (4.45-4.53) | 4.55 (4.51-4.59) | | <0.001 | |
| Poultry meat | 16467 | 4.43 (4.39-4.47) | 4.47 (4.43-4.51) | 4.48 (4.44-4.52) | 4.49 (4.45-4.53) | 4.54 (4.50-4.58) | | <0.001 | |
| White fish^3^ | 14212 | 4.45 (4.40-4.50) | 4.42 (4.37-4.47) | 4.48 (4.43-4.53) | 4.49 (4.44-4.53) | 4.52 (4.47-4.57) | | 0.016 | |
| Fatty fish^4^ | 15259 | 4.38 (4.33-4.43) | 4.44 (4.39-4.49) | 4.44 (4.39-4.49) | 4.48 (4.43-4.53) | 4.48 (4.43-4.53) | | 0.006 | |
| Milk | 16467 | 4.50 (4.46-4.54) | 4.53 (4.49-4.57) | 4.48 (4.45-4.52) | 4.43 (4.39-4.47) | 4.46 (4.42-4.50) | | 0.020 | |
| Yogurt | 16467 | 4.50 (4.46-4.54) | 4.54 (4.49-4.59) | 4.51 (4.47-4.55) | 4.46 (4.42-4.50) | 4.41 (4.37-4.45) | | <0.001 | |
| Cheese | 16467 | 4.55 (4.50-4.59) | 4.53 (4.49-4.57) | 4.45 (4.41-4.49) | 4.44 (4.40-4.48) | 4.44 (4.40-4.48) | | <0.001 | |
| Eggs^5^ | 15525 | 4.57 (4.53-4.61) | 4.53 (4.49-4.57) | 4.48 (4.44-4.52) | 4.52 (4.47-4.56) | 4.47 (4.43-4.51) | | 0.002 | |
| Cereals and cereal products | 16467 | 4.55 (4.51-4.59) | 4.50 (4.46-4.54) | 4.46 (4.42-4.50) | 4.48 (4.45-4.52) | 4.42 (4.38-4.46) | | <0.001 | |
| Fruit and vegetables | 16467 | 4.57 (4.52-4.61) | 4.50 (4.46-4.54) | 4.45 (4.41-4.49) | 4.48 (4.44-4.52) | 4.42 (4.37-4.46) | | <0.001 | |
| Fruit | 16467 | 4.57 (4.52-4.61) | 4.47 (4.43-4.51) | 4.46 (4.42-4.50) | 4.47 (4.43-4.51) | 4.44 (4.40-4.48) | | <0.001 | |
| Vegetables | 16467 | 4.54 (4.50-4.58) | 4.50 (4.46-4.54) | 4.48 (4.44-4.52) | 4.46 (4.42-4.50) | 4.43 (4.38-4.47) | | 0.001 | |
| Legumes^6^ | 14360 | 4.58 (4.52-4.64) | 4.58 (4.52-4.63) | 4.62 (4.57-4.67) | 4.59 (4.54-4.63) | 4.55 (4.50-4.60) | | 0.25 | |
| Nuts and seeds^5^ | 15525 | 4.54 (4.50-4.58) | 4.51 (4.43-4.58) | 4.52 (4.48-4.57) | 4.53 (4.48-4.57) | 4.46 (4.42-4.50) | | 0.008 | |
| Total dietary fibre | 16467 | 4.54 (4.50-4.58) | 4.52 (4.48-4.56) | 4.47 (4.43-4.51) | 4.47 (4.43-4.51) | 4.41 (4.37-4.45) | | <0.001 | |
| Cereal fibre | 16467 | 4.54 (4.50-4.58) | 4.50 (4.46-4.54) | 4.48 (4.44-4.52) | 4.47 (4.43-4.51) | 4.41 (4.37-4.45) | | <0.001 | |
| Fruit and vegetable fibre | 16467 | 4.55 (4.51-4.59) | 4.50 (4.46-4.53) | 4.46 (4.42-4.50) | 4.47 (4.43-4.51) | 4.43 (4.39-4.47) | | <0.001 | |
| Fruit fibre | 16467 | 4.55 (4.51-4.59) | 4.51 (4.47-4.55) | 4.45 (4.41-4.49) | 4.46 (4.42-4.49) | 4.45 (4.41-4.49) | | 0.001 | |
| Vegetable fibre | 16467 | 4.53 (4.49-4.57) | 4.50 (4.46-4.54) | 4.48 (4.44-4.52) | 4.46 (4.42-4.50) | 4.44 (4.40-4.48) | | 0.007 | |
| ^1^ Means are adjusted for age (continuous), sex and EPIC centre  ^2^ Based on median food intake in each category.  ^3^ Unavailable for Naples, Heidelberg and Potsdam.  ^4^ Unavailable for Potsdam.  ^5^ Unavailable for Umea.  ^6^ Unavailable for Denmark and Norway. | | | | | | | | | |

**Supplementary table 16**. Hazard ratios (95% confidence intervals) for **ischaemic stroke** per unit higher^1^ calibrated intake of major foods and fibre, with **additional adjustment for systolic blood pressure**, in up to 293,092 participants in the EPIC study.

|  | Food | Unit sizes (g/day) | No. of cases | Without adjustment | P for trend^2^ | With adjustment | P for trend^2^ | % attenuation (95% CI)^3^ |
| --- | --- | --- | --- | --- | --- | --- | --- | --- |
|  | Red and processed meat | 100 | 3588 | 1.11 (0.94-1.31) | 0.23 | 1.09 (0.92-1.29) | 0.31 | 16.2 (-401.7,434.1) |
|  | Red meat | 50 | 3588 | 1.08 (0.95-1.23) | 0.26 | 1.08 (0.94-1.23) | 0.27 | 2.62 (-352.3,357.5) |
|  | Processed meat | 50 | 3588 | 1.03 (0.91-1.17) | 0.63 | 1.02 (0.89-1.15) | 0.82 | 51.2 (-12805,12908) |
|  | Poultry meat | 20 | 3588 | 0.98 (0.89-1.08) | 0.72 | 0.99 (0.90-1.09) | 0.85 | 46.5 (-1551,1644) |
|  | White fish^4^ | 15 | 3323 | 0.95 (0.89-1.02) | 0.16 | 0.95 (0.89-1.01) | 0.13 | -9.91 (-821.0,801.2) |
|  | Fatty fish^5^ | 15 | 3384 | 0.99 (0.92-1.06) | 0.73 | 0.99 (0.92-1.06) | 0.79 | 25.7 (-433.3,484.6) |
|  | Milk | 200 | 3588 | 0.96 (0.91-1.01) | 0.098 | 0.96 (0.91-1.01) | 0.085 | -4.26 (-190.9,182.4) |
|  | Yogurt | 100 | 3588 | 0.91 (0.85-0.97) | 0.007 | 0.92 (0.86-0.99) | 0.018 | 13.2 (-503.2,529.5) |
|  | Cheese | 30 | 3588 | 0.88 (0.80-0.97) | 0.011 | 0.89 (0.81-0.98) | 0.018 | 7.01 (-11.8,25.8) |
|  | Eggs^6^ | 20 | 3094 | 1.07 (0.97-1.17) | 0.19 | 1.07 (0.97-1.17) | 0.20 | 2.20 (-121.9,126.3) |
|  | Cereals and cereal products | 200 | 3588 | 0.97 (0.81-1.18) | 0.78 | 1.00 (0.82-1.20) | 0.96 | 81.6 (-904.4,1068) |
|  | Fruit and vegetables | 200 | 3588 | 0.82 (0.76-0.89) | <0.0001 | 0.83 (0.77-0.90) | <0.0001 | 6.80 (2.88,10.7) |
|  | Fruit | 100 | 3588 | 0.91 (0.86-0.95) | 0.0001 | 0.91 (0.87-0.95) | 0.0001 | 4.34 (0.25,8.42) |
|  | Vegetables | 100 | 3588 | 0.80 (0.72-0.89) | <0.0001 | 0.82 (0.74-0.91) | 0.0002 | 11.7 (4.21,19.3) |
|  | Legumes^7^ | 20 | 2826 | 0.98 (0.86-1.13) | 0.82 | 0.98 (0.86-1.13) | 0.81 | -10.1 (-195.4,175.1) |
|  | Nuts and seeds^6^ | 10 | 3094 | 0.95 (0.81-1.11) | 0.51 | 0.97 (0.83-1.13) | 0.66 | 34.9 (-684.5,754.2) |
|  | Total dietary fibre | 10 | 3588 | 0.74 (0.66-0.83) | <0.0001 | 0.75 (0.67-0.85) | <0.0001 | 6.40 (2.26,10.5) |
|  | Cereal fibre | 4 | 3588 | 0.91 (0.85-0.97) | 0.006 | 0.91 (0.85-0.98) | 0.012 | 8.92 (-23.4,41.3) |
|  | Fruit and vegetable fibre | 4 | 3588 | 0.83 (0.77-0.89) | <0.0001 | 0.83 (0.77-0.90) | <0.0001 | 5.16 (1.82,8.51) |
|  | Fruit fibre | 2 | 3588 | 0.91 (0.87-0.95) | 0.0001 | 0.91 (0.87-0.95) | 0.0001 | 3.28 (-0.31,6.87) |
|  | Vegetable fibre | 2 | 3588 | 0.82 (0.74-0.91) | 0.0002 | 0.84 (0.76-0.93) | 0.0006 | 9.22 (-5.81,24.3) |
| ^1^ Hazard ratios are adjusted for age (continuous), smoking status and number of cigarettes per day (never smoker, former smoker, current smoker <10 cigs/d, current smoker 10-19 cigs/d, current smoker 20+ cigs/d, unknown), history of diabetes (yes, no, unknown), prior hypertension (yes, no , unknown), prior hyperlipidaemia (yes, no, unknown), Cambridge physical activity index (inactive, moderately inactive, moderately active, active, unknown), employment status (employed or student, not employed or student, unknown), level of education completed (none or primary, secondary, vocational or university, unknown), current alcohol consumption (non-drinkers and sex-specific fifths of intake among drinkers), body mass index (<22.5, 22.5-24.9, 25.0-27.4, 27.5-29.9, ≥30.0 kg/m^2^, unknown), and calibrated intake of energy (continuous), and stratified by sex and EPIC centre. Analyses restricted to participants who provided readings of systolic blood pressure. Unit sizes represent approximate differences in mean 24 hour recall intake between participants in the lowest and highest fifths of observed intake.  ^2^ Tests of trend were performed using the calibrated intake (continuous).  ^3^ Represents the potential explanatory role of SBP, estimated as (βfood - βfood+SBP)/ βfood x100, with 95% confidence intervals estimated using a bootstrap method with 1000 resamplings.  ^4^ Unavailable for Naples, Heidelberg and Potsdam.  ^5^ Unavailable for Potsdam.  ^6^ Unavailable for Umea.  ^7^ Unavailable for Denmark and Norway. | | | | | | | | |

**Supplementary table 17**. Hazard ratios (95% confidence intervals) for **haemorrhagic stroke** per unit higher^1^ calibrated intake of major foods and fibre, with **additional adjustment for systolic blood pressure**, in up to 293,092 participants in the EPIC study.

|  | Food | Unit sizes (g/day) | No. of cases | Without adjustment | P for trend^2^ | With adjustment | P for trend^2^ | % attenuation (95% CI)^3^ |
| --- | --- | --- | --- | --- | --- | --- | --- | --- |
|  | Red and processed meat | 100 | 1173 | 1.16 (0.86-1.56) | 0.33 | 1.13 (0.84-1.52) | 0.42 | 16.9 (-1736,1769) |
|  | Red meat | 50 | 1173 | 1.10 (0.87-1.38) | 0.42 | 1.09 (0.87-1.38) | 0.46 | 6.75 (-1278,1291) |
|  | Processed meat | 50 | 1173 | 1.19 (0.96-1.49) | 0.12 | 1.17 (0.93-1.46) | 0.17 | 12.0 (-140.2,164.2) |
|  | Poultry meat | 20 | 1173 | 1.00 (0.85-1.17) | 0.95 | 1.00 (0.86-1.18) | 0.96 | 190.1 (-812.9,1193) |
|  | White fish^4^ | 15 | 1097 | 1.06 (0.93-1.21) | 0.39 | 1.05 (0.92-1.20) | 0.48 | 17.4 (-1078,1112) |
|  | Fatty fish^5^ | 15 | 1122 | 0.98 (0.86-1.12) | 0.80 | 0.99 (0.87-1.13) | 0.86 | 28.5 (-148.1,205.2) |
|  | Milk | 200 | 1173 | 1.06 (0.98-1.15) | 0.13 | 1.06 (0.98-1.15) | 0.14 | 1.92 (-77.8,81.6) |
|  | Yogurt | 100 | 1173 | 0.90 (0.79-1.02) | 0.093 | 0.92 (0.81-1.04) | 0.17 | 18.5 (-642.7,679.8) |
|  | Cheese | 30 | 1173 | 0.90 (0.76-1.06) | 0.20 | 0.91 (0.77-1.08) | 0.27 | 13.9 (-215.6,243.4) |
|  | Eggs^6^ | 20 | 1057 | 1.30 (1.12-1.52) | 0.0007 | 1.30 (1.11-1.52) | 0.0009 | 1.12 (-4.50,6.73) |
|  | Cereals and cereal products | 200 | 1173 | 1.04 (0.74-1.45) | 0.83 | 1.07 (0.77-1.50) | 0.68 | -88.1 (-1131,954.9) |
|  | Fruit and vegetables | 200 | 1173 | 0.95 (0.84-1.07) | 0.41 | 0.97 (0.85-1.09) | 0.59 | 34.6 (-2454,2523) |
|  | Fruit | 100 | 1173 | 0.97 (0.90-1.05) | 0.44 | 0.98 (0.90-1.05) | 0.51 | 16.9 (-496.6,530.3) |
|  | Vegetables | 100 | 1173 | 0.95 (0.80-1.13) | 0.57 | 0.99 (0.83-1.17) | 0.89 | 75.9 (-3155,3307) |
|  | Legumes^7^ | 20 | 866 | 1.08 (0.92-1.27) | 0.35 | 1.08 (0.91-1.27) | 0.37 | 2.35 (-465.6,470.3) |
|  | Nuts and seeds^6^ | 10 | 1057 | 0.71 (0.53-0.95) | 0.019 | 0.73 (0.55-0.97) | 0.029 | 6.94 (-57.2,71.1) |
|  | Total dietary fibre | 10 | 1173 | 0.88 (0.72-1.08) | 0.22 | 0.91 (0.75-1.11) | 0.36 | 25.0 (-617.1,667.0) |
|  | Cereal fibre | 4 | 1173 | 0.94 (0.83-1.05) | 0.26 | 0.95 (0.85-1.07) | 0.39 | 22.6 (-1154,1199) |
|  | Fruit and vegetable fibre | 4 | 1173 | 0.94 (0.83-1.06) | 0.30 | 0.95 (0.84-1.07) | 0.41 | 21.9 (-885.2,929.0) |
|  | Fruit fibre | 2 | 1173 | 0.98 (0.91-1.05) | 0.56 | 0.98 (0.91-1.06) | 0.62 | 16.0 (-3024,3056) |
|  | Vegetable fibre | 2 | 1173 | 0.88 (0.74-1.05) | 0.15 | 0.91 (0.76-1.08) | 0.27 | 22.9 (-1273,1318) |
| ^1^ Hazard ratios are adjusted for age (continuous), smoking status and number of cigarettes per day (never smoker, former smoker, current smoker <10 cigs/d, current smoker 10-19 cigs/d, current smoker 20+ cigs/d, unknown), history of diabetes (yes, no, unknown), prior hypertension (yes, no , unknown), prior hyperlipidaemia (yes, no, unknown), Cambridge physical activity index (inactive, moderately inactive, moderately active, active, unknown), employment status (employed or student, not employed or student, unknown), level of education completed (none or primary, secondary, vocational or university, unknown), current alcohol consumption (non-drinkers and sex-specific fifths of intake among drinkers), body mass index (<22.5, 22.5-24.9, 25.0-27.4, 27.5-29.9, ≥30.0 kg/m^2^, unknown), and calibrated intake of energy (continuous), and stratified by sex and EPIC centre. Analyses restricted to participants who provided readings of systolic blood pressure. Unit sizes represent approximate differences in mean 24 hour recall intake between participants in the lowest and highest fifths of observed intake.  ^2^ Tests of trend were performed using the calibrated intake (continuous).  ^3^ Represents the potential explanatory role of SBP, estimated as (βfood - βfood+SBP)/ βfood x100, with 95% confidence intervals estimated using a bootstrap method with 1000 resamplings.  ^4^ Unavailable for Naples, Heidelberg and Potsdam.  ^5^ Unavailable for Potsdam.  ^6^ Unavailable for Umea.  ^7^ Unavailable for Denmark and Norway. | | | | | | | | |

**Supplementary table 18**. Hazard ratios (95% confidence intervals) for stroke per unit higher^1^ calibrated intake of major foods and fibre, after **excluding the first 4 years of follow up** in the EPIC study.

|  | Food | Unit sizes (g/day) | **Ischaemic stroke**  (up to 3497 cases) | P for trend^2^ | **Haemorrhagic stroke**  (up to 1122 cases) | P for trend^2^ |  |
| --- | --- | --- | --- | --- | --- | --- | --- |
|  | Red and processed meat | 100 | 1.16 (0.98-1.37) | 0.091 | 0.93 (0.68-1.28) | 0.67 |  |
|  | Red meat | 50 | 1.14 (1.01-1.29) | 0.032 | 0.91 (0.73-1.15) | 0.44 |  |
|  | Processed meat | 50 | 1.04 (0.91-1.18) | 0.55 | 1.04 (0.82-1.32) | 0.76 |  |
|  | Poultry meat | 20 | 0.99 (0.91-1.08) | 0.82 | 0.93 (0.80-1.09) | 0.39 |  |
|  | White fish^3^ | 15 | 0.98 (0.93-1.03) | 0.38 | 1.03 (0.92-1.14) | 0.64 |  |
|  | Fatty fish^4^ | 15 | 0.98 (0.92-1.04) | 0.50 | 0.97 (0.86-1.09) | 0.60 |  |
|  | Milk | 200 | 0.93 (0.89-0.98) | 0.008 | 1.10 (1.01-1.19) | 0.032 |  |
|  | Yogurt | 100 | 0.93 (0.86-1.00) | 0.040 | 0.99 (0.87-1.13) | 0.86 |  |
|  | Cheese | 30 | 0.86 (0.78-0.95) | 0.003 | 0.89 (0.74-1.07) | 0.20 |  |
|  | Eggs^5^ | 20 | 1.03 (0.94-1.13) | 0.54 | 1.22 (1.04-1.42) | 0.013 |  |
|  | Cereals and cereal products | 200 | 0.95 (0.79-1.16) | 0.64 | 1.04 (0.73-1.48) | 0.81 |  |
|  | Fruit and vegetables | 200 | 0.87 (0.81-0.94) | <0.001 | 1.00 (0.89-1.12) | 0.99 |  |
|  | Fruit | 100 | 0.93 (0.90-0.97) | 0.001 | 0.97 (0.90-1.04) | 0.38 |  |
|  | Vegetables | 100 | 0.86 (0.78-0.95) | 0.002 | 1.06 (0.90-1.25) | 0.48 |  |
|  | Legumes^6^ | 20 | 0.98 (0.91-1.06) | 0.63 | 1.08 (0.96-1.22) | 0.22 |  |
|  | Nuts and seeds^5^ | 10 | 1.02 (0.89-1.16) | 0.78 | 0.92 (0.73-1.16) | 0.48 |  |
|  | Total dietary fibre | 10 | 0.77 (0.69-0.87) | <0.001 | 0.95 (0.78-1.17) | 0.64 |  |
|  | Cereal fibre | 4 | 0.89 (0.83-0.96) | 0.002 | 0.94 (0.83-1.07) | 0.34 |  |
|  | Fruit and vegetable fibre | 4 | 0.88 (0.82-0.94) | <0.001 | 1.00 (0.88-1.13) | 0.98 |  |
|  | Fruit fibre | 2 | 0.94 (0.90-0.98) | 0.002 | 0.98 (0.91-1.05) | 0.56 |  |
|  | Vegetable fibre | 2 | 0.88 (0.80-0.97) | 0.011 | 0.99 (0.84-1.17) | 0.91 |  |
| ^1^ Hazard ratios are adjusted for age (continuous), smoking status and number of cigarettes per day (never smoker, former smoker, current smoker <10 cigs/d, current smoker 10-19 cigs/d, current smoker 20+ cigs/d, unknown), history of diabetes (yes, no, unknown), prior hypertension (yes, no , unknown), prior hyperlipidaemia (yes, no, unknown), Cambridge physical activity index (inactive, moderately inactive, moderately active, active, unknown), employment status (employed or student, not employed or student, unknown), level of education completed (none or primary, secondary, vocational or university, unknown), current alcohol consumption (non-drinkers and sex-specific fifths of intake among drinkers), body mass index (<22.5, 22.5-24.9, 25.0-27.4, 27.5-29.9, ≥30.0 kg/m^2^, unknown), and calibrated intake of energy (continuous), and stratified by sex and EPIC centre. Unit sizes represent approximate differences in mean 24 hour recall intake between participants in the lowest and highest fifths of observed intake.  ^2^ Tests of trend were performed using the calibrated intake (continuous).  ^3^ Unavailable for Naples, Heidelberg and Potsdam.  ^4^ Unavailable for Potsdam.  ^5^ Unavailable for Umea.  ^6^ Unavailable for Denmark and Norway. | | | | | | | |

**Supplementary table 19**. Hazard ratios (95% confidence intervals) for stroke per unit higher^1^ calibrated intake of major foods and fibre, **restricting to the first 10 years of follow up** in the EPIC study.

|  | Food | Unit sizes (g/day) | **Ischaemic stroke**  (up to 2778 cases) | P for trend^2^ | **Haemorrhagic stroke**  (up to 1025 cases) | P for trend^2^ |  |
| --- | --- | --- | --- | --- | --- | --- | --- |
|  | Red and processed meat | 100 | 1.13 (0.95-1.36) | 0.17 | 1.21 (0.89-1.65) | 0.23 |  |
|  | Red meat | 50 | 1.13 (0.98-1.29) | 0.084 | 1.09 (0.86-1.39) | 0.45 |  |
|  | Processed meat | 50 | 1.07 (0.93-1.24) | 0.34 | 1.17 (0.92-1.49) | 0.21 |  |
|  | Poultry meat | 20 | 1.01 (0.92-1.11) | 0.86 | 0.93 (0.79-1.10) | 0.39 |  |
|  | White fish^3^ | 15 | 1.01 (0.95-1.08) | 0.67 | 1.01 (0.89-1.14) | 0.89 |  |
|  | Fatty fish^4^ | 15 | 1.02 (0.95-1.10) | 0.54 | 0.96 (0.84-1.10) | 0.54 |  |
|  | Milk | 200 | 0.98 (0.93-1.04) | 0.50 | 1.10 (1.00-1.20) | 0.047 |  |
|  | Yogurt | 100 | 0.89 (0.82-0.96) | 0.005 | 0.88 (0.76-1.02) | 0.091 |  |
|  | Cheese | 30 | 0.91 (0.81-1.02) | 0.10 | 0.85 (0.70-1.02) | 0.087 |  |
|  | Eggs^5^ | 20 | 1.12 (1.01-1.24) | 0.034 | 1.40 (1.20-1.63) | <0.0001 |  |
|  | Cereals and cereal products | 200 | 0.83 (0.67-1.04) | 0.11 | 0.77 (0.53-1.12) | 0.17 |  |
|  | Fruit and vegetables | 200 | 0.88 (0.81-0.95) | 0.002 | 1.00 (0.88-1.14) | 0.95 |  |
|  | Fruit | 100 | 0.95 (0.90-0.99) | 0.022 | 0.97 (0.90-1.05) | 0.49 |  |
|  | Vegetables | 100 | 0.84 (0.75-0.94) | 0.003 | 1.08 (0.90-1.28) | 0.41 |  |
|  | Legumes^6^ | 20 | 0.95 (0.86-1.05) | 0.28 | 1.03 (0.88-1.21) | 0.72 |  |
|  | Nuts and seeds^5^ | 10 | 0.95 (0.81-1.11) | 0.51 | 0.88 (0.68-1.13) | 0.32 |  |
|  | Total dietary fibre | 10 | 0.74 (0.65-0.85) | <0.0001 | 0.94 (0.76-1.17) | 0.60 |  |
|  | Cereal fibre | 4 | 0.88 (0.81-0.96) | 0.003 | 0.89 (0.78-1.02) | 0.088 |  |
|  | Fruit and vegetable fibre | 4 | 0.88 (0.81-0.96) | 0.003 | 1.00 (0.88-1.14) | 0.95 |  |
|  | Fruit fibre | 2 | 0.94 (0.90-0.99) | 0.020 | 0.98 (0.90-1.06) | 0.60 |  |
|  | Vegetable fibre | 2 | 0.87 (0.78-0.97) | 0.016 | 1.03 (0.86-1.24) | 0.72 |  |
| ^1^ Hazard ratios are adjusted for age (continuous), smoking status and number of cigarettes per day (never smoker, former smoker, current smoker <10 cigs/d, current smoker 10-19 cigs/d, current smoker 20+ cigs/d, unknown), history of diabetes (yes, no, unknown), prior hypertension (yes, no , unknown), prior hyperlipidaemia (yes, no, unknown), Cambridge physical activity index (inactive, moderately inactive, moderately active, active, unknown), employment status (employed or student, not employed or student, unknown), level of education completed (none or primary, secondary, vocational or university, unknown), current alcohol consumption (non-drinkers and sex-specific fifths of intake among drinkers), body mass index (<22.5, 22.5-24.9, 25.0-27.4, 27.5-29.9, ≥30.0 kg/m^2^, unknown), and calibrated intake of energy (continuous), and stratified by sex and EPIC centre. Unit sizes represent approximate differences in mean 24 hour recall intake between participants in the lowest and highest fifths of observed intake.  ^2^ Tests of trend were performed using the calibrated intake (continuous).  ^3^ Unavailable for Naples, Heidelberg and Potsdam.  ^4^ Unavailable for Potsdam.  ^5^ Unavailable for Umea.  ^6^ Unavailable for Denmark and Norway. | | | | | | | |

**Supplementary table 20**: Hazard ratios (95% confidence intervals) for **ischaemic stroke** per unit higher^1^ calibrated intake of major foods and fibre, **stratified** **by age at recruitment**, showing results from independent subset analyses in the EPIC study.

| Food | Unit sizes (g/day) | <55 years | | |  | 55-64 years | | |  | ≥65 years | | | Measures of heterogeneity^3^ | |
| --- | --- | --- | --- | --- | --- | --- | --- | --- | --- | --- | --- | --- | --- | --- |
|  |  | No. of cases | HR (95% CI) | P for trend^2^ |  | No. of cases | HR (95% CI) | P for trend^2^ |  | No. of cases | HR (95% CI) | P for trend^2^ | P-het | *I^2^* (%) |
| Red and processed meat | 100 | 1200 | 1.16 (0.90-1.50) | 0.24 |  | 2188 | 1.19 (0.96-1.48) | 0.11 |  | 893 | 0.99 (0.68-1.43) | 0.95 | 0.68 | 0 |
| Red meat | 50 | 1200 | 1.15 (0.95-1.39) | 0.15 |  | 2188 | 1.16 (0.99-1.36) | 0.058 |  | 893 | 1.03 (0.80-1.34) | 0.80 | 0.74 | 0 |
| Processed meat | 50 | 1200 | 1.05 (0.86-1.28) | 0.65 |  | 2188 | 1.07 (0.91-1.26) | 0.41 |  | 893 | 0.96 (0.72-1.29) | 0.80 | 0.82 | 0 |
| Poultry meat | 20 | 1200 | 0.97 (0.83-1.14) | 0.70 |  | 2188 | 1.02 (0.91-1.14) | 0.77 |  | 893 | 0.97 (0.83-1.14) | 0.71 | 0.85 | 0 |
| White fish^4^ | 15 | 1046 | 0.99 (0.91-1.09) | 0.91 |  | 1958 | 0.97 (0.91-1.04) | 0.40 |  | 868 | 0.97 (0.87-1.08) | 0.59 | 0.91 | 0 |
| Fatty fish^5^ | 15 | 1127 | 1.00 (0.90-1.12) | 0.96 |  | 2060 | 0.99 (0.92-1.08) | 0.86 |  | 872 | 1.03 (0.91-1.15) | 0.65 | 0.89 | 0 |
| Milk | 200 | 1200 | 0.98 (0.90-1.06) | 0.60 |  | 2188 | 0.90 (0.85-0.96) | 0.002 |  | 893 | 1.04 (0.93-1.17) | 0.46 | 0.064 | 64 |
| Yogurt | 100 | 1200 | 0.84 (0.73-0.95) | 0.007 |  | 2188 | 0.95 (0.87-1.03) | 0.20 |  | 893 | 0.91 (0.78-1.06) | 0.23 | 0.31 | 16 |
| Cheese | 30 | 1200 | 0.86 (0.73-1.01) | 0.065 |  | 2188 | 0.89 (0.79-1.02) | 0.084 |  | 893 | 0.94 (0.73-1.20) | 0.60 | 0.84 | 0 |
| Eggs^6^ | 20 | 988 | 1.12 (0.96-1.31) | 0.15 |  | 1891 | 0.99 (0.88-1.12) | 0.93 |  | 893 | 1.08 (0.90-1.29) | 0.42 | 0.46 | 0 |
| Cereals and cereal products | 200 | 1200 | 1.02 (0.74-1.40) | 0.92 |  | 2188 | 0.99 (0.78-1.26) | 0.93 |  | 893 | 0.80 (0.52-1.22) | 0.29 | 0.63 | 0 |
| Fruit and vegetables | 200 | 1200 | 0.89 (0.80-1.00) | 0.050 |  | 2188 | 0.83 (0.76-0.91) | <0.0001 |  | 893 | 1.01 (0.86-1.18) | 0.95 | 0.13 | 52 |
| Fruit | 100 | 1200 | 0.95 (0.89-1.02) | 0.14 |  | 2188 | 0.90 (0.86-0.95) | 0.0001 |  | 893 | 1.02 (0.93-1.13) | 0.69 | 0.075 | 61 |
| Vegetables | 100 | 1200 | 0.89 (0.76-1.04) | 0.14 |  | 2188 | 0.84 (0.75-0.95) | 0.005 |  | 893 | 0.95 (0.75-1.19) | 0.64 | 0.64 | 0 |
| Legumes^7^ | 20 | 972 | 0.97 (0.85-1.10) | 0.64 |  | 1661 | 0.92 (0.82-1.03) | 0.14 |  | 878 | 1.07 (0.91-1.26) | 0.42 | 0.33 | 10 |
| Nuts and seeds^6^ | 10 | 988 | 1.01 (0.83-1.24) | 0.89 |  | 1891 | 0.92 (0.76-1.12) | 0.40 |  | 893 | 1.05 (0.80-1.37) | 0.73 | 0.68 | 0 |
| Total dietary fibre | 10 | 1200 | 0.75 (0.62-0.91) | 0.004 |  | 2188 | 0.77 (0.67-0.89) | 0.0005 |  | 893 | 0.83 (0.64-1.08) | 0.17 | 0.84 | 0 |
| Cereal fibre | 4 | 1200 | 0.91 (0.81-1.02) | 0.099 |  | 2188 | 0.95 (0.87-1.04) | 0.27 |  | 893 | 0.73 (0.60-0.88) | 0.001 | 0.043 | 68 |
| Fruit and vegetable fibre | 4 | 1200 | 0.88 (0.78-0.99) | 0.032 |  | 2188 | 0.84 (0.77-0.92) | 0.0002 |  | 893 | 1.00 (0.85-1.17) | 0.98 | 0.20 | 38 |
| Fruit fibre | 2 | 1200 | 0.94 (0.88-1.01) | 0.086 |  | 2188 | 0.91 (0.86-0.96) | 0.0005 |  | 893 | 1.01 (0.91-1.11) | 0.91 | 0.19 | 40 |
| Vegetable fibre | 2 | 1200 | 0.92 (0.78-1.08) | 0.29 |  | 2188 | 0.87 (0.77-0.98) | 0.018 |  | 893 | 0.93 (0.74-1.17) | 0.51 | 0.81 | 0 |
| ^1^ Hazard ratios are adjusted for age (continuous), smoking status and number of cigarettes per day (never smoker, former smoker, current smoker <10 cigs/d, current smoker 10-19 cigs/d, current smoker 20+ cigs/d, unknown; where appropriate), history of diabetes (yes, no, unknown), prior hypertension (yes, no , unknown), prior hyperlipidaemia (yes, no, unknown), Cambridge physical activity index (inactive, moderately inactive, moderately active, active, unknown), employment status (employed or student, not employed or student, unknown), level of education completed (none or primary, secondary, vocational or university, unknown), current alcohol consumption (non-drinkers and sex-specific fifths of intake among drinkers), body mass index (<22.5, 22.5-24.9, 25.0-27.4, 27.5-29.9, ≥30.0 kg/m^2^, unknown), and calibrated intake of energy (continuous), and stratified by sex and EPIC centre. Unit sizes represent approximate differences in mean 24 hour recall intake between participants in the lowest and highest fifths of observed intake.  ^2^ Tests of trend were performed using the calibrated intake (continuous).  ^3^ Tests of heterogeneity of trend by age at recruitment were obtained assuming independence of risk by age at recruitment using a meta-analysis method. *I^2^* was estimated as (χ^2^ – degrees of freedom) / χ^2^ x 100.  ^4^ Unavailable for Naples, Heidelberg and Potsdam.  ^5^ Unavailable for Potsdam.  ^6^ Unavailable for Umea.  ^7^ Unavailable for Denmark and Norway. | | | | | | | | | | | | | | |

**Supplementary table 21**: Hazard ratios (95% confidence intervals) for **haemorrhagic stroke** per unit higher^1^ calibrated intake of major foods and fibre, **stratified** **by age at recruitment**, showing results from independent subset analyses in the EPIC study.

| Food | Unit sizes (g/day) | <55 years | | |  | 55-64 years | | |  | ≥65 years | | | Measures of heterogeneity^3^ | |
| --- | --- | --- | --- | --- | --- | --- | --- | --- | --- | --- | --- | --- | --- | --- |
|  |  | No. of cases | HR (95% CI) | P for trend^2^ |  | No. of cases | P-het | *I^2^* |  | No. of cases | HR (95% CI) | P for trend^2^ | P-het | *I^2^* (%) |
| Red and processed meat | 100 | 546 | 1.10 (0.72-1.66) | 0.67 |  | 637 | 1.07 (0.71-1.62) | 0.73 |  | 247 | 0.73 (0.35-1.52) | 0.40 | 0.62 | 0 |
| Red meat | 50 | 546 | 0.97 (0.71-1.33) | 0.86 |  | 637 | 1.01 (0.75-1.38) | 0.90 |  | 247 | 0.88 (0.52-1.47) | 0.61 | 0.88 | 0 |
| Processed meat | 50 | 546 | 1.12 (0.81-1.54) | 0.50 |  | 637 | 1.07 (0.79-1.46) | 0.66 |  | 247 | 1.02 (0.57-1.85) | 0.94 | 0.96 | 0 |
| Poultry meat | 20 | 546 | 0.91 (0.73-1.13) | 0.38 |  | 637 | 0.98 (0.80-1.21) | 0.86 |  | 247 | 0.92 (0.69-1.23) | 0.57 | 0.87 | 0 |
| White fish^4^ | 15 | 503 | 1.02 (0.88-1.18) | 0.82 |  | 586 | 1.01 (0.87-1.17) | 0.90 |  | 243 | 1.14 (0.90-1.45) | 0.27 | 0.66 | 0 |
| Fatty fish^5^ | 15 | 525 | 0.94 (0.79-1.13) | 0.50 |  | 608 | 0.94 (0.80-1.11) | 0.47 |  | 244 | 1.00 (0.80-1.26) | 0.98 | 0.89 | 0 |
| Milk | 200 | 546 | 1.11 (0.98-1.25) | 0.088 |  | 637 | 1.03 (0.92-1.15) | 0.60 |  | 247 | 1.09 (0.88-1.36) | 0.42 | 0.65 | 0 |
| Yogurt | 100 | 546 | 0.87 (0.71-1.07) | 0.19 |  | 637 | 0.96 (0.82-1.13) | 0.63 |  | 247 | 0.93 (0.68-1.28) | 0.67 | 0.76 | 0 |
| Cheese | 30 | 546 | 0.89 (0.69-1.14) | 0.36 |  | 637 | 0.88 (0.70-1.11) | 0.27 |  | 247 | 1.04 (0.65-1.65) | 0.88 | 0.82 | 0 |
| Eggs^6^ | 20 | 485 | 1.16 (0.92-1.47) | 0.21 |  | 578 | 1.28 (1.04-1.56) | 0.019 |  | 247 | 1.33 (0.99-1.80) | 0.062 | 0.76 | 0 |
| Cereals and cereal products | 200 | 546 | 1.19 (0.72-1.96) | 0.51 |  | 637 | 0.91 (0.57-1.43) | 0.67 |  | 247 | 1.05 (0.46-2.39) | 0.91 | 0.74 | 0 |
| Fruit and vegetables | 200 | 546 | 1.03 (0.87-1.21) | 0.74 |  | 637 | 0.93 (0.79-1.09) | 0.35 |  | 247 | 1.20 (0.91-1.57) | 0.20 | 0.27 | 25 |
| Fruit | 100 | 546 | 0.97 (0.87-1.07) | 0.51 |  | 637 | 0.97 (0.88-1.06) | 0.48 |  | 247 | 1.09 (0.93-1.29) | 0.28 | 0.39 | 0 |
| Vegetables | 100 | 546 | 1.16 (0.93-1.46) | 0.19 |  | 637 | 0.89 (0.71-1.10) | 0.28 |  | 247 | 1.20 (0.81-1.79) | 0.36 | 0.17 | 43 |
| Legumes^7^ | 20 | 423 | 1.07 (0.88-1.30) | 0.49 |  | 432 | 0.97 (0.77-1.21) | 0.77 |  | 241 | 1.17 (0.92-1.48) | 0.20 | 0.53 | 0 |
| Nuts and seeds^6^ | 10 | 485 | 0.93 (0.68-1.27) | 0.65 |  | 578 | 0.72 (0.49-1.05) | 0.087 |  | 247 | 1.12 (0.75-1.66) | 0.58 | 0.28 | 22 |
| Total dietary fibre | 10 | 546 | 1.08 (0.81-1.45) | 0.60 |  | 637 | 0.78 (0.60-1.02) | 0.070 |  | 247 | 1.52 (0.97-2.37) | 0.067 | 0.032 | 71 |
| Cereal fibre | 4 | 546 | 0.98 (0.82-1.17) | 0.83 |  | 637 | 0.88 (0.75-1.02) | 0.097 |  | 247 | 1.19 (0.89-1.61) | 0.24 | 0.18 | 41 |
| Fruit and vegetable fibre | 4 | 546 | 1.02 (0.86-1.21) | 0.83 |  | 637 | 0.92 (0.78-1.07) | 0.29 |  | 247 | 1.20 (0.91-1.59) | 0.20 | 0.25 | 29 |
| Fruit fibre | 2 | 546 | 0.96 (0.87-1.07) | 0.49 |  | 637 | 0.98 (0.89-1.08) | 0.66 |  | 247 | 1.11 (0.94-1.32) | 0.21 | 0.34 | 7 |
| Vegetable fibre | 2 | 546 | 1.10 (0.86-1.39) | 0.45 |  | 637 | 0.83 (0.67-1.04) | 0.11 |  | 247 | 1.10 (0.71-1.70) | 0.66 | 0.21 | 36 |
| ^1^ Hazard ratios are adjusted for age (continuous), smoking status and number of cigarettes per day (never smoker, former smoker, current smoker <10 cigs/d, current smoker 10-19 cigs/d, current smoker 20+ cigs/d, unknown; where appropriate), history of diabetes (yes, no, unknown), prior hypertension (yes, no , unknown), prior hyperlipidaemia (yes, no, unknown), Cambridge physical activity index (inactive, moderately inactive, moderately active, active, unknown), employment status (employed or student, not employed or student, unknown), level of education completed (none or primary, secondary, vocational or university, unknown), current alcohol consumption (non-drinkers and sex-specific fifths of intake among drinkers), body mass index (<22.5, 22.5-24.9, 25.0-27.4, 27.5-29.9, ≥30.0 kg/m^2^, unknown), and calibrated intake of energy (continuous), and stratified by sex and EPIC centre. Unit sizes represent approximate differences in mean 24 hour recall intake between participants in the lowest and highest fifths of observed intake.  ^2^ Tests of trend were performed using the calibrated intake (continuous).  ^3^ Tests of heterogeneity of trend by age at recruitment were obtained assuming independence of risk by age at recruitment using a meta-analysis method. *I^2^* was estimated as (χ^2^ – degrees of freedom) / χ^2^ x 100.  ^4^ Unavailable for Naples, Heidelberg and Potsdam.  ^5^ Unavailable for Potsdam.  ^6^ Unavailable for Umea.  ^7^ Unavailable for Denmark and Norway. | | | | | | | | | | | | | | |

**Supplementary table 22**: Hazard ratios (95% confidence intervals) for **ischaemic stroke** per unit higher^1^ calibrated intake of major foods and fibre, **stratified by sex**, showing results from independent subset analyses in the EPIC study.

| Food | Unit sizes (g/day) | Men | | |  | Women | | | Measures of heterogeneity^3^ | |
| --- | --- | --- | --- | --- | --- | --- | --- | --- | --- | --- |
|  |  | No. of cases | HR (95% CI) | P for trend^2^ |  | No. of cases | HR (95% CI) | P for trend^2^ | P-het | *I^2^* (%) |
|  |  |  |  |  |  |  |  |  |  |  |
| Red and processed meat | 100 | 2230 | 1.14 (0.95-1.36) | 0.17 |  | 2051 | 1.14 (0.88-1.49) | 0.32 | 0.98 | 0 |
| Red meat | 50 | 2230 | 1.08 (0.95-1.23) | 0.25 |  | 2051 | 1.30 (1.06-1.59) | 0.013 | 0.14 | 54 |
| Processed meat | 50 | 2230 | 1.10 (0.96-1.27) | 0.16 |  | 2051 | 0.91 (0.74-1.12) | 0.38 | 0.13 | 56 |
| Poultry meat | 20 | 2230 | 0.97 (0.88-1.07) | 0.56 |  | 2051 | 1.03 (0.89-1.19) | 0.73 | 0.54 | 0 |
| White fish^4^ | 15 | 1998 | 0.97 (0.92-1.03) | 0.32 |  | 1874 | 0.99 (0.90-1.10) | 0.90 | 0.71 | 0 |
| Fatty fish^5^ | 15 | 2108 | 0.99 (0.92-1.06) | 0.70 |  | 1951 | 1.03 (0.93-1.14) | 0.55 | 0.47 | 0 |
| Milk | 200 | 2230 | 0.95 (0.89-1.00) | 0.054 |  | 2051 | 0.97 (0.89-1.05) | 0.42 | 0.65 | 0 |
| Yogurt | 100 | 2230 | 0.92 (0.84-1.00) | 0.051 |  | 2051 | 0.90 (0.81-1.00) | 0.048 | 0.77 | 0 |
| Cheese | 30 | 2230 | 0.92 (0.82-1.03) | 0.16 |  | 2051 | 0.84 (0.72-0.98) | 0.024 | 0.33 | 0 |
| Eggs^6^ | 20 | 1923 | 1.07 (0.97-1.18) | 0.20 |  | 1849 | 1.01 (0.87-1.17) | 0.91 | 0.52 | 0 |
| Cereals and cereal products | 200 | 2230 | 0.92 (0.74-1.13) | 0.43 |  | 2051 | 1.07 (0.78-1.47) | 0.68 | 0.43 | 0 |
| Fruit and vegetables | 200 | 2230 | 0.83 (0.76-0.90) | <0.0001 |  | 2051 | 0.95 (0.86-1.05) | 0.31 | 0.040 | 76 |
| Fruit | 100 | 2230 | 0.91 (0.87-0.96) | 0.0001 |  | 2051 | 0.98 (0.92-1.04) | 0.43 | 0.089 | 65 |
| Vegetables | 100 | 2230 | 0.84 (0.74-0.95) | 0.005 |  | 2051 | 0.93 (0.81-1.05) | 0.23 | 0.28 | 14 |
| Legumes^7^ | 20 | 1771 | 0.97 (0.90-1.05) | 0.46 |  | 1740 | 0.88 (0.70-1.11) | 0.29 | 0.44 | 0 |
| Nuts and seeds^6^ | 10 | 1923 | 1.01 (0.86-1.19) | 0.90 |  | 1849 | 0.93 (0.76-1.13) | 0.46 | 0.51 | 0 |
| Total dietary fibre | 10 | 2230 | 0.74 (0.65-0.85) | <0.0001 |  | 2051 | 0.85 (0.72-1.01) | 0.071 | 0.22 | 35 |
| Cereal fibre | 4 | 2230 | 0.91 (0.84-0.99) | 0.026 |  | 2051 | 0.89 (0.79-1.00) | 0.057 | 0.73 | 0 |
| Fruit and vegetable fibre | 4 | 2230 | 0.83 (0.76-0.91) | 0.0001 |  | 2051 | 0.95 (0.86-1.04) | 0.25 | 0.060 | 72 |
| Fruit fibre | 2 | 2230 | 0.91 (0.86-0.95) | 0.0001 |  | 2051 | 0.98 (0.92-1.04) | 0.47 | 0.060 | 72 |
| Vegetable fibre | 2 | 2230 | 0.88 (0.78-1.00) | 0.044 |  | 2051 | 0.92 (0.81-1.04) | 0.19 | 0.61 | 0 |
| ^1^ Hazard ratios are adjusted for age (continuous), smoking status and number of cigarettes per day (never smoker, former smoker, current smoker <10 cigs/d, current smoker 10-19 cigs/d, current smoker 20+ cigs/d, unknown), history of diabetes, prior hypertension, prior hyperlipidaemia (each yes, no, unknown; where appropriate), Cambridge physical activity index (inactive, moderately inactive, moderately active, active, unknown), employment status (employed or student, not employed or student, unknown), level of education completed (none or primary, secondary, vocational or university, unknown), current alcohol consumption (non-drinkers and sex-specific fifths of intake among drinkers), body mass index (<22.5, 22.5-24.9, 25.0-27.4, 27.5-29.9, ≥30.0 kg/m^2^, unknown), and calibrated intake of energy (continuous), and stratified by sex and EPIC centre. Unit sizes represent approximate differences in mean 24 hour recall intake between participants in the lowest and highest fifths of observed intake.  ^2^ Tests of trend were performed using the calibrated intake (continuous).  ^3^ Tests of heterogeneity of trend by sex were obtained assuming independence of risk by sex using a meta-analysis method. *I^2^* was estimated as (χ^2^ – degrees of freedom) / χ^2^ x 100.  ^4^ Unavailable for Naples, Heidelberg and Potsdam.  ^5^ Unavailable for Potsdam.  ^6^ Unavailable for Umea.  ^7^ Unavailable for Denmark and Norway. | | | | | | | | | | |

**Supplementary table 23**: Hazard ratios (95% confidence intervals) for **haemorrhagic stroke** per unit higher^1^ calibrated intake of major foods and fibre, **stratified** **by sex**, showing results from independent subset analyses in the EPIC study.

| Food | Unit sizes (g/day) | Men | | |  | Women | | | Measures of heterogeneity^3^ | |
| --- | --- | --- | --- | --- | --- | --- | --- | --- | --- | --- |
|  |  | No. of cases | HR (95% CI) | P for trend^2^ |  | No. of cases | HR (95% CI) | P for trend^2^ | P-het | *I^2^* (%) |
|  |  |  |  |  |  |  |  |  |  |  |
| Red and processed meat | 100 | 585 | 0.82 (0.57-1.19) | 0.29 |  | 845 | 1.40 (0.93-2.11) | 0.11 | 0.056 | 73 |
| Red meat | 50 | 585 | 0.87 (0.67-1.12) | 0.27 |  | 845 | 1.20 (0.87-1.66) | 0.27 | 0.12 | 59 |
| Processed meat | 50 | 585 | 1.00 (0.75-1.33) | 0.99 |  | 845 | 1.23 (0.90-1.69) | 0.20 | 0.34 | 0 |
| Poultry meat | 20 | 585 | 0.89 (0.73-1.08) | 0.22 |  | 845 | 0.99 (0.81-1.20) | 0.90 | 0.45 | 0 |
| White fish^4^ | 15 | 527 | 1.01 (0.90-1.14) | 0.81 |  | 805 | 1.05 (0.89-1.24) | 0.59 | 0.76 | 0 |
| Fatty fish^5^ | 15 | 550 | 1.04 (0.90-1.21) | 0.55 |  | 827 | 0.88 (0.75-1.03) | 0.11 | 0.11 | 60 |
| Milk | 200 | 585 | 1.09 (0.98-1.20) | 0.11 |  | 845 | 1.05 (0.93-1.17) | 0.44 | 0.63 | 0 |
| Yogurt | 100 | 585 | 0.90 (0.76-1.07) | 0.23 |  | 845 | 0.94 (0.80-1.11) | 0.49 | 0.69 | 0 |
| Cheese | 30 | 585 | 0.92 (0.74-1.15) | 0.46 |  | 845 | 0.83 (0.66-1.05) | 0.12 | 0.53 | 0 |
| Eggs^6^ | 20 | 522 | 1.26 (1.05-1.51) | 0.013 |  | 788 | 1.25 (1.01-1.53) | 0.036 | 0.95 | 0 |
| Cereals and cereal products | 200 | 585 | 1.02 (0.68-1.54) | 0.91 |  | 845 | 1.16 (0.71-1.90) | 0.54 | 0.69 | 0 |
| Fruit and vegetables | 200 | 585 | 1.05 (0.90-1.21) | 0.56 |  | 845 | 0.94 (0.81-1.09) | 0.41 | 0.32 | 0 |
| Fruit | 100 | 585 | 1.01 (0.93-1.10) | 0.73 |  | 845 | 0.93 (0.85-1.03) | 0.15 | 0.19 | 42 |
| Vegetables | 100 | 585 | 1.06 (0.84-1.33) | 0.65 |  | 845 | 0.99 (0.82-1.20) | 0.90 | 0.67 | 0 |
| Legumes^7^ | 20 | 436 | 1.08 (0.95-1.22) | 0.26 |  | 660 | 1.00 (0.69-1.44) | 0.99 | 0.70 | 0 |
| Nuts and seeds^6^ | 10 | 522 | 0.82 (0.58-1.15) | 0.25 |  | 788 | 0.92 (0.71-1.21) | 0.57 | 0.58 | 0 |
| Total dietary fibre | 10 | 585 | 1.03 (0.80-1.33) | 0.80 |  | 845 | 0.93 (0.72-1.20) | 0.57 | 0.57 | 0 |
| Cereal fibre | 4 | 585 | 0.97 (0.84-1.12) | 0.65 |  | 845 | 0.95 (0.80-1.12) | 0.55 | 0.87 | 0 |
| Fruit and vegetable fibre | 4 | 585 | 1.02 (0.87-1.20) | 0.77 |  | 845 | 0.94 (0.82-1.09) | 0.45 | 0.47 | 0 |
| Fruit fibre | 2 | 585 | 1.02 (0.93-1.12) | 0.64 |  | 845 | 0.94 (0.86-1.04) | 0.23 | 0.23 | 31 |
| Vegetable fibre | 2 | 585 | 0.93 (0.72-1.19) | 0.56 |  | 845 | 0.95 (0.78-1.15) | 0.61 | 0.88 | 0 |
| ^1^ Hazard ratios are adjusted for age (continuous), smoking status and number of cigarettes per day (never smoker, former smoker, current smoker <10 cigs/d, current smoker 10-19 cigs/d, current smoker 20+ cigs/d, unknown), history of diabetes, prior hypertension, prior hyperlipidaemia (each yes, no, unknown; where appropriate), Cambridge physical activity index (inactive, moderately inactive, moderately active, active, unknown), employment status (employed or student, not employed or student, unknown), level of education completed (none or primary, secondary, vocational or university, unknown), current alcohol consumption (non-drinkers and sex-specific fifths of intake among drinkers), body mass index (<22.5, 22.5-24.9, 25.0-27.4, 27.5-29.9, ≥30.0 kg/m^2^, unknown), and calibrated intake of energy (continuous), and stratified by sex and EPIC centre. Unit sizes represent approximate differences in mean 24 hour recall intake between participants in the lowest and highest fifths of observed intake.  ^2^ Tests of trend were performed using the calibrated intake (continuous).  ^3^ Tests of heterogeneity of trend by sex were obtained assuming independence of risk by sex using a meta-analysis method. *I^2^* was estimated as (χ^2^ – degrees of freedom) / χ^2^ x 100.  ^4^ Unavailable for Naples, Heidelberg and Potsdam.  ^5^ Unavailable for Potsdam.  ^6^ Unavailable for Umea.  ^7^ Unavailable for Denmark and Norway. | | | | | | | | | | |

**Supplementary table 24**: Hazard ratios (95% confidence intervals) for **ischaemic stroke** per unit higher^1^ calibrated intake of major foods and fibre, **stratified by body mass index**, showing results from independent subset analyses in the EPIC study.

| Food | Unit sizes (g/day) | BMI <25 kg/m^2^ | | |  | BMI 25-29.9 kg/m^2^ | | |  | BMI ≥30 kg/m^2^ | | | Measures of heterogeneity^3^ | |
| --- | --- | --- | --- | --- | --- | --- | --- | --- | --- | --- | --- | --- | --- | --- |
|  |  | No. of cases | HR (95% CI) | P for trend^2^ |  | No. of cases | HR (95% CI) | P for trend^2^ |  | No. of cases | HR (95% CI) | P for trend^2^ | P-het | *I^2^* (%) |
| Red and processed meat | 100 | 1473 | 1.12 (0.86-1.46) | 0.41 |  | 1947 | 1.10 (0.88-1.37) | 0.41 |  | 846 | 1.26 (0.92-1.73) | 0.15 | 0.77 | 0 |
| Red meat | 50 | 1473 | 1.20 (0.99-1.46) | 0.067 |  | 1947 | 1.04 (0.89-1.23) | 0.60 |  | 846 | 1.23 (0.99-1.52) | 0.065 | 0.39 | 0 |
| Processed meat | 50 | 1473 | 0.97 (0.78-1.19) | 0.75 |  | 1947 | 1.06 (0.90-1.25) | 0.50 |  | 846 | 1.10 (0.86-1.41) | 0.45 | 0.70 | 0 |
| Poultry meat | 20 | 1473 | 1.05 (0.92-1.20) | 0.43 |  | 1947 | 0.93 (0.82-1.06) | 0.27 |  | 846 | 1.01 (0.84-1.21) | 0.93 | 0.40 | 0 |
| White fish^4^ | 15 | 1349 | 0.98 (0.88-1.08) | 0.65 |  | 1753 | 0.97 (0.90-1.04) | 0.37 |  | 756 | 1.00 (0.91-1.09) | 0.98 | 0.87 | 0 |
| Fatty fish^5^ | 15 | 1405 | 0.91 (0.82-1.02) | 0.10 |  | 1836 | 0.99 (0.91-1.08) | 0.90 |  | 804 | 1.13 (1.00-1.27) | 0.046 | 0.035 | 70 |
| Milk | 200 | 1473 | 0.95 (0.88-1.03) | 0.23 |  | 1947 | 0.93 (0.87-1.00) | 0.040 |  | 846 | 0.97 (0.87-1.07) | 0.51 | 0.82 | 0 |
| Yogurt | 100 | 1473 | 0.88 (0.79-0.98) | 0.025 |  | 1947 | 0.92 (0.83-1.01) | 0.070 |  | 846 | 0.98 (0.84-1.14) | 0.76 | 0.57 | 0 |
| Cheese | 30 | 1473 | 0.88 (0.75-1.03) | 0.10 |  | 1947 | 0.91 (0.79-1.04) | 0.17 |  | 846 | 0.87 (0.70-1.07) | 0.20 | 0.92 | 0 |
| Eggs^6^ | 20 | 1295 | 1.14 (0.99-1.32) | 0.070 |  | 1708 | 0.96 (0.84-1.08) | 0.48 |  | 758 | 1.11 (0.93-1.32) | 0.24 | 0.15 | 48 |
| Cereals and cereal products | 200 | 1473 | 0.98 (0.73-1.32) | 0.89 |  | 1947 | 0.97 (0.75-1.25) | 0.79 |  | 846 | 0.93 (0.61-1.42) | 0.74 | 0.98 | 0 |
| Fruit and vegetables | 200 | 1473 | 0.91 (0.81-1.03) | 0.13 |  | 1947 | 0.87 (0.79-0.95) | 0.002 |  | 846 | 0.86 (0.76-0.98) | 0.026 | 0.74 | 0 |
| Fruit | 100 | 1473 | 0.96 (0.89-1.03) | 0.26 |  | 1947 | 0.94 (0.89-0.99) | 0.016 |  | 846 | 0.90 (0.84-0.98) | 0.011 | 0.55 | 0 |
| Vegetables | 100 | 1473 | 0.87 (0.74-1.02) | 0.091 |  | 1947 | 0.85 (0.75-0.97) | 0.017 |  | 846 | 0.93 (0.77-1.11) | 0.40 | 0.77 | 0 |
| Legumes^7^ | 20 | 1165 | 1.02 (0.86-1.20) | 0.86 |  | 1610 | 0.96 (0.86-1.07) | 0.46 |  | 721 | 0.92 (0.80-1.06) | 0.27 | 0.70 | 0 |
| Nuts and seeds^6^ | 10 | 1295 | 1.06 (0.88-1.29) | 0.53 |  | 1708 | 0.97 (0.80-1.17) | 0.75 |  | 758 | 0.82 (0.59-1.13) | 0.22 | 0.38 | 0 |
| Total dietary fibre | 10 | 1473 | 0.87 (0.73-1.04) | 0.13 |  | 1947 | 0.72 (0.61-0.84) | <0.0001 |  | 846 | 0.77 (0.60-0.98) | 0.035 | 0.28 | 21 |
| Cereal fibre | 4 | 1473 | 0.93 (0.83-1.03) | 0.18 |  | 1947 | 0.87 (0.79-0.96) | 0.005 |  | 846 | 0.98 (0.83-1.15) | 0.78 | 0.41 | 0 |
| Fruit and vegetable fibre | 4 | 1473 | 0.91 (0.81-1.02) | 0.12 |  | 1947 | 0.86 (0.78-0.95) | 0.002 |  | 846 | 0.88 (0.76-1.00) | 0.054 | 0.75 | 0 |
| Fruit fibre | 2 | 1473 | 0.95 (0.89-1.03) | 0.20 |  | 1947 | 0.93 (0.88-0.98) | 0.013 |  | 846 | 0.91 (0.84-0.99) | 0.029 | 0.72 | 0 |
| Vegetable fibre | 2 | 1473 | 0.89 (0.76-1.05) | 0.17 |  | 1947 | 0.88 (0.77-1.00) | 0.055 |  | 846 | 0.93 (0.77-1.11) | 0.42 | 0.89 | 0 |
| ^1^ Hazard ratios are adjusted for age (continuous), smoking status and number of cigarettes per day (never smoker, former smoker, current smoker <10 cigs/d, current smoker 10-19 cigs/d, current smoker 20+ cigs/d, unknown; where appropriate), history of diabetes (yes, no, unknown), prior hypertension (yes, no , unknown), prior hyperlipidaemia (yes, no, unknown), Cambridge physical activity index (inactive, moderately inactive, moderately active, active, unknown), employment status (employed or student, not employed or student, unknown), level of education completed (none or primary, secondary, vocational or university, unknown), current alcohol consumption (non-drinkers and sex-specific fifths of intake among drinkers), body mass index (<22.5, 22.5-24.9, 25.0-27.4, 27.5-29.9, ≥30.0 kg/m^2^, unknown), and calibrated intake of energy (continuous), and stratified by sex and EPIC centre. Unit sizes represent approximate differences in mean 24 hour recall intake between participants in the lowest and highest fifths of observed intake.  ^2^ Tests of trend were performed using the calibrated intake (continuous).  ^3^ Tests of heterogeneity of trend by body mass index were obtained assuming independence of risk by body mass index using a meta-analysis method. *I^2^* was estimated as (χ^2^ – degrees of freedom) / χ^2^ x 100.  ^4^ Unavailable for Naples, Heidelberg and Potsdam.  ^5^ Unavailable for Potsdam.  ^6^ Unavailable for Umea.  ^7^ Unavailable for Denmark and Norway. | | | | | | | | | | | | | | |

**Supplementary table 25**: Hazard ratios (95% confidence intervals) for **haemorrhagic stroke** per unit higher^1^ calibrated intake of major foods and fibre, **stratified** **by body mass index**, showing results from independent subset analyses in the EPIC study.

| Food | Unit sizes (g/day) | BMI <25 kg/m^2^ | | |  | BMI 25-29.9 kg/m^2^ | | |  | BMI ≥30 kg/m^2^ | | | Measures of heterogeneity^3^ | |
| --- | --- | --- | --- | --- | --- | --- | --- | --- | --- | --- | --- | --- | --- | --- |
|  |  | No. of cases | HR (95% CI) | P for trend^2^ |  | No. of cases | HR (95% CI) | P for trend^2^ |  | No. of cases | HR (95% CI) | P for trend^2^ | P-het | *I^2^* (%) |
| Red and processed meat | 100 | 602 | 1.09 (0.70-1.69) | 0.70 |  | 581 | 0.91 (0.59-1.39) | 0.65 |  | 242 | 1.10 (0.60-1.99) | 0.76 | 0.81 | 0 |
| Red meat | 50 | 602 | 1.24 (0.89-1.72) | 0.20 |  | 581 | 0.80 (0.59-1.09) | 0.15 |  | 242 | 0.98 (0.63-1.51) | 0.92 | 0.16 | 45 |
| Processed meat | 50 | 602 | 0.94 (0.66-1.34) | 0.74 |  | 581 | 1.21 (0.88-1.65) | 0.24 |  | 242 | 1.03 (0.65-1.62) | 0.90 | 0.58 | 0 |
| Poultry meat | 20 | 602 | 0.93 (0.75-1.15) | 0.49 |  | 581 | 0.86 (0.70-1.07) | 0.18 |  | 242 | 1.19 (0.87-1.63) | 0.29 | 0.26 | 26 |
| White fish^4^ | 15 | 570 | 0.99 (0.82-1.20) | 0.93 |  | 539 | 1.03 (0.90-1.18) | 0.67 |  | 218 | 1.08 (0.90-1.29) | 0.41 | 0.82 | 0 |
| Fatty fish^5^ | 15 | 587 | 0.93 (0.78-1.11) | 0.42 |  | 557 | 1.02 (0.87-1.20) | 0.80 |  | 228 | 0.94 (0.73-1.23) | 0.67 | 0.73 | 0 |
| Milk | 200 | 602 | 0.99 (0.87-1.12) | 0.84 |  | 581 | 1.15 (1.03-1.29) | 0.012 |  | 242 | 1.03 (0.86-1.23) | 0.73 | 0.17 | 43 |
| Yogurt | 100 | 602 | 0.88 (0.73-1.06) | 0.17 |  | 581 | 0.99 (0.83-1.19) | 0.93 |  | 242 | 0.93 (0.69-1.26) | 0.63 | 0.66 | 0 |
| Cheese | 30 | 602 | 0.92 (0.72-1.18) | 0.50 |  | 581 | 0.80 (0.62-1.03) | 0.082 |  | 242 | 1.07 (0.74-1.53) | 0.72 | 0.42 | 0 |
| Eggs^6^ | 20 | 552 | 1.23 (0.99-1.54) | 0.061 |  | 530 | 1.31 (1.07-1.61) | 0.010 |  | 224 | 1.15 (0.82-1.61) | 0.43 | 0.79 | 0 |
| Cereals and cereal products | 200 | 602 | 0.95 (0.58-1.56) | 0.84 |  | 581 | 0.92 (0.57-1.51) | 0.75 |  | 242 | 1.99 (0.97-4.08) | 0.059 | 0.18 | 43 |
| Fruit and vegetables | 200 | 602 | 1.08 (0.91-1.27) | 0.39 |  | 581 | 0.98 (0.84-1.15) | 0.82 |  | 242 | 0.92 (0.71-1.18) | 0.49 | 0.53 | 0 |
| Fruit | 100 | 602 | 1.05 (0.94-1.16) | 0.40 |  | 581 | 0.95 (0.86-1.04) | 0.27 |  | 242 | 0.95 (0.82-1.10) | 0.49 | 0.34 | 7 |
| Vegetables | 100 | 602 | 1.00 (0.79-1.27) | 0.98 |  | 581 | 1.10 (0.88-1.38) | 0.40 |  | 242 | 0.98 (0.69-1.38) | 0.89 | 0.79 | 0 |
| Legumes^7^ | 20 | 447 | 1.24 (1.07-1.42) | 0.003 |  | 448 | 0.99 (0.82-1.20) | 0.91 |  | 196 | 0.93 (0.69-1.27) | 0.66 | 0.088 | 59 |
| Nuts and seeds^6^ | 10 | 552 | 1.01 (0.76-1.34) | 0.94 |  | 530 | 0.91 (0.64-1.30) | 0.61 |  | 224 | 0.53 (0.27-1.06) | 0.073 | 0.24 | 29 |
| Total dietary fibre | 10 | 602 | 1.13 (0.86-1.49) | 0.37 |  | 581 | 0.90 (0.68-1.20) | 0.49 |  | 242 | 0.91 (0.58-1.43) | 0.69 | 0.48 | 0 |
| Cereal fibre | 4 | 602 | 0.92 (0.78-1.09) | 0.33 |  | 581 | 0.94 (0.79-1.12) | 0.49 |  | 242 | 1.11 (0.85-1.46) | 0.43 | 0.48 | 0 |
| Fruit and vegetable fibre | 4 | 602 | 1.06 (0.90-1.26) | 0.48 |  | 581 | 0.98 (0.83-1.16) | 0.84 |  | 242 | 0.89 (0.68-1.15) | 0.37 | 0.51 | 0 |
| Fruit fibre | 2 | 602 | 1.04 (0.93-1.15) | 0.49 |  | 581 | 0.96 (0.87-1.06) | 0.41 |  | 242 | 0.97 (0.83-1.13) | 0.72 | 0.55 | 0 |
| Vegetable fibre | 2 | 602 | 1.00 (0.79-1.27) | 0.98 |  | 581 | 1.00 (0.79-1.27) | 0.99 |  | 242 | 0.81 (0.56-1.17) | 0.26 | 0.58 | 0 |
| ^1^ Hazard ratios are adjusted for age (continuous), smoking status and number of cigarettes per day (never smoker, former smoker, current smoker <10 cigs/d, current smoker 10-19 cigs/d, current smoker 20+ cigs/d, unknown; where appropriate), history of diabetes (yes, no, unknown), prior hypertension (yes, no , unknown), prior hyperlipidaemia (yes, no, unknown), Cambridge physical activity index (inactive, moderately inactive, moderately active, active, unknown), employment status (employed or student, not employed or student, unknown), level of education completed (none or primary, secondary, vocational or university, unknown), current alcohol consumption (non-drinkers and sex-specific fifths of intake among drinkers), body mass index (<22.5, 22.5-24.9, 25.0-27.4, 27.5-29.9, ≥30.0 kg/m^2^, unknown), and calibrated intake of energy (continuous), and stratified by sex and EPIC centre. Unit sizes represent approximate differences in mean 24 hour recall intake between participants in the lowest and highest fifths of observed intake.  ^2^ Tests of trend were performed using the calibrated intake (continuous).  ^3^ Tests of heterogeneity of trend by body mass index were obtained assuming independence of risk by body mass index using a meta-analysis method. *I^2^* was estimated as (χ^2^ – degrees of freedom) / χ^2^ x 100.  ^4^ Unavailable for Naples, Heidelberg and Potsdam.  ^5^ Unavailable for Potsdam.  ^6^ Unavailable for Umea.  ^7^ Unavailable for Denmark and Norway. | | | | | | | | | | | | | | |

**Supplementary table 26**: Hazard ratios (95% confidence intervals) for **ischaemic stroke** per unit higher^1^ calibrated intake of major foods and fibre, **stratified** **by prior disease status**, showing results from independent subset analyses in the EPIC study.

| Food | Unit sizes (g/day) | Participants with no disease history^2^ | | |  | Participants with a history of disease^2^ | | | Measures of heterogeneity^4^ | |
| --- | --- | --- | --- | --- | --- | --- | --- | --- | --- | --- |
|  |  | No. of cases | HR (95% CI) | P for trend^3^ |  | No. of cases | HR (95% CI) | P for trend^3^ | P-het | *I^2^* (%) |
|  |  |  |  |  |  |  |  |  |  |  |
| Red and processed meat | 100 | 1301 | 1.17 (0.89-1.53) | 0.26 |  | 1825 | 1.04 (0.83-1.30) | 0.73 | 0.52 | 0 |
| Red meat | 50 | 1301 | 1.15 (0.94-1.39) | 0.17 |  | 1825 | 1.09 (0.93-1.28) | 0.29 | 0.70 | 0 |
| Processed meat | 50 | 1301 | 1.04 (0.83-1.30) | 0.72 |  | 1825 | 1.03 (0.86-1.23) | 0.76 | 0.92 | 0 |
| Poultry meat | 20 | 1301 | 1.08 (0.95-1.24) | 0.23 |  | 1825 | 0.95 (0.84-1.07) | 0.36 | 0.13 | 55 |
| White fish^5^ | 15 | 1184 | 1.04 (0.95-1.15) | 0.41 |  | 1533 | 0.96 (0.89-1.02) | 0.19 | 0.15 | 51 |
| Fatty fish^6^ | 15 | 1237 | 0.99 (0.88-1.11) | 0.89 |  | 1667 | 1.00 (0.92-1.09) | 0.98 | 0.90 | 0 |
| Milk | 200 | 1301 | 0.91 (0.83-0.99) | 0.023 |  | 1825 | 1.00 (0.93-1.08) | 0.93 | 0.072 | 69 |
| Yogurt | 100 | 1301 | 0.91 (0.81-1.03) | 0.15 |  | 1825 | 0.91 (0.82-1.01) | 0.082 | 0.99 | 0 |
| Cheese | 30 | 1301 | 0.78 (0.66-0.93) | 0.007 |  | 1825 | 0.99 (0.86-1.15) | 0.94 | 0.041 | 76 |
| Eggs^7^ | 20 | 990 | 0.88 (0.75-1.04) | 0.14 |  | 1632 | 1.11 (0.97-1.26) | 0.13 | 0.034 | 78 |
| Cereals and cereal products | 200 | 1301 | 0.93 (0.67-1.31) | 0.69 |  | 1825 | 0.93 (0.71-1.21) | 0.58 | 0.97 | 0 |
| Fruit and vegetables | 200 | 1301 | 0.94 (0.84-1.04) | 0.24 |  | 1825 | 0.85 (0.77-0.93) | 0.0005 | 0.16 | 50 |
| Fruit | 100 | 1301 | 0.98 (0.92-1.04) | 0.49 |  | 1825 | 0.91 (0.87-0.97) | 0.002 | 0.11 | 61 |
| Vegetables | 100 | 1301 | 0.87 (0.74-1.03) | 0.098 |  | 1825 | 0.85 (0.75-0.97) | 0.016 | 0.83 | 0 |
| Legumes^8^ | 20 | 1041 | 0.95 (0.85-1.07) | 0.38 |  | 1574 | 0.94 (0.85-1.05) | 0.27 | 0.91 | 0 |
| Nuts and seeds^7^ | 10 | 990 | 1.03 (0.85-1.24) | 0.80 |  | 1632 | 0.96 (0.80-1.15) | 0.63 | 0.60 | 0 |
| Total dietary fibre | 10 | 1301 | 0.78 (0.65-0.94) | 0.010 |  | 1825 | 0.77 (0.66-0.91) | 0.002 | 0.92 | 0 |
| Cereal fibre | 4 | 1301 | 0.88 (0.78-0.98) | 0.024 |  | 1825 | 0.93 (0.84-1.03) | 0.15 | 0.49 | 0 |
| Fruit and vegetable fibre | 4 | 1301 | 0.92 (0.82-1.03) | 0.16 |  | 1825 | 0.86 (0.78-0.95) | 0.003 | 0.38 | 0 |
| Fruit fibre | 2 | 1301 | 0.97 (0.91-1.04) | 0.44 |  | 1825 | 0.92 (0.87-0.97) | 0.004 | 0.18 | 44 |
| Vegetable fibre | 2 | 1301 | 0.88 (0.75-1.04) | 0.13 |  | 1825 | 0.89 (0.79-1.02) | 0.087 | 0.88 | 0 |
| ^1^ Hazard ratios are adjusted for age (continuous), smoking status and number of cigarettes per day (never smoker, former smoker, current smoker <10 cigs/d, current smoker 10-19 cigs/d, current smoker 20+ cigs/d, unknown), history of diabetes, prior hypertension, prior hyperlipidaemia (each yes, no, unknown; where appropriate), Cambridge physical activity index (inactive, moderately inactive, moderately active, active, unknown), employment status (employed or student, not employed or student, unknown), level of education completed (none or primary, secondary, vocational or university, unknown), current alcohol consumption (non-drinkers and sex-specific fifths of intake among drinkers), body mass index (<22.5, 22.5-24.9, 25.0-27.4, 27.5-29.9, ≥30.0 kg/m^2^, unknown), and calibrated intake of energy (continuous), and stratified by sex and EPIC centre. Unit sizes represent approximate differences in mean 24 hour recall intake between participants in the lowest and highest fifths of observed intake.  ^2^ History of diabetes, previous hypertension, or previous hyperlipidaemia.  ^2^ Tests of trend were performed using the calibrated intake (continuous).  ^3^ Tests of heterogeneity of trend by prior disease status were obtained assuming independence of risk by prior disease status using a meta-analysis method. *I^2^* was estimated as (χ^2^ – degrees of freedom) / χ^2^ x 100.  ^4^ Unavailable for Naples, Heidelberg and Potsdam.  ^5^ Unavailable for Potsdam.  ^6^ Unavailable for Umea.  ^7^ Unavailable for Denmark and Norway. | | | | | | | | | | |

**Supplementary table 27**: Hazard ratios (95% confidence intervals) for **haemorrhagic stroke** per unit higher^1^ calibrated intake of major foods and fibre, **stratified by prior disease status**, showing results from independent subset analyses in the EPIC study.

| Food | Unit sizes (g/day) | Participants with no disease history^2^ | | |  | Participants with a history of disease^2^ | | | Measures of heterogeneity^4^ | |
| --- | --- | --- | --- | --- | --- | --- | --- | --- | --- | --- |
|  |  | No. of cases | HR (95% CI) | P for trend^3^ |  | No. of cases | HR (95% CI) | P for trend^3^ | P-het | *I^2^* (%) |
|  |  |  |  |  |  |  |  |  |  |  |
| Red and processed meat | 100 | 548 | 0.93 (0.60-1.46) | 0.76 |  | 520 | 0.92 (0.59-1.43) | 0.71 | 0.97 | 0 |
| Red meat | 50 | 548 | 0.86 (0.62-1.18) | 0.34 |  | 520 | 0.87 (0.62-1.21) | 0.40 | 0.96 | 0 |
| Processed meat | 50 | 548 | 1.01 (0.70-1.45) | 0.97 |  | 520 | 1.14 (0.81-1.60) | 0.45 | 0.63 | 0 |
| Poultry meat | 20 | 548 | 0.89 (0.73-1.10) | 0.29 |  | 520 | 0.94 (0.76-1.18) | 0.61 | 0.72 | 0 |
| White fish^5^ | 15 | 519 | 1.02 (0.87-1.18) | 0.85 |  | 452 | 1.04 (0.88-1.21) | 0.67 | 0.86 | 0 |
| Fatty fish^6^ | 15 | 534 | 0.95 (0.79-1.14) | 0.56 |  | 482 | 0.91 (0.75-1.10) | 0.31 | 0.73 | 0 |
| Milk | 200 | 548 | 1.00 (0.88-1.14) | 0.98 |  | 520 | 1.09 (0.95-1.24) | 0.22 | 0.38 | 0 |
| Yogurt | 100 | 548 | 0.94 (0.77-1.15) | 0.57 |  | 520 | 0.91 (0.74-1.11) | 0.35 | 0.78 | 0 |
| Cheese | 30 | 548 | 0.93 (0.72-1.22) | 0.62 |  | 520 | 0.92 (0.70-1.20) | 0.52 | 0.91 | 0 |
| Eggs^7^ | 20 | 480 | 1.22 (0.99-1.51) | 0.064 |  | 468 | 1.17 (0.90-1.53) | 0.23 | 0.81 | 0 |
| Cereals and cereal products | 200 | 548 | 1.09 (0.64-1.86) | 0.74 |  | 520 | 0.90 (0.53-1.51) | 0.69 | 0.60 | 0 |
| Fruit and vegetables | 200 | 548 | 1.10 (0.95-1.29) | 0.21 |  | 520 | 0.97 (0.81-1.15) | 0.72 | 0.27 | 17 |
| Fruit | 100 | 548 | 0.99 (0.90-1.09) | 0.84 |  | 520 | 1.03 (0.93-1.14) | 0.57 | 0.58 | 0 |
| Vegetables | 100 | 548 | 1.30 (1.04-1.62) | 0.021 |  | 520 | 0.85 (0.66-1.10) | 0.22 | 0.015 | 83 |
| Legumes^8^ | 20 | 445 | 1.05 (0.90-1.23) | 0.54 |  | 420 | 1.06 (0.85-1.34) | 0.59 | 0.92 | 0 |
| Nuts and seeds^7^ | 10 | 480 | 1.01 (0.77-1.33) | 0.94 |  | 468 | 0.78 (0.53-1.14) | 0.20 | 0.28 | 16 |
| Total dietary fibre | 10 | 548 | 1.11 (0.84-1.46) | 0.48 |  | 520 | 1.07 (0.79-1.44) | 0.67 | 0.87 | 0 |
| Cereal fibre | 4 | 548 | 0.95 (0.79-1.13) | 0.54 |  | 520 | 1.05 (0.88-1.26) | 0.60 | 0.42 | 0 |
| Fruit and vegetable fibre | 4 | 548 | 1.12 (0.95-1.32) | 0.17 |  | 520 | 0.94 (0.78-1.13) | 0.50 | 0.16 | 50 |
| Fruit fibre | 2 | 548 | 1.00 (0.91-1.11) | 0.95 |  | 520 | 1.04 (0.94-1.16) | 0.43 | 0.59 | 0 |
| Vegetable fibre | 2 | 548 | 1.24 (0.98-1.56) | 0.072 |  | 520 | 0.77 (0.59-1.00) | 0.049 | 0.008 | 86 |
| ^1^ Hazard ratios are adjusted for age (continuous), smoking status and number of cigarettes per day (never smoker, former smoker, current smoker <10 cigs/d, current smoker 10-19 cigs/d, current smoker 20+ cigs/d, unknown), history of diabetes, prior hypertension, prior hyperlipidaemia (each yes, no, unknown; where appropriate), Cambridge physical activity index (inactive, moderately inactive, moderately active, active, unknown), employment status (employed or student, not employed or student, unknown), level of education completed (none or primary, secondary, vocational or university, unknown), current alcohol consumption (non-drinkers and sex-specific fifths of intake among drinkers), body mass index (<22.5, 22.5-24.9, 25.0-27.4, 27.5-29.9, ≥30.0 kg/m^2^, unknown), and calibrated intake of energy (continuous), and stratified by sex and EPIC centre. Unit sizes represent approximate differences in mean 24 hour recall intake between participants in the lowest and highest fifths of observed intake.  ^2^ History of diabetes, previous hypertension, or previous hyperlipidaemia.  ^2^ Tests of trend were performed using the calibrated intake (continuous).  ^3^ Tests of heterogeneity of trend by prior disease status were obtained assuming independence of risk by prior disease status using a meta-analysis method. *I^2^* was estimated as (χ^2^ – degrees of freedom) / χ^2^ x 100.  ^4^ Unavailable for Naples, Heidelberg and Potsdam.  ^5^ Unavailable for Potsdam.  ^6^ Unavailable for Umea.  ^7^ Unavailable for Denmark and Norway. | | | | | | | | | | |

**Supplementary table 28**: Hazard ratios (95% confidence intervals) for **ischaemic stroke** per unit higher^1^ calibrated intake of major foods and fibre, **stratified by smoking status**, showing results from independent subset analyses in the EPIC study.

| Food | Unit sizes (g/day) | Never smoker | | |  | Former smoker | | |  | Current smoker | | | Measures of heterogeneity^3^ | |
| --- | --- | --- | --- | --- | --- | --- | --- | --- | --- | --- | --- | --- | --- | --- |
|  |  | No. of cases | HR (95% CI) | P for trend^2^ |  | No. of cases | HR (95% CI) | P for trend^2^ |  | No. of cases | HR (95% CI) | P for trend^2^ | P-het | *I^2^* (%) |
| Red and processed meat | 100 | 1623 | 1.11 (0.85-1.45) | 0.46 |  | 1215 | 1.25 (0.96-1.64) | 0.10 |  | 1411 | 1.10 (0.86-1.41) | 0.46 | 0.75 | 0 |
| Red meat | 50 | 1623 | 1.16 (0.96-1.41) | 0.14 |  | 1215 | 1.10 (0.90-1.35) | 0.36 |  | 1411 | 1.17 (0.98-1.39) | 0.084 | 0.90 | 0 |
| Processed meat | 50 | 1623 | 0.97 (0.79-1.21) | 0.81 |  | 1215 | 1.09 (0.88-1.34) | 0.44 |  | 1411 | 1.06 (0.89-1.28) | 0.50 | 0.75 | 0 |
| Poultry meat | 20 | 1623 | 1.04 (0.90-1.19) | 0.62 |  | 1215 | 0.96 (0.83-1.11) | 0.58 |  | 1411 | 0.96 (0.84-1.11) | 0.62 | 0.70 | 0 |
| White fish^4^ | 15 | 1470 | 0.99 (0.91-1.07) | 0.76 |  | 1078 | 0.95 (0.86-1.04) | 0.23 |  | 1292 | 1.00 (0.92-1.08) | 0.96 | 0.66 | 0 |
| Fatty fish^5^ | 15 | 1534 | 1.03 (0.93-1.13) | 0.56 |  | 1135 | 0.97 (0.88-1.08) | 0.61 |  | 1358 | 0.99 (0.89-1.09) | 0.78 | 0.72 | 0 |
| Milk | 200 | 1623 | 0.93 (0.86-1.01) | 0.096 |  | 1215 | 1.00 (0.92-1.09) | 0.97 |  | 1411 | 0.93 (0.87-1.00) | 0.054 | 0.40 | 0 |
| Yogurt | 100 | 1623 | 0.94 (0.85-1.05) | 0.30 |  | 1215 | 0.86 (0.76-0.97) | 0.014 |  | 1411 | 0.89 (0.80-1.00) | 0.053 | 0.50 | 0 |
| Cheese | 30 | 1623 | 0.87 (0.74-1.03) | 0.11 |  | 1215 | 0.80 (0.67-0.95) | 0.011 |  | 1411 | 0.96 (0.83-1.11) | 0.56 | 0.28 | 21 |
| Eggs^6^ | 20 | 1389 | 1.13 (0.97-1.30) | 0.11 |  | 1089 | 1.02 (0.87-1.19) | 0.83 |  | 1275 | 1.03 (0.90-1.17) | 0.71 | 0.57 | 0 |
| Cereals and cereal products | 200 | 1623 | 0.98 (0.72-1.33) | 0.90 |  | 1215 | 0.95 (0.70-1.30) | 0.76 |  | 1411 | 0.94 (0.69-1.26) | 0.66 | 0.98 | 0 |
| Fruit and vegetables | 200 | 1623 | 0.97 (0.88-1.07) | 0.57 |  | 1215 | 0.80 (0.71-0.91) | 0.0004 |  | 1411 | 0.84 (0.75-0.94) | 0.002 | 0.038 | 70 |
| Fruit | 100 | 1623 | 0.97 (0.91-1.03) | 0.34 |  | 1215 | 0.90 (0.83-0.97) | 0.004 |  | 1411 | 0.91 (0.86-0.98) | 0.007 | 0.22 | 35 |
| Vegetables | 100 | 1623 | 1.01 (0.87-1.16) | 0.93 |  | 1215 | 0.78 (0.66-0.92) | 0.003 |  | 1411 | 0.83 (0.71-0.97) | 0.022 | 0.052 | 66 |
| Legumes^7^ | 20 | 1442 | 0.89 (0.78-1.02) | 0.10 |  | 996 | 1.07 (0.94-1.22) | 0.33 |  | 1044 | 0.95 (0.84-1.07) | 0.41 | 0.17 | 44 |
| Nuts and seeds^6^ | 10 | 1389 | 0.94 (0.76-1.17) | 0.60 |  | 1089 | 1.09 (0.88-1.35) | 0.43 |  | 1275 | 0.86 (0.69-1.08) | 0.20 | 0.33 | 9 |
| Total dietary fibre | 10 | 1623 | 0.85 (0.70-1.02) | 0.075 |  | 1215 | 0.71 (0.58-0.86) | 0.0006 |  | 1411 | 0.76 (0.63-0.91) | 0.003 | 0.42 | 0 |
| Cereal fibre | 4 | 1623 | 0.88 (0.78-0.99) | 0.033 |  | 1215 | 0.88 (0.78-0.99) | 0.033 |  | 1411 | 0.95 (0.85-1.06) | 0.37 | 0.52 | 0 |
| Fruit and vegetable fibre | 4 | 1623 | 0.98 (0.89-1.09) | 0.74 |  | 1215 | 0.81 (0.71-0.91) | 0.0005 |  | 1411 | 0.83 (0.74-0.94) | 0.002 | 0.027 | 72 |
| Fruit fibre | 2 | 1623 | 0.97 (0.91-1.04) | 0.38 |  | 1215 | 0.91 (0.84-0.97) | 0.008 |  | 1411 | 0.91 (0.85-0.97) | 0.005 | 0.24 | 30 |
| Vegetable fibre | 2 | 1623 | 1.03 (0.90-1.18) | 0.69 |  | 1215 | 0.78 (0.66-0.92) | 0.003 |  | 1411 | 0.86 (0.74-1.01) | 0.061 | 0.033 | 71 |
| ^1^ Hazard ratios are adjusted for age (continuous), smoking status and number of cigarettes per day (never smoker, former smoker, current smoker <10 cigs/d, current smoker 10-19 cigs/d, current smoker 20+ cigs/d, unknown; where appropriate), history of diabetes (yes, no, unknown), prior hypertension (yes, no , unknown), prior hyperlipidaemia (yes, no, unknown), Cambridge physical activity index (inactive, moderately inactive, moderately active, active, unknown), employment status (employed or student, not employed or student, unknown), level of education completed (none or primary, secondary, vocational or university, unknown), current alcohol consumption (non-drinkers and sex-specific fifths of intake among drinkers), body mass index (<22.5, 22.5-24.9, 25.0-27.4, 27.5-29.9, ≥30.0 kg/m^2^, unknown), and calibrated intake of energy (continuous), and stratified by sex and EPIC centre. Unit sizes represent approximate differences in mean 24 hour recall intake between participants in the lowest and highest fifths of observed intake.  ^2^ Tests of trend were performed using the calibrated intake (continuous).  ^3^ Tests of heterogeneity of trend by smoking status were obtained assuming independence of risk by smoking status using a meta-analysis method. *I^2^* was estimated as (χ^2^ – degrees of freedom) / χ^2^ x 100.  ^4^ Unavailable for Naples, Heidelberg and Potsdam.  ^5^ Unavailable for Potsdam.  ^6^ Unavailable for Umea.  ^7^ Unavailable for Denmark and Norway. | | | | | | | | | | | | | | |

**Supplementary table 29**: Hazard ratios (95% confidence intervals) for **haemorrhagic stroke** per unit higher^1^ calibrated intake of major foods and fibre, **stratified** **by smoking status**, showing results from independent subset analyses in the EPIC study.

| Food | Unit sizes (g/day) | Never smoker | | |  | Former smoker | | |  | Current smoker | | | Measures of heterogeneity^3^ | |
| --- | --- | --- | --- | --- | --- | --- | --- | --- | --- | --- | --- | --- | --- | --- |
|  |  | No. of cases | HR (95% CI) | P for trend^2^ |  | No. of cases | HR (95% CI) | P for trend^2^ |  | No. of cases | HR (95% CI) | P for trend^2^ | P-het | *I^2^* (%) |
| Red and processed meat | 100 | 540 | 0.97 (0.60-1.56) | 0.89 |  | 413 | 0.82 (0.51-1.33) | 0.42 |  | 467 | 1.32 (0.85-2.05) | 0.21 | 0.34 | 8 |
| Red meat | 50 | 540 | 0.96 (0.67-1.37) | 0.81 |  | 413 | 0.87 (0.61-1.24) | 0.44 |  | 467 | 1.10 (0.79-1.52) | 0.57 | 0.63 | 0 |
| Processed meat | 50 | 540 | 1.16 (0.80-1.69) | 0.44 |  | 413 | 0.91 (0.62-1.34) | 0.64 |  | 467 | 1.20 (0.86-1.67) | 0.28 | 0.53 | 0 |
| Poultry meat | 20 | 540 | 0.95 (0.75-1.19) | 0.64 |  | 413 | 1.00 (0.80-1.24) | 0.97 |  | 467 | 0.83 (0.64-1.09) | 0.18 | 0.59 | 0 |
| White fish^4^ | 15 | 500 | 1.08 (0.92-1.27) | 0.32 |  | 381 | 1.02 (0.86-1.20) | 0.85 |  | 442 | 0.98 (0.83-1.16) | 0.82 | 0.69 | 0 |
| Fatty fish^5^ | 15 | 516 | 0.95 (0.79-1.14) | 0.57 |  | 392 | 1.06 (0.89-1.26) | 0.52 |  | 459 | 0.88 (0.72-1.07) | 0.19 | 0.36 | 1 |
| Milk | 200 | 540 | 1.18 (1.04-1.35) | 0.013 |  | 413 | 1.11 (0.97-1.28) | 0.12 |  | 467 | 0.96 (0.84-1.09) | 0.52 | 0.073 | 62 |
| Yogurt | 100 | 540 | 0.95 (0.78-1.15) | 0.59 |  | 413 | 1.01 (0.82-1.25) | 0.89 |  | 467 | 0.83 (0.67-1.03) | 0.092 | 0.42 | 0 |
| Cheese | 30 | 540 | 0.76 (0.56-1.01) | 0.060 |  | 413 | 0.86 (0.64-1.14) | 0.29 |  | 467 | 1.04 (0.80-1.34) | 0.78 | 0.26 | 25 |
| Eggs^6^ | 20 | 482 | 1.13 (0.87-1.49) | 0.36 |  | 393 | 1.41 (1.13-1.76) | 0.002 |  | 427 | 1.24 (0.98-1.57) | 0.071 | 0.45 | 0 |
| Cereals and cereal products | 200 | 540 | 0.87 (0.51-1.48) | 0.61 |  | 413 | 1.08 (0.62-1.88) | 0.79 |  | 467 | 1.16 (0.67-2.01) | 0.60 | 0.75 | 0 |
| Fruit and vegetables | 200 | 540 | 0.93 (0.78-1.11) | 0.41 |  | 413 | 1.11 (0.93-1.33) | 0.23 |  | 467 | 0.97 (0.80-1.18) | 0.79 | 0.33 | 9 |
| Fruit | 100 | 540 | 0.96 (0.86-1.07) | 0.42 |  | 413 | 1.00 (0.89-1.11) | 0.96 |  | 467 | 1.00 (0.89-1.12) | 0.97 | 0.83 | 0 |
| Vegetables | 100 | 540 | 0.86 (0.66-1.11) | 0.23 |  | 413 | 1.34 (1.05-1.71) | 0.020 |  | 467 | 0.98 (0.75-1.27) | 0.85 | 0.039 | 69 |
| Legumes^7^ | 20 | 461 | 1.12 (0.90-1.39) | 0.31 |  | 309 | 1.02 (0.83-1.26) | 0.84 |  | 318 | 1.08 (0.85-1.36) | 0.52 | 0.84 | 0 |
| Nuts and seeds^6^ | 10 | 482 | 1.29 (0.98-1.68) | 0.068 |  | 393 | 0.62 (0.40-0.96) | 0.032 |  | 427 | 0.70 (0.46-1.07) | 0.10 | 0.005 | 0 |
| Total dietary fibre | 10 | 540 | 0.91 (0.67-1.24) | 0.55 |  | 413 | 0.99 (0.73-1.35) | 0.95 |  | 467 | 1.10 (0.81-1.51) | 0.53 | 0.69 | 0 |
| Cereal fibre | 4 | 540 | 0.94 (0.77-1.14) | 0.53 |  | 413 | 0.86 (0.71-1.04) | 0.11 |  | 467 | 1.09 (0.90-1.30) | 0.38 | 0.21 | 36 |
| Fruit and vegetable fibre | 4 | 540 | 0.98 (0.81-1.17) | 0.78 |  | 413 | 1.09 (0.91-1.31) | 0.33 |  | 467 | 0.92 (0.75-1.12) | 0.38 | 0.41 | 0 |
| Fruit fibre | 2 | 540 | 0.99 (0.89-1.10) | 0.83 |  | 413 | 1.00 (0.90-1.12) | 0.98 |  | 467 | 0.98 (0.87-1.11) | 0.77 | 0.97 | 0 |
| Vegetable fibre | 2 | 540 | 0.88 (0.68-1.14) | 0.34 |  | 413 | 1.18 (0.91-1.54) | 0.22 |  | 467 | 0.88 (0.67-1.15) | 0.35 | 0.21 | 37 |
| ^1^ Hazard ratios are adjusted for age (continuous), smoking status and number of cigarettes per day (never smoker, former smoker, current smoker <10 cigs/d, current smoker 10-19 cigs/d, current smoker 20+ cigs/d, unknown; where appropriate), history of diabetes (yes, no, unknown), prior hypertension (yes, no , unknown), prior hyperlipidaemia (yes, no, unknown), Cambridge physical activity index (inactive, moderately inactive, moderately active, active, unknown), employment status (employed or student, not employed or student, unknown), level of education completed (none or primary, secondary, vocational or university, unknown), current alcohol consumption (non-drinkers and sex-specific fifths of intake among drinkers), body mass index (<22.5, 22.5-24.9, 25.0-27.4, 27.5-29.9, ≥30.0 kg/m^2^, unknown), and calibrated intake of energy (continuous), and stratified by sex and EPIC centre. Unit sizes represent approximate differences in mean 24 hour recall intake between participants in the lowest and highest fifths of observed intake.  ^2^ Tests of trend were performed using the calibrated intake (continuous).  ^3^ Tests of heterogeneity of trend by smoking status were obtained assuming independence of risk by smoking status using a meta-analysis method. *I^2^* was estimated as (χ^2^ – degrees of freedom) / χ^2^ x 100.  ^4^ Unavailable for Naples, Heidelberg and Potsdam.  ^5^ Unavailable for Potsdam.  ^6^ Unavailable for Umea.  ^7^ Unavailable for Denmark and Norway. | | | | | | | | | | | | | | |

**Supplementary table 30**: Hazard ratios (95% confidence intervals) for **ischaemic stroke** per unit higher^1^ of calibrated intake of selected animal foods, plant foods, and fibre, **stratified by European region**, showing results from independent subset analyses in the EPIC study.

| Food | Unit sizes (g/day) | Northern (Denmark, Norway, Sweden) | | |  | Central/Southern (all other countries) | | | Measures of heterogeneity^3^ | |
| --- | --- | --- | --- | --- | --- | --- | --- | --- | --- | --- |
|  |  | No. of cases | HR (95% CI) | P for trend^2^ |  | No. of cases | HR (95% CI) | P for trend^2^ | P-het | *I^2^* (%) |
|  |  |  |  |  |  |  |  |  |  |  |
| Red and processed meat | 100 | 2556 | 1.12 (0.89-1.41) | 0.32 |  | 1725 | 1.18 (0.97-1.44) | 0.10 | 0.74 | 0 |
| Red meat | 50 | 2556 | 1.10 (0.92-1.30) | 0.30 |  | 1725 | 1.18 (1.02-1.35) | 0.022 | 0.53 | 0 |
| Processed meat | 50 | 2556 | 1.02 (0.88-1.20) | 0.78 |  | 1725 | 1.07 (0.90-1.28) | 0.42 | 0.68 | 0 |
| Poultry meat | 20 | 2556 | 0.92 (0.79-1.06) | 0.25 |  | 1725 | 1.02 (0.93-1.12) | 0.72 | 0.25 | 25 |
| White fish^4^ | 15 | 2556 | 0.96 (0.89-1.04) | 0.33 |  | 1316 | 0.98 (0.92-1.05) | 0.60 | 0.68 | 0 |
| Fatty fish^5^ | 15 | 2556 | 0.99 (0.92-1.07) | 0.81 |  | 1503 | 1.01 (0.92-1.10) | 0.89 | 0.79 | 0 |
| Milk | 200 | 2556 | 0.96 (0.91-1.02) | 0.18 |  | 1725 | 0.89 (0.82-0.97) | 0.011 | 0.15 | 52 |
| Yogurt | 100 | 2556 | 0.90 (0.83-0.97) | 0.005 |  | 1725 | 0.92 (0.80-1.05) | 0.23 | 0.77 | 0 |
| Cheese | 30 | 2556 | 0.90 (0.80-1.00) | 0.053 |  | 1725 | 0.85 (0.72-1.00) | 0.048 | 0.59 | 0 |
| Eggs^6^ | 20 | 2047 | 1.08 (0.97-1.21) | 0.15 |  | 1725 | 1.00 (0.89-1.14) | 0.95 | 0.37 | 0 |
| Cereals and cereal products | 200 | 2556 | 0.99 (0.80-1.24) | 0.96 |  | 1725 | 0.90 (0.67-1.21) | 0.49 | 0.60 | 0 |
| Fruit and vegetables | 200 | 2556 | 0.80 (0.73-0.88) | <0.0001 |  | 1725 | 0.94 (0.86-1.02) | 0.13 | 0.016 | 83 |
| Fruit | 100 | 2556 | 0.89 (0.83-0.94) | 0.0002 |  | 1725 | 0.96 (0.91-1.01) | 0.087 | 0.049 | 74 |
| Vegetables | 100 | 2556 | 0.81 (0.72-0.92) | 0.0008 |  | 1725 | 0.94 (0.83-1.07) | 0.38 | 0.096 | 64 |
| Legumes^7^ | 20 | 1786 | 0.97 (0.65-1.45) | 0.89 |  | 1725 | 0.97 (0.90-1.05) | 0.43 | 0.99 | 0 |
| Nuts and seeds^6^ | 10 | 2047 | 0.91 (0.65-1.27) | 0.57 |  | 1725 | 1.00 (0.87-1.15) | 0.98 | 0.59 | 0 |
| Total dietary fibre | 10 | 2556 | 0.75 (0.65-0.86) | 0.0001 |  | 1725 | 0.81 (0.69-0.95) | 0.010 | 0.47 | 0 |
| Cereal fibre | 4 | 2556 | 0.94 (0.86-1.02) | 0.11 |  | 1725 | 0.84 (0.75-0.94) | 0.003 | 0.13 | 56 |
| Fruit and vegetable fibre | 4 | 2556 | 0.81 (0.74-0.89) | <0.0001 |  | 1725 | 0.94 (0.86-1.03) | 0.21 | 0.020 | 82 |
| Fruit fibre | 2 | 2556 | 0.90 (0.84-0.95) | 0.0002 |  | 1725 | 0.96 (0.91-1.01) | 0.12 | 0.089 | 65 |
| Vegetable fibre | 2 | 2556 | 0.83 (0.73-0.93) | 0.002 |  | 1725 | 0.97 (0.85-1.11) | 0.66 | 0.072 | 69 |
| ^1^ Hazard ratios are adjusted for age (continuous), smoking status and number of cigarettes per day (never smoker, former smoker, current smoker <10 cigs/d, current smoker 10-19 cigs/d, current smoker 20+ cigs/d, unknown), history of diabetes, prior hypertension, prior hyperlipidaemia (each yes, no, unknown; where appropriate), Cambridge physical activity index (inactive, moderately inactive, moderately active, active, unknown), employment status (employed or student, not employed or student, unknown), level of education completed (none or primary, secondary, vocational or university, unknown), current alcohol consumption (non-drinkers and sex-specific fifths of intake among drinkers), body mass index (<22.5, 22.5-24.9, 25.0-27.4, 27.5-29.9, ≥30.0 kg/m^2^, unknown), and calibrated intake of energy (continuous), and stratified by sex and EPIC centre. Unit sizes represent approximate differences in mean 24 hour recall intake between participants in the lowest and highest fifths of observed intake.  ^2^ Tests of trend were performed using the calibrated intake (continuous).  ^3^ Tests of heterogeneity of trend by European region were obtained assuming independence of risk by European region using a meta-analysis method. *I^2^* was estimated as (χ^2^ – degrees of freedom) / χ^2^ x 100.  ^4^ Unavailable for Naples, Heidelberg and Potsdam.  ^5^ Unavailable for Potsdam.  ^6^ Unavailable for Umea.  ^7^ Unavailable for Denmark and Norway. | | | | | | | | | | |

**Supplementary table 31**: Hazard ratios (95% confidence intervals) for **haemorrhagic stroke** per unit higher^1^ calibrated intake of major foods and fibre, **stratified by European region**, showing results from independent subset analyses in the EPIC study.

| Food | Unit sizes (g/day) | Northern (Denmark, Norway, Sweden) | | |  | Central/Southern (all other countries) | | | Measures of heterogeneity^3^ | |
| --- | --- | --- | --- | --- | --- | --- | --- | --- | --- | --- |
|  |  | No. of cases | HR (95% CI) | P for trend^2^ |  | No. of cases | HR (95% CI) | P for trend^2^ | P-het | *I^2^* (%) |
|  |  |  |  |  |  |  |  |  |  |  |
| Red and processed meat | 100 | 712 | 1.43 (0.93-2.21) | 0.10 |  | 718 | 0.84 (0.59-1.20) | 0.34 | 0.062 | 71 |
| Red meat | 50 | 712 | 1.30 (0.93-1.82) | 0.12 |  | 718 | 0.83 (0.64-1.07) | 0.15 | 0.036 | 77 |
| Processed meat | 50 | 712 | 1.27 (0.95-1.69) | 0.10 |  | 718 | 0.94 (0.69-1.27) | 0.67 | 0.15 | 51 |
| Poultry meat | 20 | 712 | 0.96 (0.72-1.28) | 0.77 |  | 718 | 0.94 (0.80-1.09) | 0.40 | 0.89 | 0 |
| White fish^4^ | 15 | 712 | 1.00 (0.85-1.18) | 0.97 |  | 620 | 1.05 (0.93-1.18) | 0.44 | 0.68 | 0 |
| Fatty fish^5^ | 15 | 712 | 0.92 (0.78-1.08) | 0.30 |  | 665 | 0.98 (0.85-1.14) | 0.81 | 0.55 | 0 |
| Milk | 200 | 712 | 1.08 (0.98-1.18) | 0.13 |  | 718 | 1.05 (0.92-1.20) | 0.44 | 0.80 | 0 |
| Yogurt | 100 | 712 | 0.97 (0.84-1.11) | 0.63 |  | 718 | 0.81 (0.64-1.03) | 0.081 | 0.21 | 38 |
| Cheese | 30 | 712 | 0.95 (0.78-1.17) | 0.63 |  | 718 | 0.81 (0.62-1.04) | 0.10 | 0.32 | 0 |
| Eggs^6^ | 20 | 592 | 1.42 (1.19-1.71) | 0.0002 |  | 718 | 1.07 (0.86-1.32) | 0.56 | 0.042 | 76 |
| Cereals and cereal products | 200 | 712 | 0.94 (0.61-1.43) | 0.76 |  | 718 | 1.20 (0.75-1.92) | 0.45 | 0.44 | 0 |
| Fruit and vegetables | 200 | 712 | 0.91 (0.78-1.08) | 0.28 |  | 718 | 1.08 (0.94-1.23) | 0.30 | 0.13 | 55 |
| Fruit | 100 | 712 | 0.95 (0.86-1.06) | 0.36 |  | 718 | 1.00 (0.93-1.09) | 0.92 | 0.43 | 0 |
| Vegetables | 100 | 712 | 0.91 (0.74-1.13) | 0.41 |  | 718 | 1.16 (0.95-1.42) | 0.15 | 0.11 | 61 |
| Legumes^7^ | 20 | 378 | 0.56 (0.19-1.65) | 0.29 |  | 718 | 1.07 (0.95-1.21) | 0.24 | 0.24 | 28 |
| Nuts and seeds^6^ | 10 | 592 | 0.99 (0.59-1.67) | 0.97 |  | 718 | 0.90 (0.71-1.13) | 0.37 | 0.73 | 0 |
| Total dietary fibre | 10 | 712 | 0.81 (0.63-1.04) | 0.10 |  | 718 | 1.22 (0.95-1.58) | 0.12 | 0.025 | 80 |
| Cereal fibre | 4 | 712 | 0.93 (0.81-1.08) | 0.34 |  | 718 | 0.97 (0.81-1.15) | 0.73 | 0.74 | 0 |
| Fruit and vegetable fibre | 4 | 712 | 0.90 (0.77-1.06) | 0.21 |  | 718 | 1.09 (0.94-1.26) | 0.27 | 0.096 | 64 |
| Fruit fibre | 2 | 712 | 0.96 (0.87-1.06) | 0.42 |  | 718 | 1.02 (0.93-1.11) | 0.72 | 0.40 | 0 |
| Vegetable fibre | 2 | 712 | 0.84 (0.68-1.05) | 0.12 |  | 718 | 1.11 (0.89-1.37) | 0.34 | 0.078 | 68 |
| ^1^ Hazard ratios are adjusted for age (continuous), smoking status and number of cigarettes per day (never smoker, former smoker, current smoker <10 cigs/d, current smoker 10-19 cigs/d, current smoker 20+ cigs/d, unknown), history of diabetes, prior hypertension, prior hyperlipidaemia (each yes, no, unknown; where appropriate), Cambridge physical activity index (inactive, moderately inactive, moderately active, active, unknown), employment status (employed or student, not employed or student, unknown), level of education completed (none or primary, secondary, vocational or university, unknown), current alcohol consumption (non-drinkers and sex-specific fifths of intake among drinkers), body mass index (<22.5, 22.5-24.9, 25.0-27.4, 27.5-29.9, ≥30.0 kg/m^2^, unknown), and calibrated intake of energy (continuous), and stratified by sex and EPIC centre. Unit sizes represent approximate differences in mean 24 hour recall intake between participants in the lowest and highest fifths of observed intake.  ^2^ Tests of trend were performed using the calibrated intake (continuous).  ^3^ Tests of heterogeneity of trend by European region were obtained assuming independence of risk by European region using a meta-analysis method. *I^2^* was estimated as (χ^2^ – degrees of freedom) / χ^2^ x 100.  ^4^ Unavailable for Naples, Heidelberg and Potsdam.  ^5^ Unavailable for Potsdam.  ^6^ Unavailable for Umea.  ^7^ Unavailable for Denmark and Norway. | | | | | | | | | | |

**Supplementary table 32**: Hazard ratios (95% confidence intervals) for **ischaemic stroke** per unit higher^1^ calibrated intake of major foods and fibre, **stratified by** **extent of stroke validation**, showing results from independent subset analyses in the EPIC study.

| Food | Unit sizes (g/day) | Partial validation  (Netherlands, Norway, Sweden, UK) | | |  | Complete validation  (Denmark, Germany, Greece, Italy, Spain) | | | Measures of heterogeneity^3^ | |
| --- | --- | --- | --- | --- | --- | --- | --- | --- | --- | --- |
|  |  | No. of cases | HR (95% CI) | P for trend^2^ |  | No. of cases | HR (95% CI) | P for trend^2^ | P-het | *I^2^* (%) |
|  |  |  |  |  |  |  |  |  |  |  |
| Red and processed meat | 100 | 2504 | 0.98 (0.79-1.20) | 0.81 |  | 1777 | 1.38 (1.11-1.70) | 0.003 | 0.023 | 81 |
| Red meat | 50 | 2504 | 1.02 (0.86-1.20) | 0.86 |  | 1777 | 1.25 (1.09-1.44) | 0.002 | 0.061 | 72 |
| Processed meat | 50 | 2504 | 0.95 (0.81-1.11) | 0.53 |  | 1777 | 1.16 (0.99-1.37) | 0.074 | 0.086 | 66 |
| Poultry meat | 20 | 2504 | 1.02 (0.92-1.12) | 0.72 |  | 1777 | 0.94 (0.82-1.07) | 0.36 | 0.34 | 0 |
| White fish^4^ | 15 | 2504 | 0.94 (0.88-1.02) | 0.13 |  | 1368 | 1.00 (0.94-1.06) | 0.99 | 0.24 | 28 |
| Fatty fish^5^ | 15 | 2504 | 1.00 (0.92-1.08) | 0.95 |  | 1555 | 1.00 (0.92-1.09) | 0.94 | 0.93 | 0 |
| Milk | 200 | 2504 | 0.98 (0.92-1.04) | 0.46 |  | 1777 | 0.91 (0.85-0.98) | 0.007 | 0.15 | 52 |
| Yogurt | 100 | 2504 | 0.90 (0.83-0.98) | 0.014 |  | 1777 | 0.92 (0.83-1.03) | 0.14 | 0.68 | 0 |
| Cheese | 30 | 2504 | 0.87 (0.76-1.00) | 0.051 |  | 1777 | 0.89 (0.78-1.01) | 0.064 | 0.86 | 0 |
| Eggs^6^ | 20 | 1995 | 1.10 (0.98-1.24) | 0.10 |  | 1777 | 1.00 (0.89-1.13) | 0.95 | 0.28 | 15 |
| Cereals and cereal products | 200 | 2504 | 0.99 (0.78-1.26) | 0.93 |  | 1777 | 0.92 (0.71-1.19) | 0.51 | 0.67 | 0 |
| Fruit and vegetables | 200 | 2504 | 0.88 (0.80-0.98) | 0.021 |  | 1777 | 0.87 (0.80-0.94) | 0.0007 | 0.81 | 0 |
| Fruit | 100 | 2504 | 0.93 (0.87-1.00) | 0.046 |  | 1777 | 0.93 (0.89-0.98) | 0.002 | 0.98 | 0 |
| Vegetables | 100 | 2504 | 0.87 (0.76-1.00) | 0.054 |  | 1777 | 0.87 (0.78-0.98) | 0.021 | 0.98 | 0 |
| Legumes^7^ | 20 | 2497 | 1.00 (0.87-1.16) | 0.97 |  | 1014 | 0.96 (0.87-1.04) | 0.31 | 0.57 | 0 |
| Nuts and seeds^6^ | 10 | 1995 | 0.99 (0.84-1.17) | 0.90 |  | 1777 | 0.96 (0.79-1.16) | 0.66 | 0.81 | 0 |
| Total dietary fibre | 10 | 2504 | 0.78 (0.67-0.92) | 0.003 |  | 1777 | 0.76 (0.65-0.88) | 0.0002 | 0.78 | 0 |
| Cereal fibre | 4 | 2504 | 0.85 (0.76-0.94) | 0.002 |  | 1777 | 0.94 (0.86-1.02) | 0.14 | 0.14 | 54 |
| Fruit and vegetable fibre | 4 | 2504 | 0.88 (0.79-0.98) | 0.015 |  | 1777 | 0.87 (0.80-0.95) | 0.001 | 0.90 | 0 |
| Fruit fibre | 2 | 2504 | 0.93 (0.87-1.00) | 0.036 |  | 1777 | 0.93 (0.89-0.98) | 0.004 | 0.99 | 0 |
| Vegetable fibre | 2 | 2504 | 0.89 (0.78-1.02) | 0.093 |  | 1777 | 0.89 (0.79-1.00) | 0.056 | 0.98 | 0 |
| ^1^ Hazard ratios are adjusted for age (continuous), smoking status and number of cigarettes per day (never smoker, former smoker, current smoker <10 cigs/d, current smoker 10-19 cigs/d, current smoker 20+ cigs/d, unknown), history of diabetes, prior hypertension, prior hyperlipidaemia (each yes, no, unknown; where appropriate), Cambridge physical activity index (inactive, moderately inactive, moderately active, active, unknown), employment status (employed or student, not employed or student, unknown), level of education completed (none or primary, secondary, vocational or university, unknown), current alcohol consumption (non-drinkers and sex-specific fifths of intake among drinkers), body mass index (<22.5, 22.5-24.9, 25.0-27.4, 27.5-29.9, ≥30.0 kg/m^2^, unknown), and calibrated intake of energy (continuous), and stratified by sex and EPIC centre. Unit sizes represent approximate differences in mean 24 hour recall intake between participants in the lowest and highest fifths of observed intake.  ^2^ Tests of trend were performed using the calibrated intake (continuous).  ^3^ Tests of heterogeneity of trend by extent of stroke validation were obtained assuming independence of risk for each category using a meta-analysis method. *I^2^* was estimated as (χ^2^ – degrees of freedom) / χ^2^ x 100.  ^4^ Unavailable for Naples, Heidelberg and Potsdam.  ^5^ Unavailable for Potsdam.  ^6^ Unavailable for Umea.  ^7^ Unavailable for Denmark and Norway. | | | | | | | | | | |

**Supplementary table 33**: Hazard ratios (95% confidence intervals) for **haemorrhagic stroke** per unit higher^1^ calibrated intake of major foods and fibre, **stratified by** **extent of stroke validation**, showing results from independent subset analyses in the EPIC study.

| Food | Unit sizes (g/day) | Partial validation  (Netherlands, Norway, Sweden, UK) | | |  | Complete validation  (Denmark, Germany, Greece, Italy, Spain) | | | Measures of heterogeneity^3^ | |
| --- | --- | --- | --- | --- | --- | --- | --- | --- | --- | --- |
|  |  | No. of cases | HR (95% CI) | P for trend^2^ |  | No. of cases | HR (95% CI) | P for trend^2^ | P-het | *I^2^* (%) |
|  |  |  |  |  |  |  |  |  |  |  |
| Red and processed meat | 100 | 789 | 1.09 (0.75-1.59) | 0.65 |  | 641 | 0.95 (0.64-1.41) | 0.80 | 0.61 | 0 |
| Red meat | 50 | 789 | 1.03 (0.76-1.40) | 0.85 |  | 641 | 0.93 (0.71-1.22) | 0.62 | 0.64 | 0 |
| Processed meat | 50 | 789 | 1.19 (0.89-1.59) | 0.25 |  | 641 | 0.99 (0.73-1.35) | 0.96 | 0.40 | 0 |
| Poultry meat | 20 | 789 | 0.94 (0.80-1.11) | 0.45 |  | 641 | 0.94 (0.74-1.20) | 0.62 | 0.99 | 0 |
| White fish^4^ | 15 | 789 | 1.01 (0.86-1.18) | 0.91 |  | 543 | 1.05 (0.93-1.18) | 0.47 | 0.72 | 0 |
| Fatty fish^5^ | 15 | 789 | 0.94 (0.82-1.09) | 0.43 |  | 588 | 0.98 (0.83-1.16) | 0.84 | 0.72 | 0 |
| Milk | 200 | 789 | 1.06 (0.95-1.19) | 0.32 |  | 641 | 1.07 (0.97-1.19) | 0.19 | 0.88 | 0 |
| Yogurt | 100 | 789 | 0.80 (0.67-0.95) | 0.011 |  | 641 | 1.07 (0.92-1.25) | 0.39 | 0.014 | 83 |
| Cheese | 30 | 789 | 0.93 (0.72-1.19) | 0.54 |  | 641 | 0.88 (0.72-1.08) | 0.23 | 0.77 | 0 |
| Eggs^6^ | 20 | 669 | 1.15 (0.93-1.41) | 0.19 |  | 641 | 1.36 (1.13-1.64) | 0.001 | 0.23 | 31 |
| Cereals and cereal products | 200 | 789 | 1.14 (0.72-1.81) | 0.57 |  | 641 | 0.98 (0.64-1.50) | 0.93 | 0.64 | 0 |
| Fruit and vegetables | 200 | 789 | 0.93 (0.78-1.11) | 0.40 |  | 641 | 1.05 (0.92-1.20) | 0.46 | 0.26 | 20 |
| Fruit | 100 | 789 | 0.95 (0.84-1.06) | 0.33 |  | 641 | 1.00 (0.93-1.08) | 0.93 | 0.40 | 0 |
| Vegetables | 100 | 789 | 0.96 (0.75-1.22) | 0.74 |  | 641 | 1.08 (0.90-1.30) | 0.41 | 0.45 | 0 |
| Legumes^7^ | 20 | 762 | 1.12 (1.01-1.24) | 0.026 |  | 334 | 0.96 (0.80-1.15) | 0.65 | 0.14 | 54 |
| Nuts and seeds^6^ | 10 | 669 | 0.75 (0.55-1.01) | 0.058 |  | 641 | 1.12 (0.83-1.50) | 0.46 | 0.062 | 71 |
| Total dietary fibre | 10 | 789 | 1.03 (0.79-1.34) | 0.84 |  | 641 | 0.96 (0.75-1.23) | 0.76 | 0.72 | 0 |
| Cereal fibre | 4 | 789 | 0.99 (0.83-1.18) | 0.89 |  | 641 | 0.94 (0.81-1.08) | 0.36 | 0.64 | 0 |
| Fruit and vegetable fibre | 4 | 789 | 0.93 (0.77-1.11) | 0.40 |  | 641 | 1.04 (0.91-1.19) | 0.57 | 0.31 | 3 |
| Fruit fibre | 2 | 789 | 0.95 (0.85-1.07) | 0.40 |  | 641 | 1.01 (0.94-1.10) | 0.75 | 0.38 | 0 |
| Vegetable fibre | 2 | 789 | 0.88 (0.68-1.13) | 0.32 |  | 641 | 1.02 (0.84-1.23) | 0.85 | 0.36 | 0 |
| ^1^ Hazard ratios are adjusted for age (continuous), smoking status and number of cigarettes per day (never smoker, former smoker, current smoker <10 cigs/d, current smoker 10-19 cigs/d, current smoker 20+ cigs/d, unknown), history of diabetes, prior hypertension, prior hyperlipidaemia (each yes, no, unknown; where appropriate), Cambridge physical activity index (inactive, moderately inactive, moderately active, active, unknown), employment status (employed or student, not employed or student, unknown), level of education completed (none or primary, secondary, vocational or university, unknown), current alcohol consumption (non-drinkers and sex-specific fifths of intake among drinkers), body mass index (<22.5, 22.5-24.9, 25.0-27.4, 27.5-29.9, ≥30.0 kg/m^2^, unknown), and calibrated intake of energy (continuous), and stratified by sex and EPIC centre. Unit sizes represent approximate differences in mean 24 hour recall intake between participants in the lowest and highest fifths of observed intake.  ^2^ Tests of trend were performed using the calibrated intake (continuous).  ^3^ Tests of heterogeneity of trend by extent of stroke validation were obtained assuming independence of risk for each category using a meta-analysis method. *I^2^* was estimated as (χ^2^ – degrees of freedom) / χ^2^ x 100.  ^4^ Unavailable for Naples, Heidelberg and Potsdam.  ^5^ Unavailable for Potsdam.  ^6^ Unavailable for Umea.  ^7^ Unavailable for Denmark and Norway. | | | | | | | | | | |

**References**

1. Haftenberger M, Schuit A, Tormo M, Boeing H, Wareham N, Bueno-de-Mesquita H, Kumle M, Hjartåker A, Chirlaque M, Ardanaz E, Andren C, Lindahl B, Peeters P, Allen N, Overvad K, Tjønneland A, Clavel-Chapelon F, Linseisen J, Bergmann M, Trichopoulou A, Lagiou P, Salvini S, Panico S, Riboli E, Ferrari P, Slimani N. Physical activity of subjects aged 50–64 years involved in the European Prospective Investigation into Cancer and Nutrition (EPIC). *Public Health Nutr* 2002;**5**:1163–1177.

2. Pols MA, Peeters PH, Ocké MC, Slimani N, Bueno-de-Mesquita HB, Collette HJ. Estimation of reproducibility and relative validity of the questions included in the EPIC Physical Activity Questionnaire. *Int J Epidemiol* 1997;**26 Suppl 1**:S181-9.

3. Borch KB, Ekelund U, Brage S, Lund E. Criterion validity of a 10-category scale for ranking physical activity in Norwegian women. *Int J Behav Nutr Phys Act* 2012;**9**:2.

4. Wareham NJ, Jakes RW, Rennie KL, Schuit J, Mitchell J, Hennings S, Day NE. Validity and repeatability of a simple index derived from the short physical activity questionnaire used in the European Prospective Investigation into Cancer and Nutrition (EPIC) study. *Public Health Nutr* 2003;**6**:407–413.

5. Baumeister SE, Schlesinger S, Aleksandrova K, Jochem C, Jenab M, Gunter MJ, Overvad K, Tjønneland A, Boutron-Ruault M-C, Carbonnel F, Fournier A, Kühn T, Kaaks R, Pischon T, Boeing H, Trichopoulou A, Bamia C, Vecchia C La, Masala G, Panico S, Fasanelli F, Tumino R, Grioni S, Bueno de Mesquita B, Vermeulen R, May AM, Borch KB, Oyeyemi SO, Ardanaz E, Rodríguez-Barranco M, Dolores Chirlaque López M, Felez-Nobrega M, Sonestedt E, Ohlsson B, Hemmingsson O, Werner M, Perez-Cornago A, Ferrari P, Stepien M, Freisling H, Tsilidis KK, Ward H, Riboli E, Weiderpass E, Leitzmann MF. Association between physical activity and risk of hepatobiliary cancers: A multinational cohort study. *J Hepatol* European Association for the Study of the Liver; 2019;**70**:885–892.

6. Langenberg C, Sharp S, Forouhi NG, Franks PW, Schulze MB, Kerrison N, Ekelund U, Barroso I, Panico S, Tormo MJ, Spranger J, Griffin S, van der Schouw YT, Amiano P, Ardanaz E, Arriola L, Balkau B, Barricarte A, Beulens JW, Boeing H, Bueno-de-Mesquita HB, Buijsse B, Chirlaque Lopez MD, Clavel-Chapelon F, Crowe FL, de Lauzon-Guillan B, Deloukas P, Dorronsoro M, Drogan D, Froguel P, Gonzalez C, Grioni S, Groop L, Groves C, Hainaut P, Halkjaer J, Hallmans G, Hansen T, Huerta Castaño JM, Kaaks R, Key TJ, Khaw KT, Koulman A, Mattiello A, Navarro C, Nilsson P, Norat T, Overvad K, Palla L, Palli D, Pedersen O, Peeters PH, Quirós JR, Ramachandran A, Rodriguez-Suarez L, Rolandsson O, Romaguera D, Romieu I, Sacerdote C, Sánchez MJ, Sandbaek A, Slimani N, Sluijs I, Spijkerman AM, Teucher B, Tjonneland A, Tumino R, van der A DL, Verschuren WM, Tuomilehto J, Feskens E, McCarthy M, Riboli E, Wareham NJ. Design and cohort description of the InterAct Project: An examination of the interaction of genetic and lifestyle factors on the incidence of type 2 diabetes in the EPIC Study. *Diabetologia* 2011;**54**:2272–2282.

7. Danesh J, Saracci R, Berglund G, Feskens E, Overvad K, Panico S, Thompson S, Fournier A, Clavel-Chapelon F, Canonico M, Kaaks R, Linseisen J, Boeing H, Pischon T, Weikert C, Olsen A, Tjønneland A, Johnsen SP, Jensen MK, Quirós JR, Svatetz CA, Pérez MJ, Larrañaga N, Sanchez CN, Iribas CM, Bingham S, Khaw KT, Wareham N, Key T, Roddam A, Trichopoulou A, Benetou V, Trichopoulos D, Masala G, Sieri S, Tumino R, Sacerdote C, Mattiello A, Verschuren WM, Bueno-de-Mesquita HB, Grobbee DE, van der Schouw YT, Melander O, Hallmans G, Wennberg P, Lund E, Kumle M, Skeie G, Ferrari P, Slimani N, Norat T, Riboli E. EPIC-Heart: The cardiovascular component of a prospective study of nutritional, lifestyle and biological factors in 520,000 middle-aged participants from 10 European countries. *Eur J Epidemiol* 2007;**22**:129–141.

8. Slimani N, Ferrari P, Ocké M, Welch a, Boeing H, Liere M, Pala V, Amiano P, Lagiou a, Mattisson I, Stripp C, Engeset D, Charrondière R, Buzzard M, Staveren W, Riboli E. Standardization of the 24-hour diet recall calibration method used in the european prospective investigation into cancer and nutrition (EPIC): general concepts and preliminary results. *Eur J Clin Nutr* 2000;**54**:900–917.

9. Ferrari P, Day NE, Boshuizen HC, Roddam A, Hoffmann K, Thiébaut A, Pera G, Overvad K, Lund E, Trichopoulou A, Tumino R, Gullberg B, Norat T, Slimani N, Kaaks R, Riboli E. The evaluation of the diet/disease relation in the EPIC study: considerations for the calibration and the disease models. *Int J Epidemiol* 2008;**37**:368–378.
